# A 3-month clofazimine–rifapentine-containing regimen for drug-susceptible tuberculosis versus standard of care (Clo-Fast): a randomised, open-label, phase 2c clinical trial

**DOI:** 10.1016/S1473-3099(25)00436-0

**Published:** 2025-09-04

**Authors:** John Z Metcalfe, Isabelle R Weir, Kimberly K Scarsi, Alberto Mendoza-Ticona, Samuel Pierre, Luke Hall, Jorge Leon-Cruz, Elin M Svensson, Simon E Koele, Wadzanai Samaneka, Cecilia Kanyama, Maxwell Yohane, Neetal Nevrekar, Busisiwe Ntsalaze, Jean Bernard Marc, Melanie Goth, Gary Maartens, Richard Chaisson

**Affiliations:** **Division of Pulmonary and Critical Care Medicine, San Francisco General Hospital and Trauma Center, University of California (UCSF), San Francisco, CA, USA** (Prof J Z Metcalfe MD)**; Center for Biostatistics in AIDS Research in the Department of Biostatistics, Harvard T.H. Chan School of Public Health, Boston, MA, USA** (I R Weir PhD, L Hall MS, J Leon-Cruz MS)**; Department of Pharmacy Practice and Science, College of Pharmacy, University of Nebraska Medical Center, Omaha, NE, USA** (Prof K K Scarsi PharmD)**; Socios En Salud Sucursal Perú, Lima, Peru** (A Mendoza-Ticona MD)**; GHESKIO, Port-au-Prince, Haiti** (S Pierre MD, J B Marc MD)**; Department of Pharmacy, Radboud Institute for Medical Innovation, Radboud University Medical Center, Nijmegen, Netherlands** (E M Svensson PhD, S E Koele PhD)**; Department of Pharmacy, Uppsala University, Uppsala, Sweden** (E M Svensson)**; Milton Park CRS, Harare, Zimbabwe** (W Samaneka MBChB)**; Malawi CRS, Lilongwe, Malawi** (C Kanyama MBBS)**; Blantyre CRS, Blantyre, Malawi** (M Yohane MBBS)**; Byramjee Jeejeebhoy Medical College (BJMC) CRS, Pune, India** (N Nevrekar MD)**; CAPRISA eThekwini CRS, Durban, South Africa** (B Ntsalaze MBChB)**; National Institute of Allergy and Infectious Diseases, Bethesda, MD, USA** (M Goth MD)**; Division of Clinical Pharmacology, Department of Medicine, University of Cape Town, Cape Town, South Africa** (Prof G Maartens MMed)**; The Johns Hopkins University School of Medicine, Baltimore, MD, USA** (Prof R Chaisson MD)

## Abstract

**Background:**

Based on results from preclinical and clinical studies, a five-drug combination of isoniazid, rifapentine, pyrazinamide, ethambutol, and clofazimine was identified with treatment shortening potential for drug-susceptible tuberculosis; the Clo-Fast trial aimed to determine the efficacy and safety of this regimen. We compared 3 months of isoniazid, rifapentine, pyrazinamide, ethambutol, and clofazimine, administered with a clofazimine loading dose, to the standard 6 month regimen of isoniazid, rifampicin, pyrazinamide, and ethambutol in drug-susceptible tuberculosis.

**Methods:**

Clo-Fast was a phase 2c open-label trial recruiting participants at six sites in five countries. Participants aged 18 years or older with pulmonary tuberculosis who were sputum smear positive for acid-fast bacilli or molecular tuberculosis assay positive (with *Mycobacterium tuberculosis* with sensitivity to rifampicin and isoniazid) were eligible for enrolment. Individuals with HIV infection with a CD4^+^ cell count ≥100 cells per mm^3^ could participate. Participants were randomly assigned in a 2:1 ratio (group 1: group 2) or a 2:1:1 ratio (group 1: group 2: group 3), depending on consent to participate in the intensive pharmacokinetic visits required in group 3, using a central web-based system with permuted blocks. The group 1 regimen included 8 weeks of rifapentine–isoniazid–pyrazinamide–ethambutol–clofazimine, with a 2-week 300 mg clofazimine loading dose, followed by 5 weeks of rifapentine–isoniazid–pyrazinamide–clofazimine (13 weeks total). The group 2 control regimen included 8 weeks of isoniazid–rifampicin–pyrazinamide–ethambutol followed by 18 weeks of rifampicin–isoniazid. Group 3 was identical to group 1 over the first 4 weeks of treatment, except that the regimen was administered without a clofazimine loading dose (100 mg daily); after 4 weeks of group 3 treatment, participants transitioned to local standard of care to complete treatment. Group 3 was designed to assess the effect of a 2-week loading dose on clofazimine pharmacokinetics. Randomisation was stratified by HIV status and advanced disease on chest radiograph. The primary efficacy endpoint was time to sputum culture-negative status by 12 weeks. The primary safety endpoint was the proportion of participants experiencing any grade 3 or worse adverse event over 65 weeks. The key secondary endpoint was unfavourable clinical or bacteriological outcomes by week 65. The efficacy analysis population contained participants assigned to groups 1 and 2 who were not late exclusions (no positive culture at screening, entry, or week 1, or if rifampicin resistance or isoniazid resistance was detected at screening or entry); the safety analysis population contained all randomly assigned participants who took at least one dose of treatment. The trial was registered with ClinicalTrials.gov ID: NCT04311502.

**Findings:**

104 participants were randomly assigned to group 1 (n=58), group 2 (n=31), and group 3 (n=15). 82 (79%) were male and 74 (71%) had radiographically advanced disease; 30 (29%) were people with HIV. The trial was stopped early for lack of clinical efficacy. For the primary efficacy outcome, 49 (89%) of 55 group 1 participants and 28 (90%) of 31 group 2 participants had stable sputum culture conversion by week 12 (adjusted hazard ratio 1·21 [90% CI 0·82–1·79]; p=0·2089). Adverse events grade 3 or worse occurred in 26 (45%) of 58 group 1 participants and five (16%) of 31 group 2 participants (difference 30%, 90% CI 14–45; p=0·002). The cumulative probability of a week 65 unfavourable outcome was 52% (95% CI 37–69) in group 1 versus 27% (14–50) in group 2 (p=0·049).

**Interpretation:**

Although the trial was stopped early, we found that a 3-month regimen containing clofazimine and rifapentine had 12-week culture conversion rates that did not differ statistically from the standard of care. The regimen was associated with an unacceptably high proportion of participants with unfavourable composite clinical outcomes and grade 3 or worse adverse events.

## Introduction

The current 6-month standard of care regimen for tuberculosis disease has been in use globally since the 1980s.^1^ A recent trial found that a 4-month rifapentine-based regimen containing moxifloxacin was non-inferior to the standard 6-month regimen.^2^ Shortening effective treatment duration further could reduce time to effective cure, loss to follow-up, and costs incurred by patients.^3^

Clofazimine is a riminophenazine agent that has shown significant intracellular accumulation, a long half-life, anti-inflammatory activity, and low incidence of drug resistance, making it an attractive candidate for tuberculosis treatment.^4^ Clofazimine has demonstrated clinical efficacy in retrospective observational studies,^5–9^ prospective cohorts,^10,11^ and small randomised trials,^12,13^ and is an important component of ongoing tuberculosis treatment trials (eg, NCT05556746 and NCT03828201). Clofazimine contributed significantly to a second-line regimen in BALB/c mice,^14^ and demonstrated high dose-dependent sterilising capability when added to drug-susceptible tuberculosis regimens,^15,16^ exhibiting sustained activity even after stopping treatment.^17^

We aimed to compare the efficacy and tolerability of a 3-month regimen containing a combination of isoniazid, rifapentine, pyrazinamide, ethambutol, and clofazimine with the standard treatment 6 months of isoniazid, rifampicin, pyrazinamide, and ethambutol for drug-susceptible tuberculosis treatment.

## Methods

### Study design and participants

Advancing Clinical Therapeutics Globally (ACTG) A5362 (Clo-Fast) was a randomised, open-label, phase 2c trial evaluating the efficacy and safety of a novel 3-month rifapentine-containing and clofazimine-containing regimen for drug-susceptible pulmonary tuberculosis. The study was done at six outpatient clinics within the ACTG network in Malawi, South Africa, Zimbabwe, India, Haiti, and Thailand.

Participants were adults (aged ≥18 years) with pulmonary tuberculosis, without known isoniazid or rifampicin resistance, diagnosed within 5 days before entry, confirmed by either molecular tuberculosis assay or acid-fast bacilli (AFB) positive sputum, and documentation of a Karnofsky performance score of 50 or higher within 30 days before entry. Individuals with HIV infection with a CD4^+^ cell count of 100 cells per mm^3^ or more could participate. The protocol, with the full list of inclusion and exclusion criteria, is provided in the appendix. A central institutional review board and national and local ethics committees approved the protocol. The ACTG Global Community Advisory Board as well as local community advisory boards at recruiting sites provided input into, and approved, the protocol. All participants gave written informed consent. The trial was registered with ClinicalTrials.gov; NCT04311502.

### Randomisation and masking

Participants were randomly assigned to one of three treatment groups either in a 2:1 ratio (group 1: group 2) or a 2:1:1 ratio (group 1: group 2: group 3), depending on consent to participate in the intensive pharmacokinetic visits required in group 3. Randomised assignment was done using a central web-based system with permuted blocks, balanced across clinical research sites and allowing a maximum imbalance of four participants between group 1 and group 2, or between group 1 and group 3, and two participants between group 2 and group 3. Randomisation was stratified by HIV status and advanced disease on chest radiograph.^18^

### Procedures

The experimental group 1 regimen included 8 weeks of rifapentine–isoniazid–pyrazinamide–ethambutol–clofazimine, with a 2-week 300 mg clofazimine loading dose, followed by 5 weeks of rifapentine–isoniazid–pyrazinamide–clofazimine (13 weeks total). The control group 2 regimen included 8 weeks of isoniazid–rifampicin–pyrazinamide–ethambutol followed by 18 weeks of rifampicin–isoniazid. The experimental group 3 regimen was designed to assess the impact of a 2-week loading dose on clofazimine pharmacokinetics. Group 3 was identical to group 1 over the first 4 weeks of treatment, except that the regimen was administered without a clofazimine loading dose (100 mg daily); after 4 weeks of group 3 treatment, participants transitioned to local standard of care to complete treatment. The medications in each regimen were administered 7 days per week, including at least 5 days of in-person directly observed therapy per week.

In the event of presumptive poor clinical response at week 13 among group 1 participants (ie, persistent symptoms consistent with ongoing active tuberculosis with at least one of the following: lack of radiographic improvement, non-negative week 8 culture result, or week 13 AFB sputum smear-positivity involving at least two separate sputum samples), treatment extension to a maximum of 17 weeks was allowed by protocol. Participants with continued poor clinical response at week 17 were referred to the domestic national tuberculosis programme for standard of care treatment. All decisions regarding treatment extension were required to be discussed with and approved by the A5362 Steering Committee before implementation. Full details of the design and implementation of the trial are provided in the protocol.

All group 3 participants (target, n=20) and the first 20 participants enrolled in group 1 and consenting to participate in intensive pharmacokinetic visits completed a 24 h intensive pharmacokinetic sampling visit at week 2; group 1 participants repeated the intensive sampling visit at week 13. Clofazimine and rifapentine were measured using validated, quality-controlled liquid chromatography-tandem mass spectrometry assays at the Division of Clinical Pharmacology, University of Cape Town, Cape Town, South Africa. Non-compartmental pharmacokinetic analyses were performed in R version 4.1.3 with the NCAPPC package version 0.3.0.^19^ Group 1 and 3 pharmacokinetic parameters were compared at week 2 by the Wilcoxon rank-sum test.

### Outcomes

The trial had co-primary efficacy and safety outcome measures. The primary efficacy outcome was time to stable liquid culture conversion up to 12 weeks, defined as the first of two negative sputum cultures without an intervening positive culture. The primary safety outcome was the proportion of participants experiencing any grade 3 or worse adverse event over 65 weeks. Adverse events were graded according to the Division of AIDS Adverse Event Grading Table (version 2.1), except for QT-interval prolongation which was graded according to protocol-specific criteria (appendix p 79).

Unfavourable clinical and bacteriological outcomes up to 65 weeks were defined as the following: culture-positivity at or after end of treatment, confirmed by a second sample; death, except from violent or accidental cause; a positive culture for *Mycobacterium tuberculosis* when last seen; or extension of treatment beyond 14 days of the nominal level (week 13 for group 1 and week 26 for group 2), except to make up for missed doses. Participants were unevaluable if they were either lost to follow-up with their last culture being negative for *M tuberculosis* or died due to violent or accidental cause. In order to provide a detailed assessment of clofazimine-associated hyperpigmentation, we also assessed participant-reported changes in skin pigment and distress related to these changes, patient reported quality-of-life (WHO Quality of Life-BREF), and depression (Center for Epidemiologic Studies Depression Scale). Other secondary outcomes were proportion of participants who had a tuberculosis recurrence; tolerability, estimated as the proportion with premature treatment discontinuation up to 65 weeks (discontinuation other than due to violent death, natural disaster, or administrative censoring); proportion of participants with one or more serious adverse events up to week 65; time to positivity in liquid culture (mycobacteria growth indicator tube); difference in improvement in radiographic score;^20^ mean change and absolute QTcF; and plasma pharmacokinetic parameters for clofazimine.

### Statistical analysis

The trial was designed to detect a difference in the primary microbiological efficacy outcome between group 1 and group 2 using a one-sided alpha level of 0·05. We estimated that a sample size of 165 would provide 90% power to test the hypothesis that there was a difference in time to 12-week culture conversion between group 1 and group 2, assuming a 70% culture conversion rate in group 2 and a hazard ratio of 1·8. For the primary efficacy analysis, we used a Cox proportional hazards model adjusted for HIV status and presence of advanced radiographic disease to estimate the hazard ratio and corresponding 90% CI.

The primary microbiological efficacy and secondary efficacy analyses were performed for all participants randomly assigned to groups 1 and 2 who were not late exclusions. Participants were excluded after randomisation (ie, late exclusions) if they did not have any positive culture at screening, entry, or week 1, or if rifampicin resistance or isoniazid resistance was detected at screening or entry. We estimated the difference in cumulative proportions of unfavourable outcomes up to week 65 between group 1 and group 2 using a Kaplan–Meier estimator and associated 90% CIs. Differences in radiographic score from baseline to end of treatment between group 1 and group 2 in the efficacy set were assessed using linear regression, adjusting for HIV status and advanced disease status. The primary safety analysis included all randomly assigned participants who started study treatment. Non-compartmental pharmacokinetic analyses were performed in R version 4.1.3 with the NCAPPC package version 0.3.0.^19^

An independent Data and Safety Monitoring Board (DSMB) reviewed safety and efficacy data throughout the trial. Oversight and responsibility for data management were delegated by the sponsor to Frontier Sciences Foundation. An independent Review Committee, distinct from the DSMB, adjudicated all clinical unfavourable endpoints that were not bacteriologically confirmed. Interim monitoring guidelines were established for QT prolongation, futility, unfavourable clinical or bacteriological efficacy, and early recurrence (protocol, section 10.5.1).

### Role of the funding source

The trial sponsors were responsible for the design and conduct of the trial. The sponsors of the study had no role in data collection, data analysis, data interpretation, or writing of the report.

## Results

**Figure 1:**
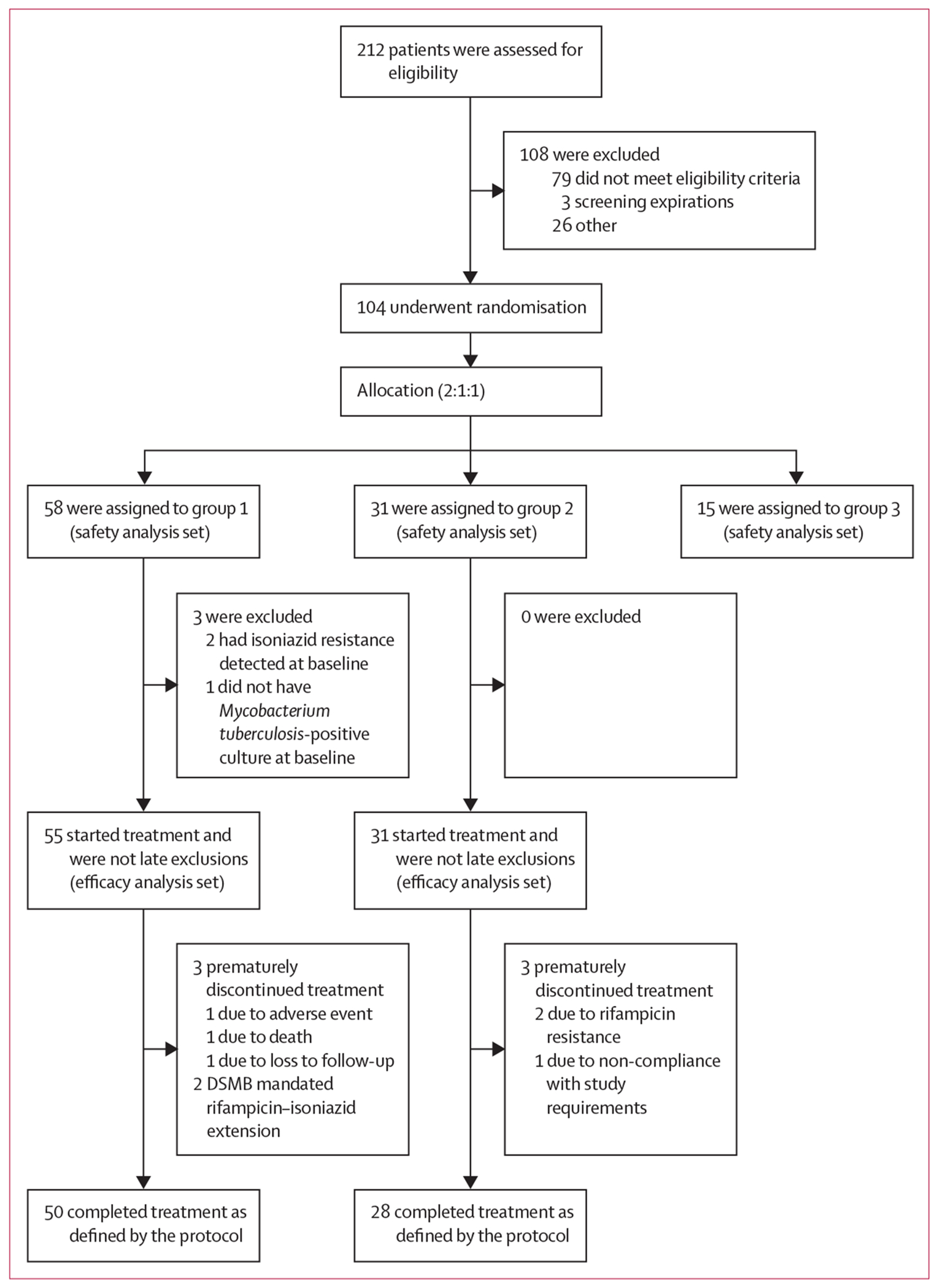
Trial profile The efficacy analysis set contained participants randomly assigned to groups 1 and 2 who were not late exclusions; the safety analysis set contained all randomly assigned participants who had at least one dose of treatment. Group 1=8 weeks of isoniazid, rifapentine, pyrazinamide, ethambutol, and clofazimine with a 2-week loading dose, followed by 5 weeks of rifapentine, isoniazid, pyrazinamide, and clofazimine (13 weeks total treatment). Group 2=standard of care isoniazid, rifampicin, pyrazinamide, and ethambutol. Group 3=isoniazid, rifapentine, pyrazinamide, ethambutol, and clofazimine once daily for 4 weeks, without a clofazimine loading dose, then isoniazid, rifampicin, pyrazinamide, and ethambutol according to local standard of care to complete 6 months of treatment. DSMB=Data and Safety Monitoring Board.

Between Nov 1, 2021, and June 2, 2023, 212 participants were screened and 104 were randomly assigned (58 to group 1, 31 to group 2, and 15 to group 3; figure 1). At a planned interim analysis in March, 2023, the trial met prespecified stopping criteria for lack of clinical or bacteriological efficacy with no safety concerns. Trial accrual was paused in April, 2023, and subsequently stopped for lack of efficacy in June, 2023, at the recommendation of the DSMB. Participants in group 1 who were still on study therapy when the study was stopped completed an additional 3 months of isoniazid and rifampicin. This report includes results for all follow-up to the earliest of week 65, premature discontinuation before week 65, or Sept 25, 2023, when the last participant completed study treatment.

Three participants in each group who prematurely discontinued treatment, and two participants assigned to group 1 who underwent DSMB-recommended treatment extension, were unevaluable for week 65 outcomes. 82 (79%) participants were male, 30 (29%) were people with HIV, 26 (25%) had AFB smear grade 3+, and 74 (71%) had advanced disease on chest radiograph at baseline (table 1). Compared with group 2, group 1 participants had a higher proportion of males (47 [81%] *vs* 22 [71%] of 58), AFB smear grade 3+ (18 [31%] of 58 *vs* five [16%] of 31), and, for people with HIV, had a lower median CD4^+^ count at baseline. Among the 22 participants with HIV and data on antiretroviral regimen, 14 (64%) were taking tenofovir–lamivudine–dolutegravir with an additional 50 mg dose of dolutegravir, and six (27%) were taking tenofovir–lamivudine–dolutegravir without additional dolutegravir. The median duration of trial participation was 50 weeks (IQR 40–58).

**Figure 2:**
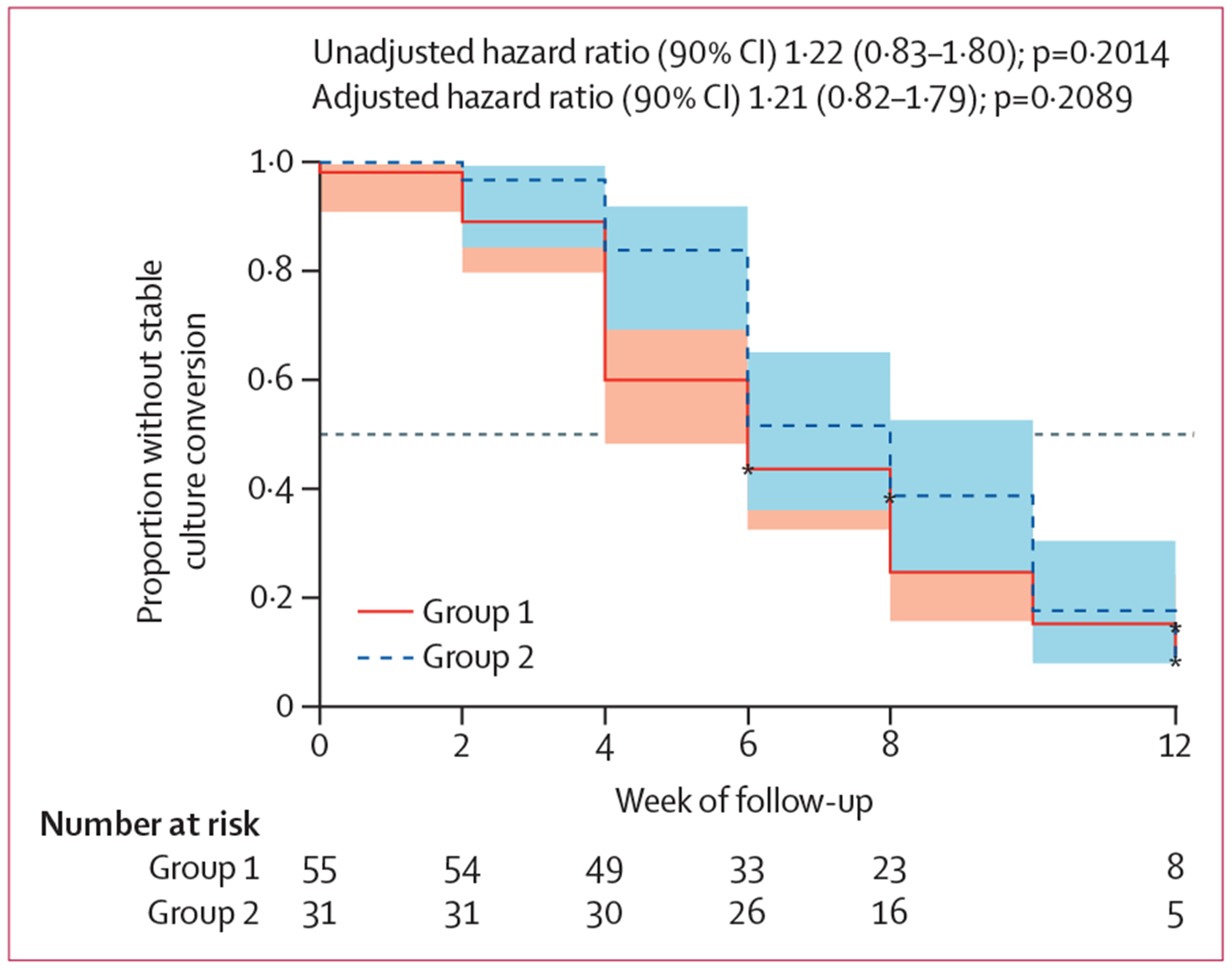
Primary efficacy analysis of time to culture-negative status by 12 weeks The line and shaded portion represent the estimate and corresponding 90% CIs, respectively, for each treatment group; asterisks represent censored participants. Group 1=8 weeks of isoniazid, rifapentine, pyrazinamide, ethambutol, and clofazimine with a 2-week loading dose, followed by 5 weeks of rifapentine, isoniazid, pyrazinamide, and clofazimine (13 weeks total treatment). Group 2=standard of care isoniazid, rifampicin, pyrazinamide, and ethambutol.

86 (97%) of 89 participants were included in the efficacy analyses; three participants were late exclusions due to isoniazid resistance (n=2) or culture-negativity (n=1) at enrolment. Of these, 80 (93%) participants (including two with DSMB-mandated treatment extension; 52 in group 1; 28 in group 2) completed their respective 13-week or 26-week treatment period. For the primary efficacy outcome, 49 (89%) of 55 participants in group 1 and 28 (90%) of 31 participants in group 2 had stable culture conversion by week 12 (adjusted hazard ratio 1·21 [90% CI 0·82–1·79]; p=0·2089; figure 2). Culture conversion occurred by 8 weeks in 41 (75%) of 51 participants in group 1 and 19 (61%) of 31 participants in group 2 (1·46 [0·92 to 2·31]; figure 2). Median time to negative culture was 6 weeks (IQR 4–8) in group 1 and 8 weeks (6–10) in group 2. Mycobacteria growth indicator tube time-to-positivity increased linearly on a log10 scale over 12 weeks in groups 1 and 2 without a statistically significant difference in slope between groups (appendix pp 2, 11).

**Figure 3:**
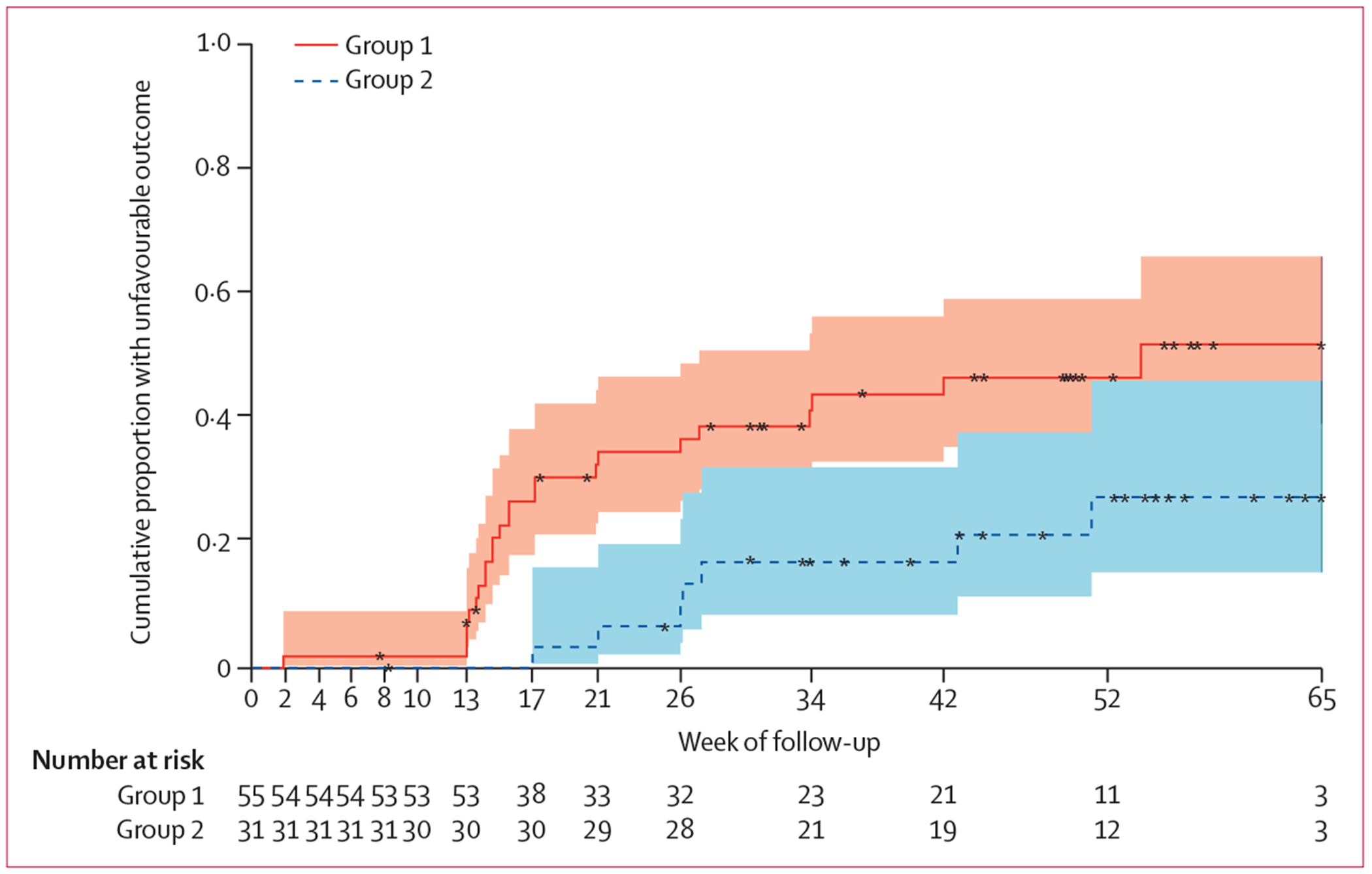
Time to unfavourable outcome up to week 65 The line and shaded portion represent the Kaplan–Meier estimate and corresponding 90% CIs, respectively. Group 1=8 weeks of isoniazid, rifapentine, pyrazinamide, ethambutol, and clofazimine with a 2-week loading dose, followed by 5 weeks of rifapentine, isoniazid, pyrazinamide, and clofazimine (13 weeks total treatment). Group 2=standard of care isoniazid, rifampicin, pyrazinamide, and ethambutol.

At week 65, the cumulative probability of an unfavourable outcome was 52% (95% CI 37–69) in group 1 compared with 27% (14–50; p=0·049) in group 2 (figure 3; appendix p 3). Among the unfavourable outcomes, 12 (50%) of 24 participants in group 1 and three (43%) of seven participants in group 2 were confirmed by culture (ie, two or more positive cultures at or after week 13 or week 17, respectively, or culture-positive when last seen), and 11 (46%) in group 1 and four (57%) in group 2 had treatment extension due to clinically inadequate response (appendix p 12). Of culture-confirmed unfavourable outcomes, nine (75%) of 12 in group 1 and one (33%) of three in group 2 occurred following end of treatment and were considered tuberculosis recurrences (appendix pp 3, 12). Unfavourable outcomes related to treatment extension in the absence of culture confirmation were concordantly adjudicated by the Independent Review Committee.

By the end of each respective treatment duration, controlling for HIV status and baseline radiographic severity, group 1 participants were less likely to have radiographic improvement than those in group 2 (mean improvement in chest x-ray score 26 [95% CI 34 to 17] *vs* 46 [58 to 34]; p<0·01), and were more likely to have residual pulmonary cavitations (18 [33%] of 55 *vs* seven [23%] of 31; appendix p 4). Improvement in chest x-ray score across the first 13 weeks of treatment did not significantly differ between groups (mean difference 10 [95% CI −4 to 25], favouring control; p=0·15).

*M tuberculosis* acquired resistance to isoniazid among two participants in group 1; one participant was removed from the trial and treated for isoniazid-resistant tuberculosis (unfavourable outcome), and another clinically improved without recurrence (favourable outcome).

Up to week 65, the cumulative proportion of participants experiencing a grade 3 or worse adverse event was greater in group 1 (26 [45%] of 58; 90% CI 36–58) than in group 2 (five [16%] of 31; 8–31); the estimated cumulative difference was 30% (90% CI 14–45; p=0·002; table 2, appendix p 5). A higher cumulative proportion of serious adverse events occurred in group 1 (eight [14%]) than group 2 (one [3%]; estimated percentage-point difference 11 [90% CI 1–20]; p=0·06). Tolerability, estimated as permanent premature discontinuation of study treatment, did not differ between groups (two [4%] of 58 in group 1 *vs* three [10%] of 31 in group 2; p=0·35). Increased creatinine was the most common reported grade 3 or worse adverse event in both groups 1 and 2. 13 (22%) of 58 participants in group 1 and three (10%) of 31 participants in group 2 experienced grade 3 or worse increases in creatinine; all such events reflected an increase in creatinine of 1·5 to <2·0-times the baseline. The mean decrease in estimated glomerular filtration rate nadir during the study period was −12·5 mL/min (SD 13·6) in group 1 versus −3·4 mL/min (13·1) in group 2. Five (9%) of 58 participants in group 1 and one (7%) of 15 participants in group 3 experienced hepatic adverse events of grade 3 or worse. One event was solely due to bilirubin increase, and one event met Hy’s Law criteria, both in group 1. The mean increase in the electrocardiographic QTcF interval up to week 13 was 32 ms (95% CI 26–37) in group 1 and 8 ms (1–15) in group 2 (appendix p 6). QTcF prolongation of 60 ms or more above baseline occurred in six (10%) of 58 participants in group 1 and no participants in group 2. No participants in either group had a QTcF interval more than 480 ms at any study visit. Participant subjective assessment of skin hyperpigmentation and distress was higher in group 1 than group 2 at week 13, though neither measure differed from group 2 by week 26 (appendix pp 7–8).

Before the study was stopped, 17 participants in group 1 and 15 participants in group 3 completed all intensive pharmacokinetic visits (appendix pp 9–10). The 2-week clofazimine loading period significantly increased exposure, resulting in over three-fold higher clofazimine maximum concentration (geometric mean ratio 3·5 [90% CI 2·7–4·5]), minimum concentration (3·1 [2·4–4·1]), and area under the curve (AUC)_24h_ (3·2 [2·5–4·2]) at week 2 in group 1 compared with group 3. In group 1, clofazimine concentrations at week 2 were similar to week 13 (appendix p 13). Rifapentine exposure was similar between groups 1 and 3, and between week 2 and week 13 in group 1 (appendix pp 9–10, 14).

## Discussion

In this randomised controlled trial, a 3-month regimen of rifapentine–isoniazid–pyrazinamide–ethambutol–clofazimine had poor clinical efficacy for the treatment of drug-susceptible pulmonary tuberculosis. The primary analysis of time to stable conversion of sputum cultures to negative demonstrated no significant improvement versus standard of care, and secondary efficacy analyses including improvement in chest radiographic score were also not supportive. The incidence of grade 3 or worse adverse events was increased in group 1, though treatment discontinuation was similar across treatment groups.

Participants receiving the experimental clofazimine-containing and rifapentine-containing regimen demon strated adjusted 12-week liquid culture conversion rates not significantly different than the control regimen and similar to the Study 31/A5349 moxifloxacin-containing and rifapentine-containing regimen found to be non-inferior to standard of care.^2^ Further, the log10 slope of mycobacteria growth indicator tube time-to-positivity, increasingly used in phase 2 trials,^21^ was statistically indistinguishable from control. However, 65-week unfavourable outcomes, driven by a high proportion of participants with microbiologically-confirmed recurrence, were dramatically higher than in these previous trials and suggest unacceptably low rates of *M tuberculosis* sterilisation.

The absence of clinical efficacy in Clo-Fast is likely multifactorial. First, as early as 1959, Barry and colleagues questioned whether the lack of clofazimine distribution to extracellular *M tuberculosis* within pulmonary cavities could impede its sterilisation efficacy and prevention of relapse,^22^ and more recent experiments support this concern.^23,24^ Second, the high proportion of trial participants with advanced radiographic disease on chest x-ray, high-grade smear-positivity, and living with HIV infection (29%, *vs* 8% for Study 31/A5349),^2^ as well as the high rate of unfavourable outcomes in the control group 2, indicate that the recruited participants might have had difficult-to-treat tuberculosis disease at study entry. Clo-Fast recruitment also occurred during the COVID-19 pandemic as lockdowns eased, which could have resulted in delayed access to care and more advanced disease presentations. Third, a 3-month treatment duration, unassessed in randomised trials since before the HIV era,^25^ remains a bold and challenging target when considering the full spectrum of tuberculosis disease severity. Fourth, a drug-susceptible versus drug-resistant tuberculosis companion regimen might have been less favourable to clofazimine. Isoniazid-susceptible, catalase-positive *M tuberculosis* might be less susceptible to clofazimine^26^ due to a relatively greater ability to clear reactive oxygen species,^27^ considered to be central to clofazimine’s mechanism of action.^28^

Clo-Fast found that clofazimine over a 3-month treatment duration performs poorly in the context of disease severity where the relative proportion of extracellular to intracellular *M tuberculosis* is likely to be high, with widespread necrotic foci and cavitation (for which plain radiographs have poor sensitivity). The scarcity of sterilising activity was not offset by the use of pyrazinamide through the full 3-month duration and the substitution of rifampicin for rifapentine. Clofazimine efficacy during later stages of treatment, longer treatment durations, and/or in the context of lesser lung pathology may differ. In addition, the target plasma concentration of clofazimine (0·25 mg/L), extrapolated from mice,^17^ was surpassed in Clo-Fast early in treatment; the poor efficacy in Clo-Fast suggests that the murine bioequivalent dose in humans might be considerably higher for clofazimine than previously thought.

The use of a clofazimine loading dose was associated with a three-fold increase in drug exposure and faster achievement of (though without exceeding) week 13 concentrations. Given that the WHO recommended 100 mg once-daily dose of clofazimine without a loading dose is an historical phenomenon inherited from the leprosy field,^29^ our data support using a clofazimine loading dose as standard practice within tuberculosis treatment regimens to optimise clofazimine plasma concentrations.

Clo-Fast was designed to provide a detailed assessment of clofazimine-associated hyperpigmentation, patient distress in response to hyperpigmentation, and quality of life. Although it was hoped that the short duration of treatment would minimise these effects, a small number of individuals experienced significant hyperpigmentation and distress even up to 65 weeks, as described with longer-term treatment.^30^ This effect also being present in a similar number of controls might reflect nocebo or expectation bias. Clofazimine had relatively potent QTc effects driven by its concentration, as shown in previous studies;^31^ however, QTc increases above clinically relevant thresholds (ie, 480 ms) did not occur. Finally, decreases in estimated glomerular filtration rate were an unexpected finding, previously unreported with clofazimine. This finding might simply represent chromogen interference with the creatinine assay, and might therefore have limited clinical significance.

This trial has limitations. First, the early termination of Clo-Fast decreases its statistical power; secondary endpoints, in particular, should be interpreted with caution due to reduced sample size. However, primary and secondary outcomes were concordant, and the trial had high internal validity. Second, because of the open-label nature of the trial, the short treatment duration, and potential negative predisposition to clofazimine (among site clinicians and/or participants), ascertainment bias might have increased unfavourable treatment-outcome assignment in group 1 among cases not microbiologically confirmed. However, we found similar differences using culture-confirmed events or events based on treatment extensions without culture confirmation, suggesting that ascertainment bias was not a significant contributor. In addition to a standardised approach for collection of objective data to support outcome determination, an Independent Review Committee was convened in the Clo-Fast trial to adjudicate unfavourable outcomes that were not confirmed by culture results. Nevertheless, given that ascertainment bias is difficult to measure or control, future tuberculosis treatment shortening trials should optimise rigour through use of placebo controls, if at all feasible.

In conclusion, a 3-month rifapentine–clofazimine regimen had high unfavourable outcomes. Using the available preclinical evidence, generated in both BALB/c^15,16^ and Kramnik^32^ mice, we were unable to accurately predict sterilising activity and risk of relapse in humans. This result illustrates the ongoing challenge of successfully translating preclinical and clinical evidence into successful treatment-shortening trials.

## Supplementary Material

Appendix

## Figures and Tables

**Table 1:** Baseline characteristics

	Group 1 (n=58)	Group 2 (n=31)	Group 3 (n=15)
Age, years	33 (27–39)	30 (24–38)	35 (31–43)

Sex*			
Female	11 (19%)	9 (29%)	2 (13%)
Male	47 (81%)	22 (71%)	13 (87%)

Race			
Asian	9 (16%)	6 (19%)	0
Black	49 (84%)	25 (81%)	15 (100%)

Country†			
Haiti	2 (3%)	2 (6%)	0
India	9 (16%)	5 (16%)	0
Malawi	26 (45%)	11 (35%)	9 (60%)
South Africa	11 (19%)	6 (19%)	3 (20%)
Zimbabwe	10 (17%)	7 (23%)	3 (20%)

BMI, kg/m^2^	19 (17–20)	18 (17–21)	18 (17–20)

HIV positivity	16 (28%)	9 (29%)	5 (33%)

CD4 count among people with HIV positivity	237 (182–347)	301 (262–468)	226 (174–318)

Advanced radiographic disease on chest x-ray‡	40 (69%)	21 (68%)	13 (87%)

Diabetes§	5 (9%)	2 (6%)	2 (13%)

Karnofsky Performance Scale¶			
Karnofsky score 50–70%	8 (14%)	5 (16%)	2 (13%)

Karnofsky score 80–100%	50 (86%)	26 (84%)	13 (87%)

WHO AFB smear grade			
Negative	11 (19%)	4 (13%)	1 (7%)
Scanty or 1–9 AFB	8 (14%)	5 (16%)	1 (7%)
1+	5 (9%)	6 (19%)	3 (20%)
2+	16 (28%)	11 (35%)	7 (47%)
3+	18 (31%)	5 (16%)	3 (20%)

Median time to MGIT positivity at baseline, days‖	6·2 (3·8–7·7)	5·7 (4·0–8·1)	··

Data are n (%) or median (IQR). AFB=acid-fast bacilli. Group 1=8 weeks of isoniazid, rifapentine, pyrazinamide, ethambutol, and clofazimine with a 2-week loading dose, followed by 5 weeks of rifapentine, isoniazid, pyrazinamide, and clofazimine (13 weeks total treatment). Group 2=isoniazid, rifampicin, pyrazinamide, and ethambutol. Group 3=isoniazid, rifapentine, pyrazinamide, ethambutol, and clofazimine without a loading dose. MGIT=mycobacteria growth indicator tube.

* Participants’ gender was completely concordant with sex at birth.

† Participants were screened for enrolment but not enrolled at Chiang Mai, Thailand.

‡ Radiographically advanced disease was defined as a total combined area of pulmonary opacity across all lung fields equivalent to or greater than one entire lung, or one or more large cavitations (≥3 cm).

§ HbA_1c_ and fasting glucose were missing for three participants in group 2.

¶ Scores above 70% suggest a patient is able to care for themselves independently; scores between 50% and 70% suggest that a patient is unable to work, with varying amount of assistance needed; scores below 50% indicate significant disability, requiring considerable medical assistance.

‖ Time to positivity was missing for three participants in group 1.

**Table 2:** Safety and premature treatment discontinuation

	Group 1 (n=58)	Group 2 (n=31)	Group 3 (n=15)
**Primary safety outcome**			
Adverse event of grade 3 or worse, n (%)	26 (45%)	5 (16%)	3 (20%)
Percentage-point difference from control (90% CI)	30 (14 to 45)	··	··

**Secondary safety outcomes**			
Any serious adverse event, n (%)	8 (14%)	1 (3%)	2 (13%)
Percentage-point difference from control (90% CI)	11 (1 to 20)	··	··
Permanent premature treatment discontinuation, n (%)*	2 (4%)	3 (10%)	0
Percentage-point difference from control (95% CI)	−6 (−22 to 5)	··	··

**Other safety outcomes**			
Any adverse event leading to death, n (%)†	1 (2%)	0	1 (7%)
Any liver-related adverse event grade 3 or above, n (%)	5 (9%)	0	1 (7%)
Hy’s Law criteria met, n (%)‡	1 (2%)	··	··
Any renal glomerular filtration rate-related adverse event grade 3 or worse, n (%)§	13 (22%)	3 (10%)	0
Any QTc-related adverse event grade 3 or worse, n (%)¶	0	0	0

The safety analysis population included all participants who had undergone randomisation and received at least one dose of the assigned treatment. Adverse events were graded by the site investigators according to the Division of AIDS Table for Grading the Severity of Adult and Pediatric Adverse Events. Participants with multiple adverse events in each category are counted only once in each category. One participant with a liver-related adverse event grade 3 or worse met these criteria due to bilirubin elevation alone. Group 1=8 weeks of isoniazid, rifapentine, pyrazinamide, ethambutol, and clofazimine with a 2-week loading dose, followed by 5 weeks of rifapentine, isoniazid, pyrazinamide, and clofazimine (13 weeks total treatment). Group 2=standard of care isoniazid, rifampicin, pyrazinamide, and ethambutol. Group 3=isoniazid, rifapentine, pyrazinamide, ethambutol, and clofazimine without a loading dose. ULN=upper limit of normal.

* Defined as premature treatment discontinuation up to week 65 other than due to violent death, natural disaster, or administrative censoring.

† Adverse events in this category were reported regardless of attribution. In group 3, one participant death was due to underlying congestive heart failure, cor pulmonale, venous thrombosis, and respiratory failure, deemed by site investigators to be unlikely related to tuberculosis treatment. In group 3, one participant death was due to progression of tuberculosis disease and immunosuppression.

‡ Alanine aminotransferase or aspartate aminotransferase more than 3 × ULN, total bilirubin more than 2 × ULN, and alkaline phosphatase less than 2 × ULN.

§ Glomerular filtration rate-related adverse events can be classified as grade 3 based on an increase in creatinine to 1·5 to <2·0 times baseline, or an increase of more than 1·8 times the ULN; all events in groups 1 and 2 reflected the former criterion and none of the latter.

¶ Protocol-specific criteria for an adverse event grade 3 included QTcF more than 500 ms, or QTcF more than 480 ms and QTcF change from baseline more than 60 ms; six individuals in group 3 had a QTcF change from baseline more than 60 ms, but no participant randomly assigned to any study group had a QTcF more than 480 ms.

## Data Availability

Due to ethical restrictions, study data are available upon request from https://actgnetwork.org/submit-a-proposal-2/ with the written agreement of ACTG.

## Research in context

### Evidence before this study

We searched PubMed for clinical trials of tuberculosis drug regimens published between Jan 1, 1970, and Jan 1, 2025 using the MeSH terms “clofazimine” AND “rifapentine”. Treatment regimens containing clofazimine and rifapentine have been studied singularly and in different combinations. Rifapentine when combined with moxifloxacin in a 4-month regimen (Study 31/A5349) was non-inferior to the 6-month standard of care. A large body of observational literature suggests that clofazimine is efficacious in treatment of tuberculosis, and murine models suggested that a 3-month drug-susceptible tuberculosis regimen combining clofazimine and rifapentine would be highly sterilising in humans. Multiple randomised trials have included clofazimine as a component in 6-month (TB PRACTECAL stage 1) or 9-month (STREAM stages 1 and 2; endTB) rifampicin-resistant tuberculosis regimens and were non-inferior to control groups. A treatment strategy trial (TRUNCATE) using an 8–12-week high-dose rifampicin, clofazimine, pyrazinamide, and ethambutol regimen found that 13% of participants had an unfavourable outcome (a definition that included early relapse with successful retreatment) at 96 weeks. A randomised trial specifically evaluating clofazimine (100 mg daily for 21 months *vs* placebo) within an optimised multidrug-resistant tuberculosis background regimen found significantly faster time to culture conversion and healing of cavities on chest computed tomography, and higher treatment success (39 [73·6%] of 53 *vs* 28 [53·8%] of 52; p=0·035) in patients randomly assigned to clofazimine; relapse was not assessed. In contrast, a second randomised clinical trial evaluating clofazimine as part of individualised extensively drug-resistant tuberculosis regimens over 36 months found no difference in time to sputum culture conversion on solid media; of note, no participant discontinued therapy because of adverse events in the clofazimine group of either trial. Results from a phase 2a early bactericidal activity study showed no antimicrobial effect of clofazimine during 14 days of therapy.

The results of a phase 2c clinical trial (NCT03561753), which were not available at the start of this study, assessing a 4-month regimen of clofazimine (150 mg daily), protionamide, pyrazinamide, and ethambutol, found no difference in 2-month culture conversion (primary outcome) compared with standard of care but lower rates of treatment success, largely due to an elevated rate of drug-induced hepatitis. Randomised controlled trials assessing curative 3-month tuberculosis treatment regimens have not been reported since the 1980s.

### Added value of this study

A 3-month rifapentine-containing and clofazimine-containing regimen for treatment of drug-susceptible tuberculosis had 12-week culture conversion rates that did not differ statistically from the standard of care but was associated with an unacceptably high proportion of participants with unfavourable composite clinical outcomes. Until week 65, grade 3 or worse adverse events were more frequent in the experimental group (26 [45%] of 58) than in the control group (five [16%] of 31; p=0·002). Premature discontinuation of study treatment did not differ between groups. A 2-week loading dose of clofazimine increased drug exposure three-fold with a faster time to near steady state, and was well-tolerated.

### Implications of all the available evidence

A 3-month clofazimine-containing and rifapentine-containing regimen for drug-susceptible tuberculosis, with pyrazinamide throughout, has poor treatment efficacy for drug-susceptible tuberculosis, and grade 3 or worse adverse events were more frequent than in the control group. Clofazimine efficacy during later stages of treatment, longer treatment durations, and/or in the context of lesser lung pathology might differ. Using the available preclinical evidence, generated in both BALB/c and Kramnik mice, we were unable to accurately predict sterilising activity and risk of relapse in humans. Successfully translating preclinical and clinical evidence into successful treatment-shortening trials is an ongoing challenge for the field.
