# Supplementary material for: A 3-month clofazimine–rifapentine-containing regimen for drug-susceptible tuberculosis versus standard of care (Clo-Fast): a randomised, open-label, phase 2c clinical trial
Source: Lancet Infect Dis. Author manuscript; Available in PMC 2026 Mar 16. (PMC12990188; doi:10.1016/S1473-3099(25)00436-0)
Supplement: Appendix [file NIHMS2145898-supplement-Appendix.pdf]

THE LANCET
Infectious Diseases

Supplementary appendix

This appendix formed part of the original submission and has been peer reviewed. We post it as supplied by the authors.

Supplement to: Metcalfe JZ, Weir IR, Scarsi KK, et al. A 3-month clofazimine–rifapentine-containing regimen for drug-susceptible tuberculosis versus standard of care (Clo-Fast): a randomised, open-label, phase 2c clinical trial. *Lancet Infect Dis* 2025; published online Sept 4. https://doi.org/10.1016/S1473-3099(25)00436-0.

Supplementary appendix

Table of contents

Figure S1. Time-to-positivity (TTP) data used for slope analysis, illustrated per arm……………………….2
Figure S2. Time to unfavorable clinical/bacteriologic tuberculosis composite outcome through week 65…3
Figure S3. Mean Chest X-ray Score Over Time by Arm…………………………………………………4
Figure S4. Time to first grade ≥3 adverse event through week 65..………………………………………5
Figure S5. Mean QTcF interval change through 13 weeks……………………………………………..6
Figure S6. Subjective Change in Skin Coloration and Subjective Distress Caused by Change in Skin Coloration by Arm…………………………………………………………………………………..7
Figure S7. Median clofazimine (a) and rifapentine (b) concentration-time-curve over 24 hours after an observed dose………………………………………………………………………………………9
Table S1. Final TPP pharmacodynamic model parameters……………………………………………...11
Table S2. Unfavorable clinical/bacteriologic composite outcomes……………………………………..12
Table S3. Summary of clofazimine PK measures by Arm………………………………………….…13
Table S4. Summary of rifapentine PK measures by Arm……………………………………………..14
A5362 Trial Protocol………………………………………………………………………………15
A5362 Primary Statistical Analysis Plan………………………………………………………....136

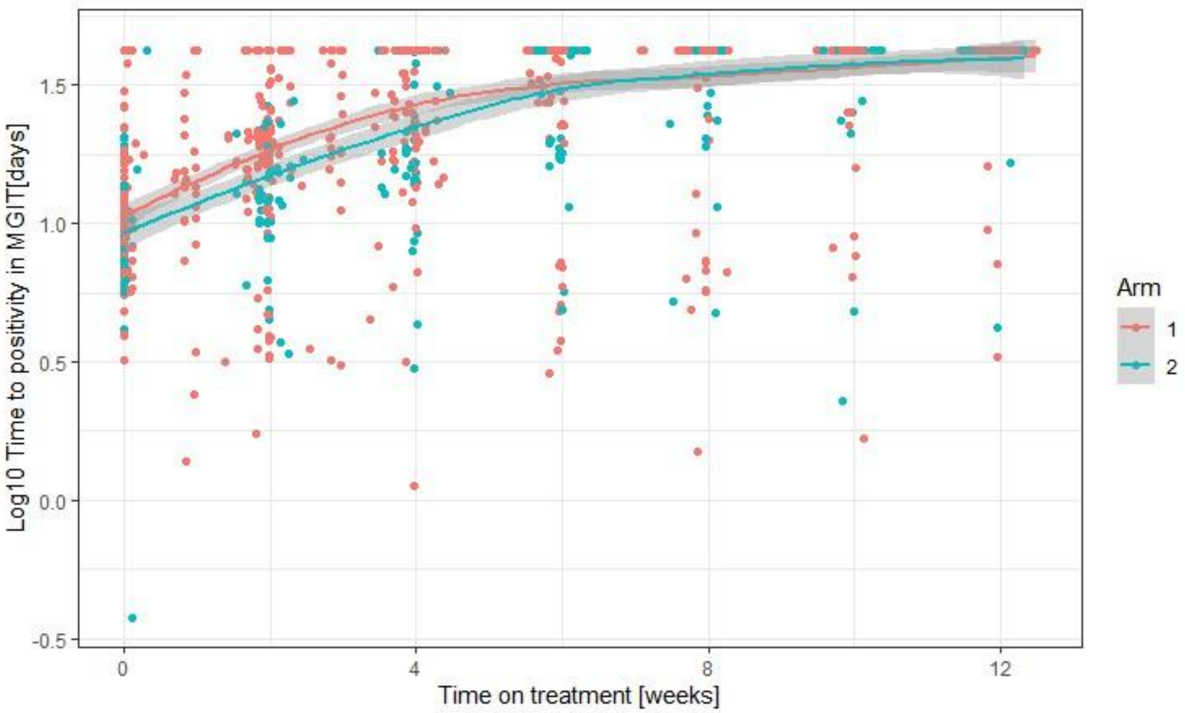

Figure S1. Time-to-positivity (TTP) data used for slope analysis, illustrated per arm. Negative samples included at censoring limit 42 days. Lines represent the loess-smooth per arm with 95% CIs.

Arm 1: 8 weeks of isoniazid, rifapentine, pyrazinamide, ethambutol, and clofazimine with a two-week loading dose, followed by 5 weeks of rifapentine, isoniazid, pyrazinamide, and clofazimine (13 weeks total treatment); Arm 2: HRZE=isoniazid, rifampin, pyrazinamide, and ethambutol.

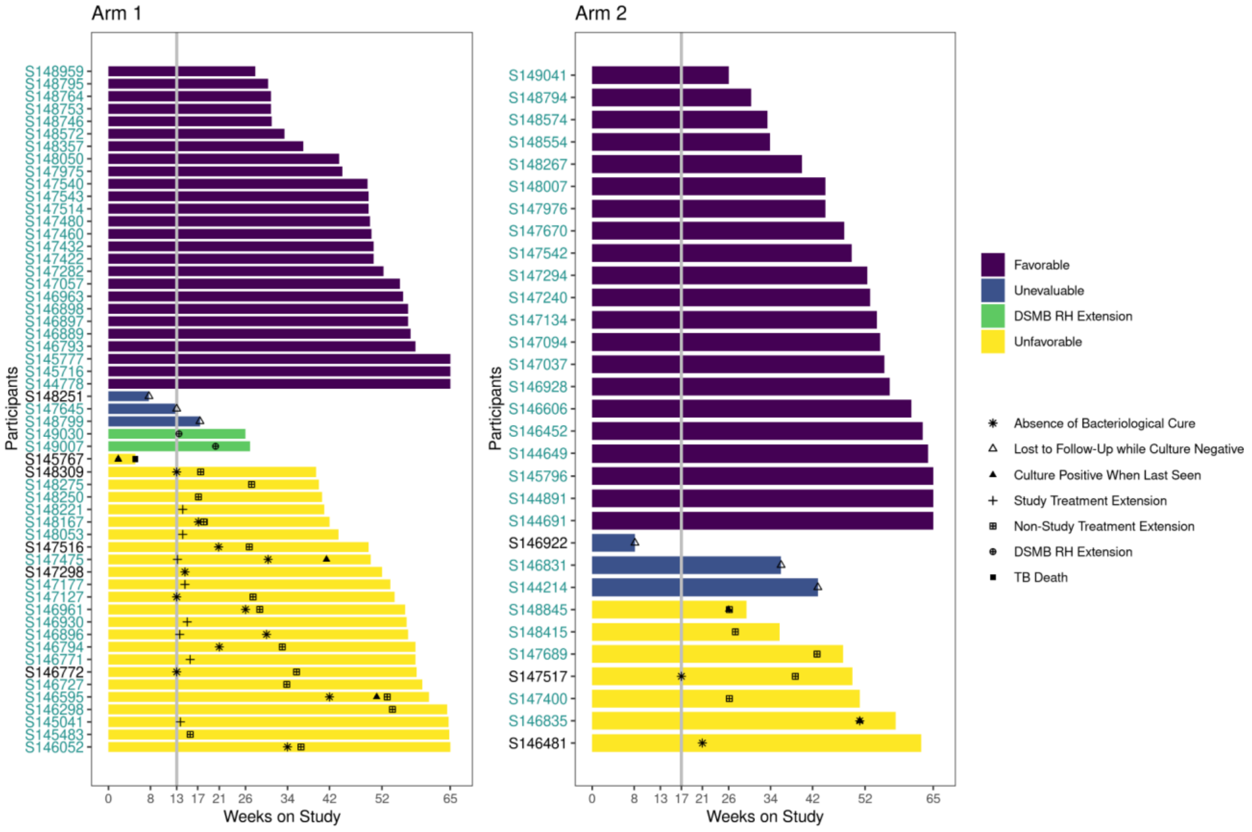

Figure S2. Time to unfavorable clinical/bacteriologic tuberculosis composite outcome through week 65. Patient identification numbers on the y-axis colored light green had sputum *M. tuberculosis* culture conversion by week 12 (i.e., favorable primary outcome). Study treatment extension refers to the continuation of respective study regimens beyond 14 days due to clinically inadequate response. Nonstudy treatment extension included referral to the domestic national TB program for standard of care treatment.

Arm 1: 8 weeks of isoniazid, rifapentine, pyrazinamide, ethambutol, and clofazimine with a two-week loading dose, followed by 5 weeks of rifapentine, isoniazid, pyrazinamide, and clofazimine (13 weeks total treatment); Arm 2: HRZE=isoniazid, rifampin, pyrazinamide, and ethambutol.

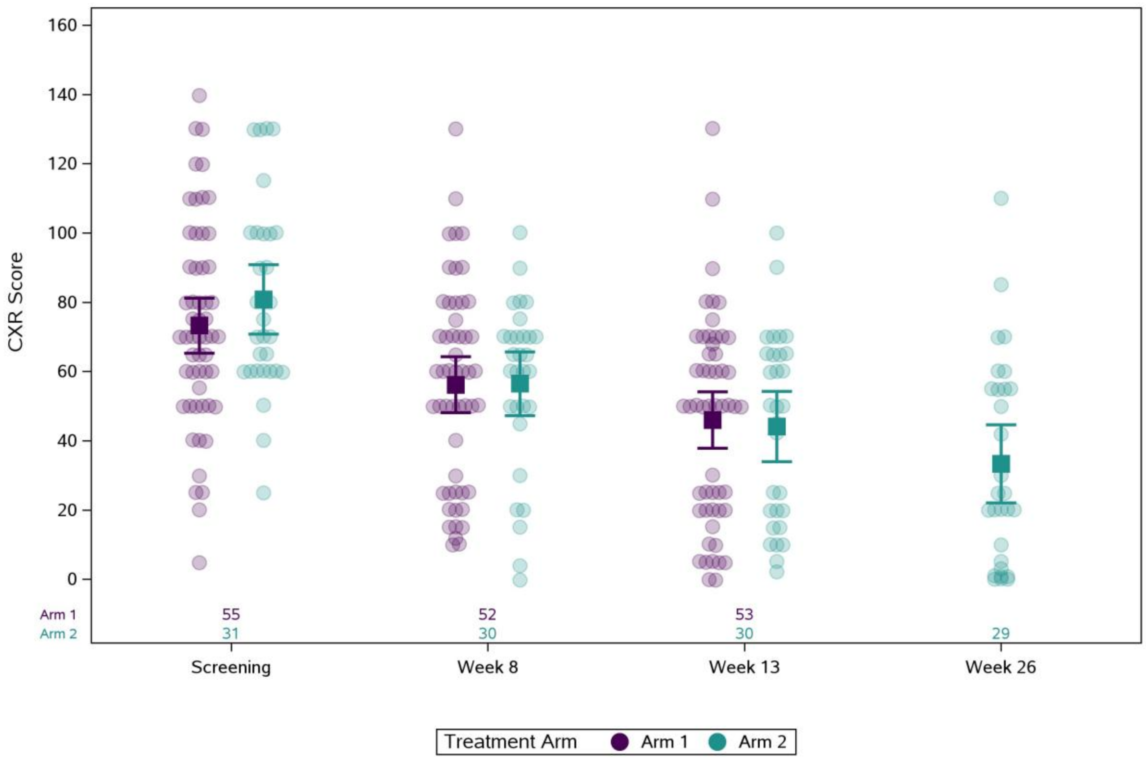

Figure S3. Mean Chest X-ray Score Over Time by Arm. Improvement in CXR score across the first 13 weeks of treatment did not significantly differ between groups but was significantly improved by end of respective treatment duration (i.e., week 13 in Arm 1, week 26 in Arm 2). Arm 1: 3HPZEC= 8 weeks of isoniazid, rifapentine, pyrazinamide, ethambutol, and clofazimine with a two-week loading dose, followed by 5 weeks of rifapentine, isoniazid, pyrazinamide, and clofazimine (13 weeks total treatment); Arm 2: HRZE=isoniazid, rifampin, pyrazinamide, and ethambutol.

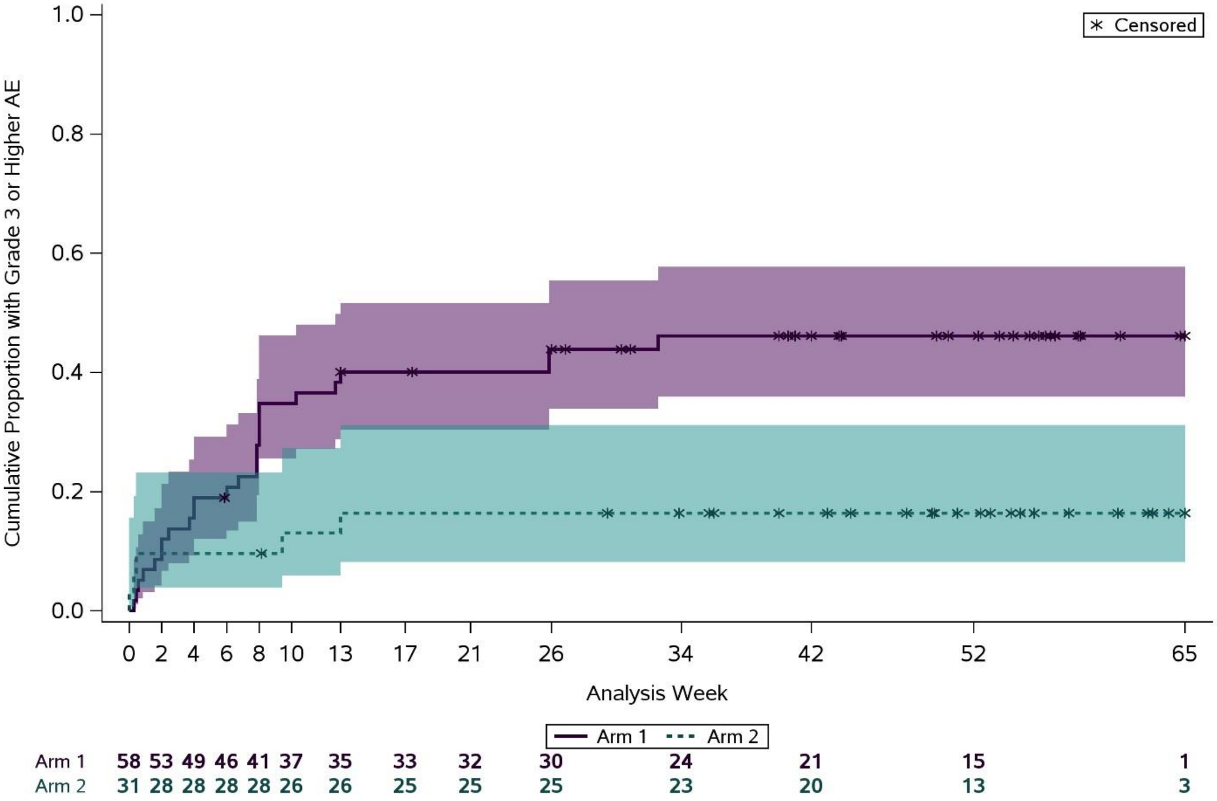

Figure S4. Time to first grade ≥3 adverse event through week 65. The line and shaded portion represent the Kaplan-Meier estimate and corresponding 95% confidence interval, respectively. Arm 1: 3HPZEC=8 weeks of isoniazid, rifapentine, pyrazinamide, ethambutol, and clofazimine with a two-week loading dose, followed by 5 weeks of rifapentine, isoniazid, pyrazinamide, and clofazimine (13 weeks total treatment); Arm 2: HRZE=isoniazid, rifampin, pyrazinamide, and ethambutol.

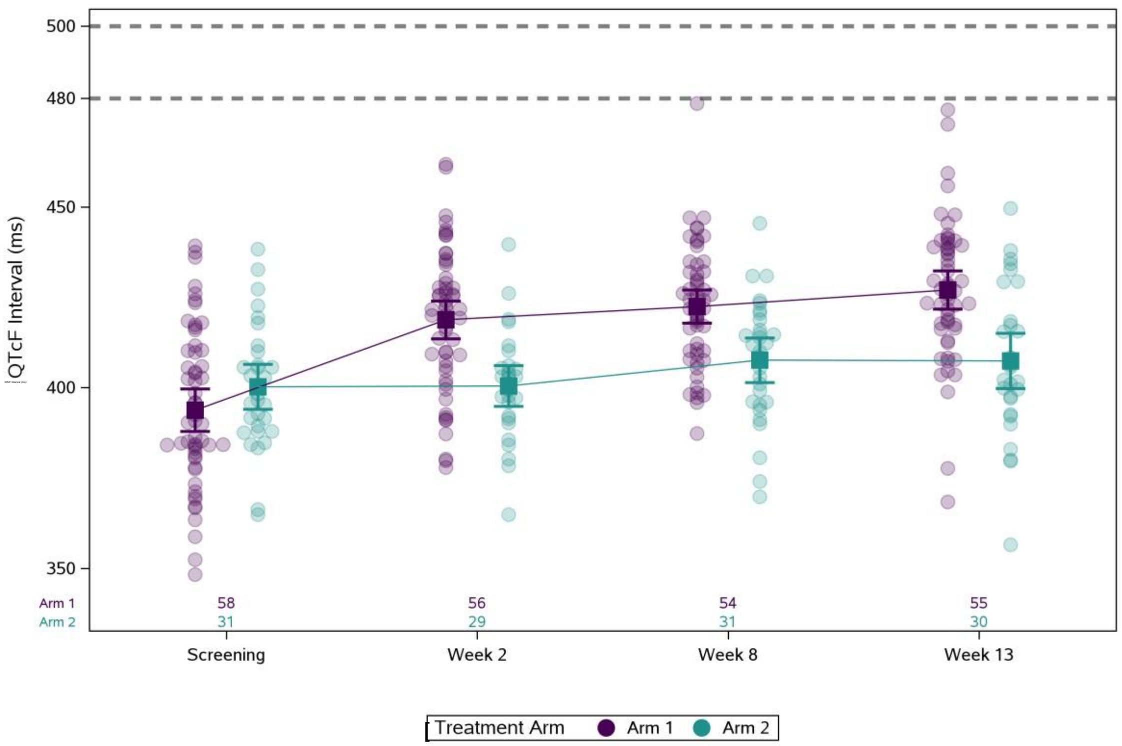

Figure S5. Mean QTcF interval change through 13 weeks. The circles represent participant-level QTcF interval measurements. The solid square for each arm and timepoint indicates the mean QTcF Interval (milliseconds) with bars representing the corresponding 95% Confidence Interval for the mean. Arm 1: 3HPZEC= 8 weeks of isoniazid, rifapentine, pyrazinamide, ethambutol, and clofazimine with a two-week loading dose, followed by 5 weeks of rifapentine, isoniazid, pyrazinamide, and clofazimine (13 weeks total treatment); Arm 2: HRZE=isoniazid, rifampin, pyrazinamide, and ethambutol; PK Arm 3= PHZEC once daily for 4 weeks, without a clofazimine loading dose, then HRZE according to local standard of care to complete 6 months of treatment.

S6a. Subjective change in skin coloration through 65 weeks.

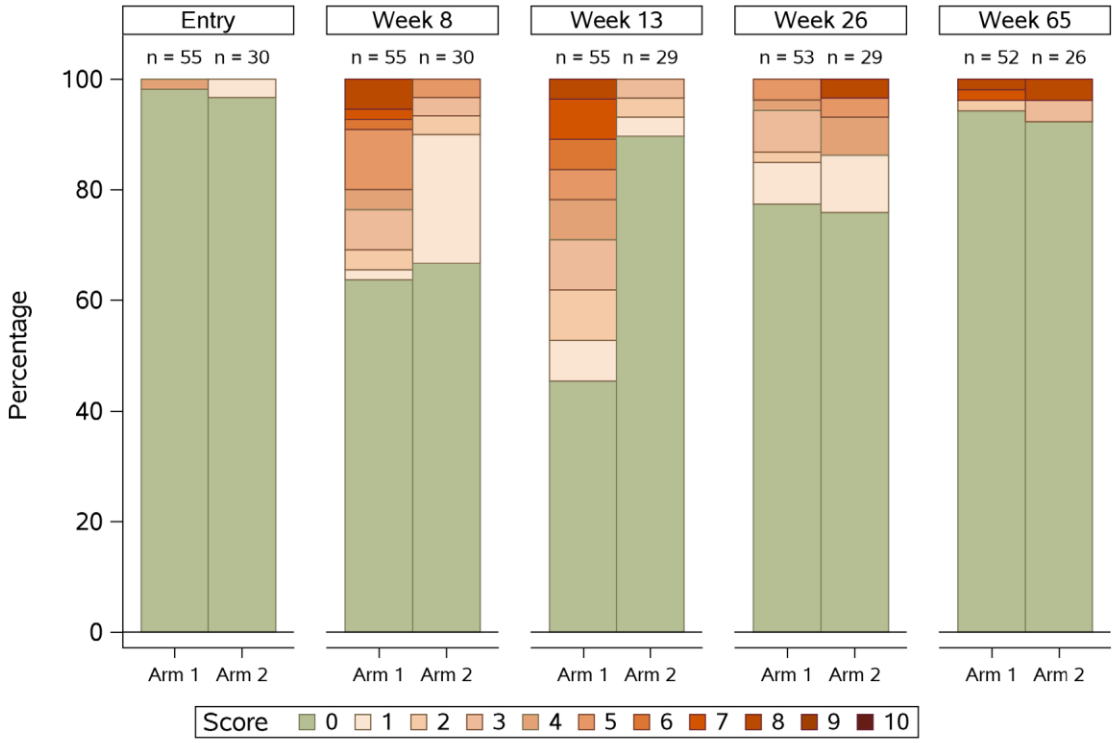

S6b. Subjective change in patient-reported distress due to skin coloration through 65 weeks.

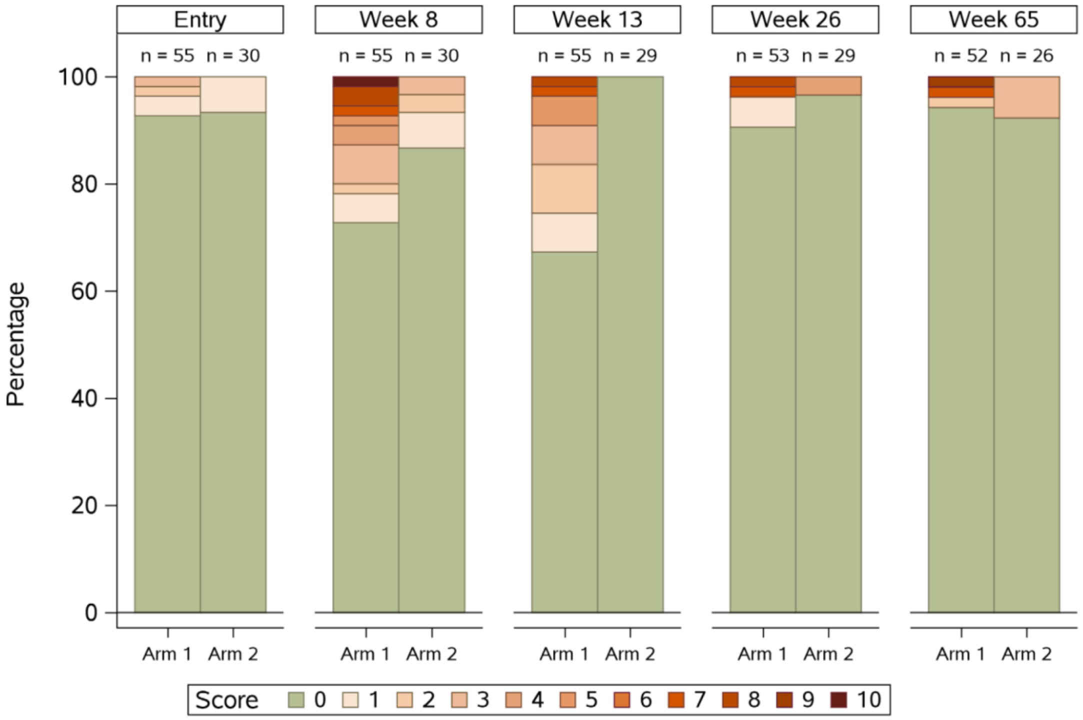

Figure S6. Subjective Change in Skin Coloration and Subjective Distress Caused by Change in Skin Coloration by Arm. Participant-reported changes in skin pigment (0 [none] to 10 [most significant possible]) and distress specifically related to skin pigment changes (0 [none] to 10 [worst possible]) were assessed by verbal response to a 10-point numeric rating scale.

Arm 1: 3HPZEC= 8 weeks of isoniazid, rifapentine, pyrazinamide, ethambutol, and clofazimine with a two-week loading dose, followed by 5 weeks of rifapentine, isoniazid, pyrazinamide, and clofazimine (13 weeks total treatment); Arm 2: HRZE=isoniazid, rifampin, pyrazinamide, and ethambutol.

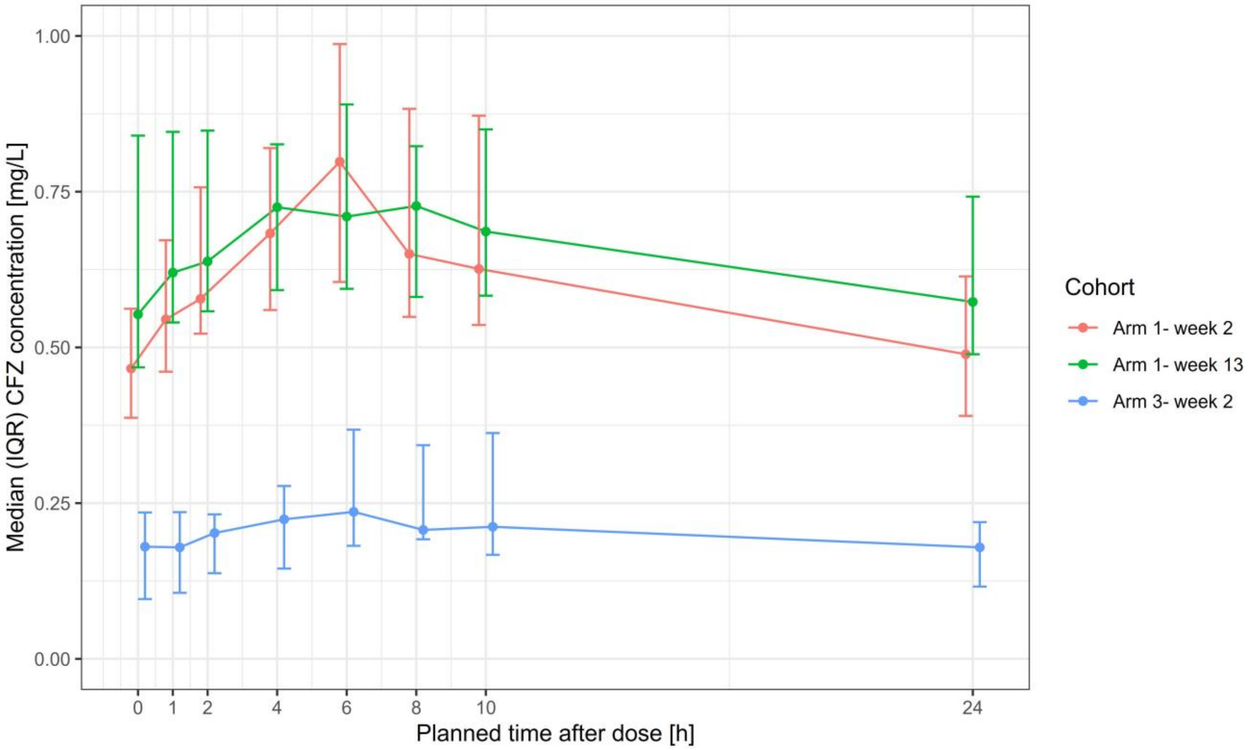

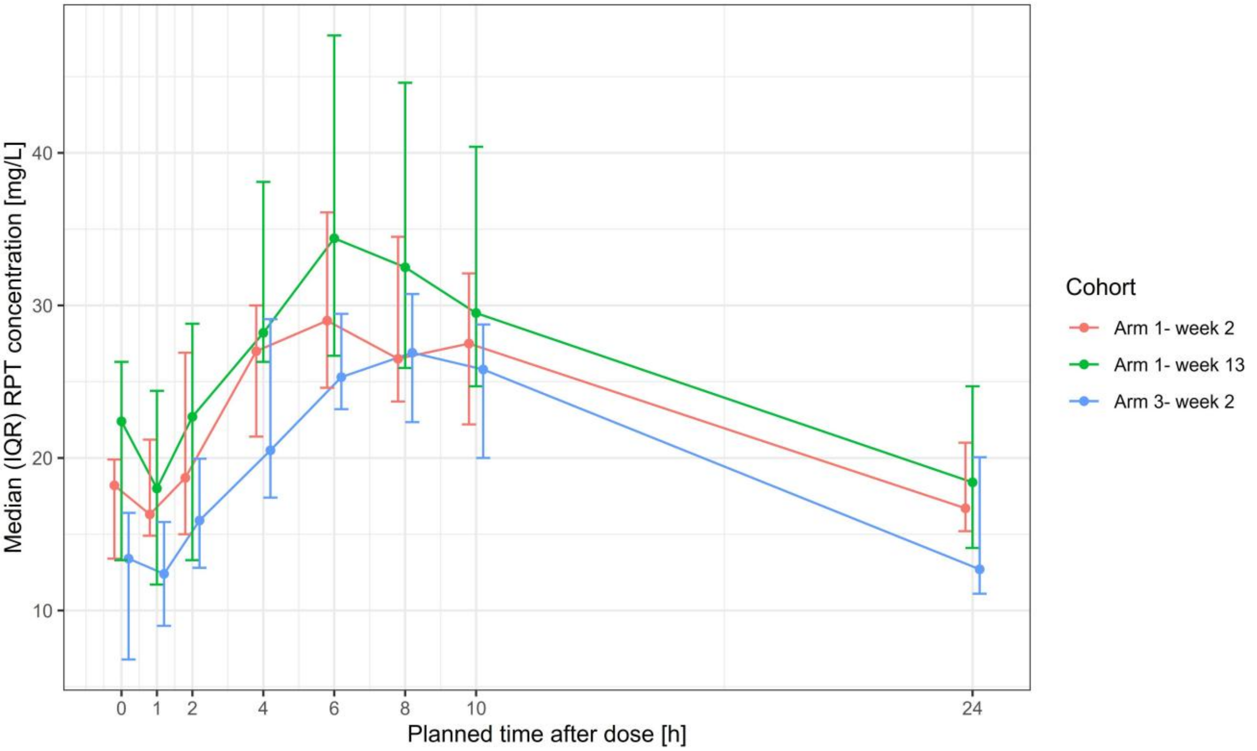

Figure S7. Median clofazimine (a) and rifapentine (b) concentration-time-curve over 24 hours after an observed dose. Error bars represent the interquartile range. Week 2 represents the completion of the loading-dose of clofazimine in Arm 1 (red) and Arm 3 (blue). Intensive PK sampling was repeated at week 13 in Arm 1 (green).

Arm 1: 3HPZEC= 8 weeks of isoniazid, rifapentine, pyrazinamide, ethambutol, and clofazimine with a two-week loading dose, followed by 5 weeks of rifapentine, isoniazid, pyrazinamide, and clofazimine (13

weeks total treatment); PK Arm 3= PHZEC once daily for 4 weeks, without a clofazimine loading dose, then HRZE according to local standard of care to complete 6 months of treatment.

Parameter	Best estimate (95% CI SIR)	RSE (%)
Fixed effects		
Baseline bacterial load (log10TTP [days])	0.978 (0.923 - 1.03)	2.74
**Slope Arm 1 (log10TTP*day^{-1})**	**0.0159 (0.0131 - 0.0201)**	**10.2**
**Slope Arm 2 (log10TTP*day^{-1})**	**0.0143 (0.0113 - 0.0186)**	**10.9**
Baseline negative culture chance [a]	0.08	
Random effects		
IIV baseline (CV%)	15.8 (9.96 - 21.5)	37.0
IIV slope Arm 1 (CV%)	65.0 (46.1 - 94.4)	26.2
IIV slope Arm 2 (CV%)	52.4 (33.4 - 90.2)	58.0
Residual error		
Additive error (log10TTP [days])	0.00998 (0.0074 - 0.0141)	14.1

[a] Determined as the proportion of culture negative TTP observations at baseline.
IIV= inter-individual variability. CV= coefficient of variation. RSE = Relative standard error. CI= confidence interval. TTP = Time to positivity.

Table S1. Final TPP pharmacodynamic model parameters. Arm 1: 3HPZEC= 8 weeks of isoniazid, rifapentine, pyrazinamide, ethambutol, and clofazimine with a two-week loading dose, followed by 5 weeks of rifapentine, isoniazid, pyrazinamide, and clofazimine (13 weeks total treatment); Arm 2: HRZE=isoniazid, rifampin, pyrazinamide, and ethambutol. To determine the TTP slopes, a linear mixed-effects model was fitted to log-10 transformed TTP data. Bi-linear models did not describe the data significantly better than linear models (degrees of freedom= 4, difference in objective function value = 2.9, p=0.57). The M3 method[33] was implemented to account for censored observations. Applying the upper limit of quantification at 25 days (suggested to have better properties for quantitative analysis)[34] resulted in better visual predictive checks than censoring at 42, hence the former was. A baseline negative culture chance (defined as the percentage of culture-negative samples at baseline after start of treatment) was included in the model to reflect the assay sensitivity.

	Arm 1 (n=55)	Arm 2 (n=31)
Participants with protocol-defined *M. tuberculosis*-related unfavorable outcome*	24	7
-- Microbiologically confirmed absence of cure†	12 (50%)	3 (43%)
-- Treatment extension due to clinically inadequate response	11 (46%)	4 (57%)
-- TB-related death‡	1 (4%)	0 (0%)

Table S2. Unfavorable clinical/bacteriologic composite outcomes. Data are n (% of those with an unfavorable outcome). Arm 1: 3HPZEC= 8 weeks of isoniazid, rifapentine, pyrazinamide, ethambutol, and clofazimine with a two-week loading dose, followed by 5 weeks of rifapentine, isoniazid, pyrazinamide, and clofazimine (13 weeks total treatment); Arm 2: HRZE=isoniazid, rifampin, pyrazinamide, and ethambutol.

As of September 25, 2023; categories are mutually exclusive. As of September 25, 2023; categories are mutually exclusive.

† Two or more positive cultures at or after week 13 (Arm 1) or week 17 (Arm 2); respectively, *n*=9 and *n*=1 occurred following culture conversion and end of treatment.

‡ This participant was *M. tuberculosis* culture positive.

	Arm 1	Arm 3
**Week 2**	(n=15)	(n=15)
Cmax (mg/L)	0.835 (0.737, 1.07)	0.236 (0.196, 0.393)
Tmax (h)	6.00 (4.01, 6.02)	6.15 (6.00, 8.00)
Cmin (mg/L)	0.450 (0.37, 0.56)	0.159 (0.0960, 0.218)
AUC (mg/L*h)	14.7 (13.3, 18.6)	4.98 (3.77, 7.38)
**Week 13**	(n=17)	
Cmax (mg/L)	0.756 (0.667, 0.890)	--
Tmax (h)	6.00 (6.00, 8.00)	--
Cmin (mg/L)	0.539 (0.458, 0.658)	--
AUC (mg/L*h)	15.7 (13.7, 18.7)	--

Table S3. Summary of clofazimine PK measures by Arm. All values are median (quartile 1, quartile 3). Arm 1: 3PHZEC=rifapentine, isoniazid, pyrazinamide, ethambutol, and clofazimine with a two-week loading dose of clofazimine 300 mg daily followed by clofazimine 100 mg daily, followed by 5 weeks of rifapentine, isoniazid, pyrazinamide, and clofazimine (13 weeks total treatment); Arm 3: 3PHZEC=rifapentine, isoniazid, pyrazinamide, ethambutol, and clofazimine 100mg daily without a loading dose for four weeks, followed by transition to standard of care therapy off study; AUC= area under the concentration time curve; Cmax = maximum concentration; Cmin = minimum concentration; Tmax; time to maximum concentration. The data from two participants in Arm 1 at week 2 were excluded from the summary metrics due to the intensive PK samples being taken after the first 100mg dose instead of the last 300mg dose.

	Arm 1	Arm 3
**Week 2**	(n=17)	(n=15)
Cmax (mg/L)	32.5 (27.2, 37.7)	26.9 (23.9, 32.1)
Tmax (h)	6.13 (6.00, 10.0)	8.00 (5.99, 8.04)
Cmin (mg/L)	14.9 (12.1, 19.9)	11.5 (6.46, 14.8)
AUC (mg/L*h)	549 (526, 662)	478 (405, 571)
**Week 13**	(n=17)	
Cmax (mg/L)	34.6 (28.8, 47.7)	
Tmax (h)	6.00 (4.17, 6.25)	
Cmin (mg/L)	16.9 (10.3, 21.8)	
AUC (mg/L*h)	612 (478, 863)	

Table S4. Summary of rifapentine PK measures by Arm. All values are median (quartile 1, quartile 3). Arm 1: 3PHZEC=rifapentine, isoniazid, pyrazinamide, ethambutol, and clofazimine with a two-week loading dose of clofazimine 300 mg daily followed by clofazimine 100 mg daily, followed by 5 weeks of rifapentine, isoniazid, pyrazinamide, and clofazimine (13 weeks total treatment); Arm 3: 3PHZEC=rifapentine, isoniazid, pyrazinamide, ethambutol, and clofazimine 100mg daily without a loading dose for four weeks, followed by transition to standard of care therapy off study; AUC= area under the concentration time curve; Cmax = maximum concentration; Cmin = minimum concentration; Tmax; time to maximum concentration.

A5362

A Phase IIc Trial of Clofazimine- and Rifapentine-Containing Treatment Shortening Regimens in Drug-Susceptible Tuberculosis: The CLO-FAST Study

A Multicenter Trial of the AIDS Clinical Trials Group (ACTG)

Sponsored by:

National Institute of Allergy and Infectious Diseases

IND # 147535

**The ACTG Tuberculosis Transformative Science Group Chair:**	**Kelly Dooley, MD, PhD**
**Protocol Chair:**	**John Metcalfe, MD, PhD, MPH**
**Protocol Co-Vice Chairs:**	**Samuel Pierre, MD**<br>**Kimberly Scarsi, PharmD, MS**
**DAIDS Clinical Representatives:**	**Richard Hafner, MD**<br>**Melanie Michele Goth, MD FAAP**
**Clinical Trials Specialist:**	**Austin Leah Van Grack, MPH**

FINAL Version 2.0
July 8, 2022

A Phase IIc Trial of Clofazimine- and Rifapentine-Containing Treatment Shortening Regimens in Drug-Susceptible Tuberculosis: The CLO-FAST Study

SIGNATURE PAGE

I will conduct the study in accordance with the provisions of this protocol and all applicable protocol-related documents. I agree to conduct this study in compliance with United States (US) Health and Human Service regulations (45 CFR 46); applicable US Food and Drug Administration regulations; standards of the International Conference on Harmonization Guideline for Good Clinical Practice (E6); Institutional Review Board/Ethics Committee determinations; all applicable in-country, state, and local laws and regulations; and other applicable requirements (e.g., US National Institutes of Health, Division of AIDS) and institutional policies.

Principal Investigator: ____
Print/Type

Signed: ____Date: _____________

TABLE OF CONTENTS

Page

SIGNATURE PAGE 2
SITES PARTICIPATING IN THE STUDY 5
PROTOCOL TEAM ROSTER 6
STUDY MANAGEMENT 10
GLOSSARY OF PROTOCOL-SPECIFIC TERMS 12
SCHEMA 14
1.0 HYPOTHESES AND STUDY OBJECTIVES 16
1.1 Hypotheses 16
1.2 Primary Objectives 16
1.3 Secondary Objectives 16
1.4 Exploratory Objectives 17
2.0 INTRODUCTION 18
2.1 Background 18
2.2 Clinical and Preclinical Evidence for CFZ Efficacy 18
2.3 Rationale 32
3.0 STUDY DESIGN 32
4.0 SELECTION AND ENROLLMENT OF PARTICIPANTS 33
4.1 Inclusion Criteria 33
4.2 Exclusion Criteria 36
4.3 Study Enrollment Procedures 37
4.4 Co-enrollment Guidelines 38
5.0 STUDY REGIMEN 39
5.1 Regimens, Administration and Duration 39
5.2 Formulations and Preparation 42
5.4 Concomitant Medications 42
6.0 CLINICAL AND LABORATORY EVALUATIONS 43
5.3 Pharmacy: Product Supply, Distribution, and Accountability 43
6.1 Schedule of Evaluations 43
6.2 Timing of Evaluations 49
6.3 Instructions for Evaluations 52
7.0 ADVERSE EVENTS AND STUDY MONITORING 61
7.1 Definition of Adverse Events 61
7.2 Adverse Event Collection Requirements for this Study 61
7.3 Expedited Adverse Event (EAE) Reporting to DAIDS 62
7.4 Study Monitoring 63
8.0 CLINICAL MANAGEMENT ISSUES 64
8.1 Toxicity 64

CONTENTS (Cont'd)

Page

8.2 Specific Conditions 65
8.3 Pregnancy 70

9.0 CRITERIA FOR DISCONTINUATION 70
9.1 Permanent and Premature Treatment Discontinuation 70
9.2 Premature Study Discontinuation 71

10.0 STATISTICAL CONSIDERATIONS 71
10.1 General Design Issues 71
10.2 Outcome Measures 71
10.3 Randomization and Stratification 76
10.4 Sample Size and Accrual 77
10.5 Data and Safety Event Monitoring 80
10.6 Analysis Sets 83
10.7 Analyses 83

11.0 PHARMACOLOGY PLAN 86
11.1 Pharmacology Objectives 86
11.2 Pharmacology Study Design 87
11.3 Primary and Secondary Data, Modeling and Data Analysis 88
11.4 Anticipated Outcomes 90

12.0 DATA COLLECTION AND MONITORING 90
12.1 Records to Be Kept 90
12.2 Role of Data Management 90
12.3 Clinical Site Monitoring and Record Availability 90

13.0 PARTICIPANTS 91
13.1 Institutional Review Board (IRB) Review and Informed Consent 91
13.2 Participant Confidentiality 91
13.3 Study Discontinuation 92

14.0 PUBLICATION OF RESEARCH FINDINGS 92

15.0 BIOHAZARD CONTAINMENT 92

16.0 REFERENCES 93

APPENDIX A: A5362 PROHIBITED AND PRECAUTIONARY MEDICATIONS 101

APPENDIX B: A5362 PERMITTED ANTIRETROVIRALS MEDICATIONS 103

APPENDIX I: SAMPLE INFORMED CONSENT 104

APPENDIX I-A: A5362 STUDY VISITS 117

APPENDIX I-B: SAMPLE CONSENT FORM FOR USE OF SAMPLES AND INFORMATION IN OTHER STUDIES 124

SITES PARTICIPATING IN THE STUDY

A5362 is open to non-US AIDS Clinical Trials Group (ACTG) clinical research sites (CRSs) in countries with reported cases of drug-susceptible tuberculosis (DS-TB). Refer to the Site tab on the protocol’s webpage on the ACTG Member website for the list of eligible sites.

PROTOCOL TEAM ROSTER

Chair
John Metcalfe, MD, PhD, MPH
University of California, San Francisco
Division of Pulmonary/Critical Care Med
San Francisco General Hospital
1001 Potrero Avenue, R**oo**m 5K1
San Francisco, CA 94110
Phone: 415-206-4679 or 415-596-9348
E-mail: john.metcalfe@ucsf.edu

Co-Vice Chairs
Samuel Pierre, MD
Les Centres Gheskio CRS
33 Boulevard Harry Truman
Port-au-Prince, HT-6110
HAITI
Phone: 509-3740-7711
E-mail: pierresamuel1@yahoo.fr

Kimberly Scarsi, PharmD, MS
University of Nebraska Medical Center
Department of Pharmacy Practice, Rm 3021
986145 Nebraska Medical Center
Omaha, NE 68198-6145
Phone: 402-559-9916
E-mail: kim.scarsi@unmc.edu

DAIDS Clinical Representatives
Richard Hafner, MD
Chief, TB Clinical Research Branch
DAIDS/NIAID/NIH/TBCB
5601 Fishers Lane, Room 9E30
Rockville, MD 20852
Phone: 301-435-3766
E-mail: rhafner@niaid.nih.gov

Melanie Michele Goth, MD, FAAP
TB Clinical Research Branch
TRP/DAIDS/NIAID
5601 Fishers Lane
Room 9E36
MSC 9829
Rockville, MD 20892-9829
Phone: 301-761-7490
E-mail: melanie.goth@nih.gov

Clinical Trials Specialist
Austin Leah Van Grack, MPH
ACTG Network Coordinating Center
Social & Scientific Systems, Inc.,
A DLH Holdings Company
8757 Georgia Avenue, 12th Floor
Silver Spring, MD 20910
Phone: 301-628-**3033**
E-mail: **austin.vangrack@dlhcorp.com**

Statisticians
Jorge Tomas Leon-Cruz
Statistical & Data Analysis Center
Harvard School of Public Health
FXB Building, Room 549
Boston, MA 02115
Phone: 617-432-7469
E-mail: jleoncru@sdac.harvard.edu

Isabelle R. Weir, PhD, MA
Statistical & Data Analysis Center
Harvard School of Public Health
651 Huntington Avenue
Boston, MA 02115
Phone: 617-432-2998
E-mail: iweir@sdac.harvard.edu

Data Manager**s**
Scott Anderson, MS Frontier Science & Technology Research Foundation, Inc.
4033 Maple Road
Amherst, NY 14226
Phone: 716-834-0900
E-mail: **sanders**@frontierscience.org

Jenna Meldrum, BA
Frontier Science Foundation
4033 Maple Road
Amherst, NY 14226
Phone: 716-834-0900
E-mail: meldrum@frontierscience.org
Data Managers (Cont'd)

PROTOCOL TEAM ROSTER (Cont'd)

Brooke Altman
Frontier Science Foundation
4033 Maple Road
Amherst, NY 14226
Phone: 716-834-0900
E-mail: altman@frontierscience.org

DAIDS Pharmacist
Justine Beck, PharmD, BCPS, RPh
Pharmaceutical Affairs Branch
OCSO/DAIDS/NIAID/NIH/DHHS
5601 Fishers Lane, 9D39, MSC 9829
Rockville, MD 20852
Phone: 301-761-5288
E-mail: justine.beck@nih.gov

Pharmacologist
Gary Maartens, MBChB, MMed
University of Cape Town
Division of Clinical Pharmacology
Anzio Road, Cape Town 7925
SOUTH AFRICA
Phone: 27-21-4066286
E-mail: gary.maartens@uct.ac.za

Pharmacometrician
Elin Svensson, PhD
Radboud University Medical Center
Department of Pharmacy
P.O. Box 9101, 62 HB Nijmegen THE
NETHERLANDS
Phone: 31-623217888
E-mail: elin.svensson@radboudumc.nl

Dermatologist
Maria Wei, MD, PhD
Department of Dermatology
VA 190, 4150 Clement Street
San Francisco, CA 94121
E-mail: maria.wei@ucsf.edu

Investigators
Richard E. Chaisson, MD
Division of Infectious Diseases
Johns Hopkins University
1503 E. Jefferson Street

Baltimore, MD 21231
Phone: 410-955-1755
E-mail: rchaiss@jhmi.edu

Jennifer Furin, MD, PhD
Case CRS,
2120 Circle Drive, Room E-202
Cleveland, OH 44106
Phone: 216-368-6727
E-mail: jjf38@case.edu

Jacques Grosset, MD
Johns Hopkins University CRS
CRB2, Room 105
1550 Orleans Street
Baltimore, MD 21231-1044
Phone: 410-502-8234
E-mail: jgrosse4@gmail.com

David W. Haas, MD
Vanderbilt Therapeutics CRS
One Hundred Oaks
719 Thompson Lane, Suite 47183
Nashville, TN 37204
Phone: 615-936-8594
E-mail: david.haas@vanderbilt.edu

Natthapol Kosashunhanan, MD
Chiang Mai University HIV Treatment CRS
Chiang Mai University Research Institute for Health Sciences
110 Intavaroros Road
Chiang Mai 50200
THAILAND
Phone: 66-53-3945055 Ext. 354
E-mail: natapolk@yahoo.com

PROTOCOL TEAM ROSTER (Cont'd)

Investigators (Cont'd)

Rahul Lokande, MD
Byramjee Jeejeebhoy Medical College (BJMC)
CRS (31441)
Pulmonary Medicine Department OPD
NO. 34, Sassoon General Hospital
Jai Prakash Narayan Road
Pune 411001
INDIA
Phone: 91-98-22894972
E-mail: drrahul_2007@yahoo.com

Alberto Mendoza-Ticona MD,
Socios en Salud Sucursal Peru CRS
(31985) Jr. Puno 279, Cercado de Lima
Lima 15001
PERU
Phone: 51 953982909
E-mail: **amendoza_ses@pih.org**

Gus Rosania, PhD
Department of Pharmaceutical Sciences
University of Michigan College of Pharmacy
428 Church Street
Ann Arbor, MI 48109
Phone: 734-358-5661
E-mail: grosania@med.umich.edu

Wadzanai Samaneka MBChB, MSc
Parirenyatwa CRS, P.O. Box A5178
Corner Josiah Tongogara and Mazowe
Harare 263
ZIMBABWE
Phone: 263-242-705986
E-mail: wsamaneka@uzchs-ctrc.org

International Program Specialist

Akbar Shahkolahi, PhD
ACTG Network Coordinating Center
Social and Scientific Systems, **Inc.,**
A DLH Holdings Company
8757 Georgia Avenue, 12th Floor
Silver Spring, MD 20910
Phone: 301-628-3318
E-mail: akbar.shahkolahi@dlhcorp.com

Field Representative

Joan Coetzee, CPN, PN
Tygerberg Hospital
Ward J8/ Francie van Zijl Drive
Parow Valley, 7505
Cape Town 7505
SOUTH AFRICA
Phone: 27-21-9384157
E-mail: joan@sun.ac.za

Community Scientific Subcommittee (CSS) Representative

Anne Wambui Njoroge
Moi University Clinical Research Center (MUCRC)
CRS (12601)
Chandaria Cancer and Chronic Diseases
Centre at Moi Teaching and Referral Hospital
Nandi Road, P.O. Box: 4606
Eldoret 30100
KENYA
Phone: 254-728934777
E-mail: anbartilol@yahoo.com

Laboratory Data Managers

Brooke Altman
Frontier Science & Technology Research Foundation
4033 Maple Road
Amherst, NY 14226
Phone: 716-834-0900
E-mail: altman@frontierscience.org

Laura Hovind, BS, MS
Frontier Science & Technology Research
Foundation
4033 Maple Road
Amherst, NY 14226
Phone: 716-834-0900 x7468
E-mail: hovind@fstrf.org

Kevin Knowles
Frontier Science & Technology Research
Foundation
4033 Maple Road
Amherst, NY 14226
Phone: 716-834-0900 x7238

E-mail: knowles@frontierscience.org

PROTOCOL TEAM ROSTER (Cont'd)

Laboratory Technologists
Brian Clagett, BA
Case CRS
BRB, Room 1048B
2109 Adelbert Road
Cleveland, OH 44106
Phone: 216-368-4853
E-mail: bmc@case.edu

Neeta N. Pradhan, MSC
Byramjee Jeejeebhoy Government
Medical College CRS (31441)
Jaiprakash Narayan Road
P.O. Box 411001
Pune 411001
INDIA
Phone: 985-0044897
E-mail: neetapradhanpune@gmail.com

Laboratory Specialists
Afton Dorasamy, BS
ACTG Laboratory Center
University of California Los Angeles
11075 Santa Monica Blvd. Suite #200
Los Angeles, CA 90025
Phone : 443-972-0685
E-mail : adorasamy@milabcentral.org

Cherisse Heirs, BA
ACTG Laboratory Center
University of California Los Angeles
11075 Santa Monica Blvd. Suite #200
Los Angeles, CA 90025
Phone: **585-397-4730**
Email: **cheirs@milabcentral.org**

Source Document Specialist
Josie Marshall, BS
ACTG Network Coordinating Center
Social and Scientific Systems, Inc.,
A DLH Holdings Company
8757 Georgia Avenue, 12th Floor
Silver Spring, MD 20910

Phone: 303-724-0803
E-mail: josie.marshall@dlhcorp.com

STUDY MANAGEMENT

All general questions concerning this protocol should be sent to actg.teama5362@fstrf.org via e-mail. The appropriate team member will respond with a "cc" to actg.teama5362@fstrf.org. A response should generally be received within 24 hours (Monday-Friday).

Protocol E-mail Group
Sites should contact the User Support Group at the Data Management Center (DMC) as soon as possible to have the relevant personnel at the site added to the actg.prota5362 e-mail group. Include the protocol number in the email subject line.

- Send an e-mail message to actg.user.support@fstrf.org.

Clinical Management:
For questions concerning entry criteria, toxicity management, concomitant medications, coenrollment, and a**ll decisions regarding change, restart, or extension of TB treatment**, contact the Clinical Management Committee (CMC).

- Send an e-mail message to actg.cmcA5362@fstrf.org. Include the protocol number, patient identification number (PID), and a brief relevant history.

Laboratory
For questions specifically related to pharmacology laboratory tests, contact the protocol pharmacologist.

- Send an e-mail message to actg.teama5362@fstrf.org (ATTENTION: Gary Maartens**/Elin Svensson**).

Data Management
For nonclinical questions about transfers, inclusion/exclusion criteria, electronic case report forms (eCRFs), randomization/registration, and other data management issues, contact the data manager. Completion guidelines for eCRFs and participant-completed eCRFs can be downloaded from the FSTRF website at www.frontierscience.org.

- For transfers, reference the Study Participant Transfer SOP 119, and contact **Brooke Altman, Scott Anderson, and Jenna Meldrum** directly.
- For other questions, send an e-mail message to actg.teama5362@fstrf.org (ATTENTION: **Brooke Altman/Scott Anderson/Jenna Meldrum**).
- Include the protocol number, PID, and a detailed question.

Randomization/Participant Registration
For randomization/participant registration questions or problems and study identification number (SID) lists:

- Send an e-mail message to rando.support@fstrf.org. Call the Statistical and Data Analysis Center (SDAC)/DMC Randomization Desk at 716-834-0900 extension 7301.

DMC Portal and Medidata Rave Problems Contact
DMC User Support:

- Send an e-mail message to actg.user.support@fstrf.org or call 716-834-0900 x7302.

Protocol Document Questions
For questions concerning the protocol document, contact the Clinical Trials Specialist.

- Send an e-mail to actg.teama5362@fstrf.org (ATTENTION: **Austin Van Grack**).

Copies of the Protocol
To request a hard copy of the protocol, send an e-mail message to ACTGNCC@**dlhcorp**.com. Electronic copies can be downloaded from the ACTG website (https://www.actgnetwork.org).

Product Package Inserts and/or Investigator Brochures
To request copies of product package inserts or investigator brochures, contact the DAIDS Regulatory Support Center (RSC) at RIC@tech-res.com or call 301-897-1708.

Protocol Registration
For protocol registration questions, send an e-mail message to Protocol@tech-res.com or call 301-897-1707.

Protocol Activation
For questions related to protocol activation at non-US sites contact the ACTG Site Coordination Group.

- Send an email message to actgsitecoordination@**dlhcorp**.com.

Study Product
For questions or problems regarding study product, dose, supplies, records, and returns, call Justine Beck, protocol pharmacist, at 301-761-5288.

Study Drug Orders
Call the Clinical Research Products Management Center (CRPMC) at 301-294-0741.

IND (Investigational New Drug) Number or Questions
The IND number will be available on the PSWP within 30 days of the submission to the FDA. For any questions related to the IND submission, contact the DAIDS RSC at Regulatory@techres.com or call 301-897-1706.

Expedited Adverse Event (EAE) Reporting/Questions
Contact DAIDS through the RSC Safety Office at DAIDSRSCSafetyOffice@tech-res.com or call 1-800-537-9979 or 301-897-1709; or fax 1-800-275-7619 or 301-897-1710.

Telephone Calls
Sites are responsible for documenting any phone calls made to A5362 team members.

- Send an e-mail to actg.teama5362@fstrf.org.

Protocol-Specific Web Page
Additional information about management of the protocol can be found on the protocol-specific web page (PSWP).

GLOSSARY OF PROTOCOL-SPECIFIC TERMS

AE	adverse event
AMEZ	amikacin/moxifloxacin/ethambutol/pyrazinamide
AMEZC	amikacin/moxifloxacin/ethambutol/pyrazinamide/clofazimine

ART	antiretroviral therapy
ATP	adenosine triphosphate
AUC	area under the concentration-time curve
BDQ	bedaquiline
CES-D	Center for Epidemiologic Studies Depression Scale
CFU	colony-forming unit
CFZ (or C)	clofazimine
CI	confidence interval
C_{max}	maximum concentration
CYP	cytochrome P450
DOT	directly observed therapy
DS-TB	drug susceptible tuberculosis
DST	drug susceptibility testing
DTG	dolutegravir
EAE	expedited adverse event
EBA	early bactericidal activity
ECG	electrocardiogram
EFV	efavirenz
EMB (or E)	ethambutol
EOT	end of treatment
FDA	Food and Drug Administration
HR	hazard ratio
INH (or H)	isoniazid
ITT	intent-to-treat
LPA	line probe assay (Hain GenoType MTBDRplus v2.0 or greater)
MDR-TB	multidrug-resistant tuberculosis
MGIT	mycobacteria growth indicator tube
MIC	minimum inhibitory concentration
mITT	modified intent-to-treat
MOPS	Manual of Procedures
ms	milliseconds

GLOSSARY (Cont'd)

Mtb	*Mycobacterium tuberculosis*
NTM	nontuberculous mycobacteria
NTP	National Tuberculosis Program
PD	pharmacodynamic
PHZ	rifapentine/isoniazid/pyrazinamide
PHZE	rifapentine/isoniazid/pyrazinamide/ethambutol
PK	pharmacokinetics
PZA (or Z)	pyrazinamide
QTcF	corrected QT based on the Fridericia correction method
RAL	raltegravir
RH	rifampicin/isoniazid
RHZE	rifampicin/isoniazid/pyrazinamide/ethambutol
RIF (or R)	rifampicin (also referred to as rifampin)
RPT (or P)	rifapentine
**RS ratio**	**ribosomal RNA synthesis ratio**
SAE	serious adverse event
SOC	standard of care
TB	tuberculosis
WHOQOL-BREF	World Health Organization Quality of Life-BREF
XDR-TB	extensively drug-resistant tuberculosis
Xpert	nucleic acid amplification diagnostic test to identify MTB DNA and resistance to rifampicin

SCHEMA

A Phase IIc Trial of Clofazimine- and Rifapentine-Containing Treatment Shortening Regimens in Drug-Susceptible Tuberculosis: The CLO-FAST Study

DESIGN
A5362 is a Phase IIc, randomized, open-label comparison of a 3-month rifapentine (RPT)/clofazimine (CFZ)-containing regimen with CFZ loading dose (Arm 1, experimental) versus 6-month standard of care (SOC; Arm 2) for drug-susceptible (DS) tuberculosis (TB). Early bacteriologic efficacy, early relapse, safety, and tolerability of the 3-month experimental arm versus standard of care will be evaluated. A 65-week composite efficacy outcome including treatment failure, relapse, and death will be a key secondary outcome. A PK-only subgroup to study the effect of the CFZ loading dose will be concurrently randomized to a RPT/CFZcontaining regimen without CFZ loading dose (Arm C, PK only subgroup) but will not be included in efficacy analyses.

DURATION
65 weeks

SAMPLE SIZE
The total sample size will be 185.
Arm 1 (Experimental): 110
Arm 2 (Standard of Care): 55
Arm C (PK only subgroup): 20

POPULATION
Participants aged ≥18 years living with and without HIV co-infection, and with pulmonary TB without demonstrated isoniazid or rifampicin resistance. Participants living with HIV with CD4+ T-cell count ≥100 cells/mm^3 are eligible to enroll, and co-administration of antiretroviral therapy is required.

STRATIFICATION
Participants will be stratified based on HIV status, and presence of advanced disease based on chest X-ray conducted at screening.

REGIMEN
Participants will be randomized to Arm 1, 2, or C:

Arm 1 (Experimental 3-month, with CFZ loading dose): rifapentine/isoniazid/pyrazinamide/ethambutol (PHZE) + clofazimine (CFZ) 300 mg once daily for 2 weeks; then PHZE + CFZ 100 mg once daily for 6 weeks; then rifapentine/isoniazid/pyrazinamide (PHZ) + CFZ 100 mg once daily for 5 weeks, for a total of 13 weeks of study treatment.

Arm 2 (Standard of Care): rifampicin/isoniazid/pyrazinamide/ethambutol (RHZE) for 8 weeks; then rifampicin/isoniazid (RH) for 18 weeks, for a total of 26 weeks of study treatment.

SCHEMA (Cont'd)

Arm C (PK only subgroup): PHZE + CFZ 100 mg for 4 weeks; then remain on study, off study medications and be treated per SOC (completing RHZE for 4 weeks; then RH for 18 weeks).

CFZ and RPT are investigational agents. Rifampicin (RIF or R), isoniazid (INH or H), pyrazinamide (PZA or Z), and ethambutol (EMB or E) are study-supplied standard TB drugs.

Schema Figure 1

	Weeks					
	0-2	3-4	5-8	9-13	14-26	27-65
Arm 1 (Experimental) N=110	PHZE + CFZ 300 mg	PHZE + CFZ 100 mg	PHZE + CFZ 100 mg	PHZ + CFZ 100 mg	Follow-up	Follow-up
Arm 2 (Standard of Care) N=55	RHZE			RH		Follow-up
Arm C (PK only subgroup) N=20	PHZE + CFZ 100 mg	PHZE + CFZ 100 mg	RHZE: On SOC treatment and study follow-up	RH: On SOC treatment and study follow-up		Follow-up

1.0 HYPOTHESES AND STUDY OBJECTIVES

1.1 Hypotheses

Primary Hypothesis

A 3-month regimen of rifapentine/isoniazid/pyrazinamide/ethambutol (PHZE) with clofazimine (CFZ) dosed as 100 mg daily with a 2-week 300 mg daily loading dose (Arm 1) will demonstrate early efficacy (time to 12-week liquid culture conversion) relative to standard of care (SOC) (RHZE; Arm 2), and will have acceptable safety and tolerability.

1.2 Primary Objectives

1.2.1 To compare time to 12-week liquid culture conversion between Arm 1 and Arm 2 participants

1.2.2 To compare adverse events (safety) over 65 weeks **between Arm 1 and Arm 2 participants**

1.3 Secondary Objectives

1.3.1 To compare proportion of participants who experience **a** favorable composite efficacy outcome at 65 weeks **between Arm 1 and Arm 2 participants** (see Table 10.4-1 and section 10.2.4**B**)

1.3.2 To compare premature regimen discontinuation (tolerability) **between Arm 1 and Arm 2 participants**

1.3.3 To estimate and compare CFZ-associated increases in QT interval (QTcF, using the Fridericia correction)

1.3.4 To estimate and compare time to stable liquid and solid mycobacterial culture conversion

1.3.5 To compare proportion of participants with liquid and solid culture conversion at weeks 8 and 12 **between Arm 1 and Arm 2**

1.3.6 To compare days to positivity in liquid culture over treatment time

1.3.7 To compare chest radiographic score (with specific attention to resolution of cavitary disease) from baseline to end of treatment (Arm 1, week 13; **Arm 2**, week 26) **between Arm 1 and Arm 2 participants**

1.3.8 To compare proportion of participants who experience a favorable clinical/bacteriologic treatment outcome at 65 weeks **between Arm 1 and Arm 2 participants** (see section 10.2.4**A**)

1.3.9 To estimate cumulative relapse and recurrence proportions up to week 65 **for participants on Arm 1 and Arm 2**

1.3.10 To determine CFZ pharmacokinetic (PK) parameters in plasma in Arm 1

1.3.11 To compare PK parameters when CFZ is given with a loading dose in Arm 1 and without a loading dose in Arm C

1.3.12 To objectively and subjectively assess CFZ-associated skin hyperpigmentation **between Arm 1 and Arm 2 participants**

1.4 Exploratory Objectives

1.4.1 To examine the association of plasma concentrations of CFZ with:

1.4.1.1 QTcF changes from baseline, on treatment and post-treatment, using a population pharmacokinetic-pharmacodynamic (PK-PD) model

1.4.1.2 Time to stable (liquid and solid) culture conversion

1.4.1.3 Time (days) to positivity in liquid culture (MGIT) over time after start of treatment

1.4.1.4 CFZ-associated adverse events

1.4.1.5 Emergence of CFZ drug resistance (and cross-resistance with bedaquiline) as determined by phenotypic and genotypic methods

1.4.2 To describe RPT PK parameters when combined with CFZ

1.4.3 To explore whether efficacy, safety and/or PK of CFZ are associated with polymorphisms in human genes that may affect metabolism, disposition, and toxicity of study drugs as well as concomitant medications (e.g., *NAT2* and *CYP2B6*)

1.4.4 To assess social stigma and the impact of perceived skin changes on quality of life in a subset of **Arm 1 and Arm 2** participants using an open-ended qualitative interview

1.4.5 To estimate and compare changes in quality of life at weeks 8, 13, 26, and 65

1.4.6 To estimate and compare changes in symptoms of depression at weeks 8, 13, 26, and 65

1.4.7 To evaluate the sputum ribosomal synthesis (RS) ratio, an exploratory molecular pharmacodynamic (PD) marker, as follows:

1.4.7.1 Compare the sputum RS ratio between Arm 1 and Arm 2 (weeks 2 and 4) and between the intensive PK groups in Arm 1 and Arm C (weeks 1, 2, 3, and 4)

1.4.7.2 Examine association of the sputum RS ratio at weeks 2 and 4 with time to 12-week liquid culture conversion

1.4.7.3 Examine association of the sputum RS ratio at weeks 2 and 4 with 65-week composite efficacy outcome

1.4.7.4 Characterize the sputum RS ratio at the time of Possible Poor Treatment Response (as defined in section 6.2.3)

1.4.7.5 Determine the longitudinal association of the sputum RS ratio with clinical, radiographic, and PK measures

2.0 INTRODUCTION

2.1 Background

CFZ is a fat-soluble riminophenazine dye first synthesized in 1954 by Barry et al. as an anti-TB drug [1-3]. CFZ became an established antileprosy drug following demonstration of efficacy against *Mycobacterium leprae* in 1955 [4]. Since the 1990s, the growing global burden of drug resistant TB rekindled interest in CFZ as a key repurposed constituent of newer TB regimens.

2.2 Clinical and Preclinical Evidence for CFZ Efficacy

In 2010, an unblinded, single-site, sequential observational cohort study of the Bangladesh regimen, a cocktail of seven agents given for 4 months followed by four drugs for 5 months, with CFZ throughout, demonstrated a 15% increase in cure (with no increase in patient default) in treatment of fluoroquinolone-sensitive multi-drug resistant TB (MDR-TB) [5]. A subsequent follow-up study of over 500 patients treated with the Bangladesh regimen demonstrated 84.5% bacteriologically favorable treatment outcomes, with >80% of patients completing 24month post-treatment follow-up [6]. The Bangladesh regimen was later evaluated in the study, “The Evaluation of a Standardized Treatment Regimen of Anti-tuberculosis Drugs for Patients with Multi-drug Resistant Tuberculosis” (STREAM I, with STREAM II ongoing) [7]. STREAM I demonstrated noninferior efficacy (long-term successful outcome in more than 78% of participants) and similar safety relative to conventional 20-month regimens, though more deaths were reported in the short-regimen group (8.5% vs. 6.4%) [8]. A randomized trial evaluating CFZ (100 mg daily for 21 months versus placebo) within an optimized MDR-TB background regimen found significantly faster time to culture conversion and healing of cavities on chest computed tomography, and higher treatment success (73.6% vs. 53.8%; p=0.035) in patients randomized to CFZ; relapse

was not assessed [9]. A second small randomized clinical trial evaluating CFZ as part of individualized XDR-TB regimens over 36 months found no difference in time to sputum culture conversion on solid medium [10]. Of note, no patient discontinued therapy because of adverse events in the CFZ arm of either trial. Finally, additional observational clinical data in MDR-TB and extensively drug-resistant TB (XDR-TB) from multiple settings are supportive of CFZ efficacy [11-15].

While a recent human early bactericidal activity (EBA) study showed no antimicrobial effect of CFZ during 14 days of therapy [16], CFZ has demonstrated significant *Mycobacterium tuberculosis* (*Mtb*) killing activity and treatment-shortening potential in murine models. In a study of second-line anti-TB drugs [17], the addition of CFZ 25mg/kg/day (dose approximating 200 mg/day in humans) to a 2-month regimen of amikacin/moxifloxacin/ethambutol/pyrazinamide (AMEZ) followed by 7 months of AMEZ alone dramatically increased clearance of isoniazid (INH)-resistant *Mtb* (H37Rv) from the lungs of BALB/c mice, with most being culture-negative after 5 months of treatment, compared with none at 9 months of AMEZ alone (average log_{10}CFU of 4). Relapse rates between the two groups were unable to be compared, as mice treated with the CFZ-free regimen were still heavily culture positive at treatment cessation. Among mice treated with AMEZC, relapses occurred similarly with treatment durations of 5 months (3/15) and 9 months (1/15), suggesting that a plateau in sterilization capability of CFZ had been reached long before treatment cessation [17].

These findings led to a subsequent mouse model of drug-susceptible TB (DS-TB) demonstrating that substitution of CFZ (25 mg/kg/day) for ethambutol (EMB) in the standard DS-TB regimen of isoniazid (H; 10 mg/kg), E (100 mg/kg), rifampicin (RIF; 10 mg/kg), and pyrazinamide (PZA; 150 mg/kg) (2HRZE/4HR) in BALB/c mice resulted in significantly faster clearance of organisms from the lungs, curing 100% of mice in just 3 months (15/15 culturenegative 6 months after discontinuation of therapy) (Figure 2.2-1) [18].

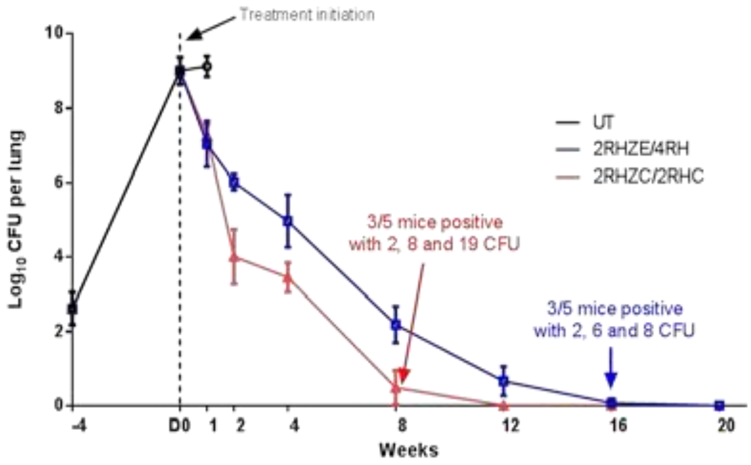

Figure 2.2-1: Antimicrobial effect of replacing ethambutol with clofazimine in standard TB therapy (RHZC) in BALB/c mice.

Further, among BALB/c mice (n = 307) aerosol-infected with *Mtb* H37Rv, replacement of RIF with rifapentine (RPT; 20 mg/kg) exhibited an additive effect

with CFZ (12.5 mg/kg/day, approximating a human dose of 100 mg/d) within a DS-TB regimen (Figure 2.2-2), achieving no detectable 6-month relapse following 12 weeks of treatment (Table 2.2-1) **[19]**.

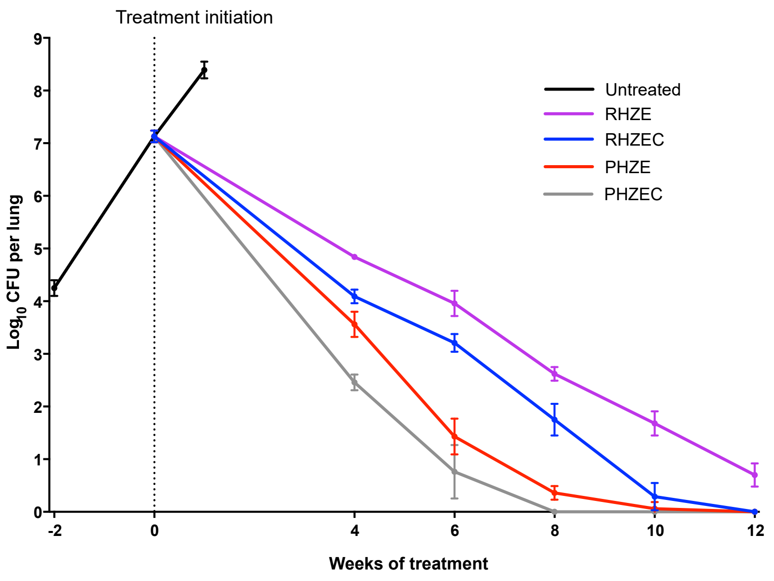

Figure 2.2-2: Additive effect of high-dose RIF within a clofazimine-based DS-TB regimen over 12 weeks [**19**].

Table 2.2-1: Sterilizing Activity of Rifapentine-clofazimine-containing DS-TB Regimen, BALB/c Mice [**19**]

Regimen	Proportion of mice relapsing 6 months after treatment for:			
	6 weeks	8 weeks	10 weeks	12 weeks
RHZE	---	---	---	19/20 (95%)
RHZEC	---	---	10/18 (56%)	0/20 (0%)
PHZE	---	6/20 (30%)	1/18 (6%)	0/18 (0%)
PHZEC	5/18 (28%)	0/18 (0%)	2/18 (11%)	0/20 (0%)

The majority of mice under stand-of-care treatment relapsed after 12 weeks of treatment; addition of clofazimine alone (RHZEC) resulted in about a 2-month treatment shortening; addition of RIF alone (PHZE) did better than clofazimine alone; addition of both RIF and clofazimine (PHZEC) resulted in a further relative 25% treatment shortening.

Reassuringly, sterilizing activity was similar among C3HeB/FeJ ("Kramnik") mice (Table 2.2-2), though mice with the most caseation seemed to benefit least from CFZ **[19]**. Thus, in both BALB/c and C3HeB/FeJ mice, high-dose RPT and CFZ have additive treatment-shortening effects, with a rank-order of PHZEC > PHZE > RHZEC > RHZE. Although head-to-head studies have not been performed, BALB/c experiments further suggest that (1) high-dose RPT (20 mg/kg) would be more effective than high-dose RIF, given that RIF 40 mg/kg appears to be dose-equivalent with RPT 10 mg/kg [**20**]; and (2) a PHZEC regimen has similar efficacy as a registered-PA-824-moxifloxacin-pyrazinamide regimen, and is likely marginally better than registered-PA-824-pyrazinamide and registered-moxifloxacin-pyrazinamide [**21**, **22**].

Table 2.2-2: Sterilizing Activity of Rifapentine-clofazimine-containing DS-TB Regimen, C3HeB/FeJ ("Kramnik") Mice [**19**]

Regimen	Proportion of mice with culture-positive lungs 6 months after treatment for:			
	6 weeks	8 weeks	10 weeks	12 weeks
RHZE	---	---	---	11/19 (55%)
RHZEC	---	---	7/17 (41%)	3/18 (17%)
PHZE	---	10/18 (56%)	0/17 (0%)	2/20 (10%)
PHZEC	5/17 (29%)	1/18 (6%)	1/18 (6%)	0/18 (0%)

Prior C3HeB/FeJ mouse experiments had demonstrated poor efficacy [**23**], hypothesized to be due to ineffective killing of extracellular *Mtb* residing within the caseous necrotic centers of granulomata, a finding potentially supported by matrix-assisted laser desorption/ionization mass spectrometry imaging studies [**24**]. However, PZA likewise performs poorly in C3HeB/FeJ mice (due to pH dependence) [**25**], despite substantial sterilizing activity in clinical trials [**26**].

As murine studies indicate a dose-dependent additive effect of CFZ during intensive phase DS-TB treatment, they also suggest dose-dependent antagonism in the absence of pyrazinamide/ethambutol (i.e., low-dose CFZ (between 1-3 mg/kg) resulted in higher CFU at 3-4 months) during the continuation phase (Figure 2.2-3, Table 2.2-3) **[19]**. The mechanism of antagonism is poorly understood, but may be due to induction of RIF clearance (e.g., through effects on major ABC transporters [**27**], such as P-glycoprotein). It is not known whether such antagonism would also occur during co-administration with RPT, rather than RIF.

These results collectively suggest a potent antimicrobial effect for CFZ. We hypothesize that use of CFZ in combination with other drugs with potent sterilizing activity, such as RPT and PZA, may powerfully synergize in a way not seen with second-line companion drugs to effect substantial treatment shortening for DS-TB.

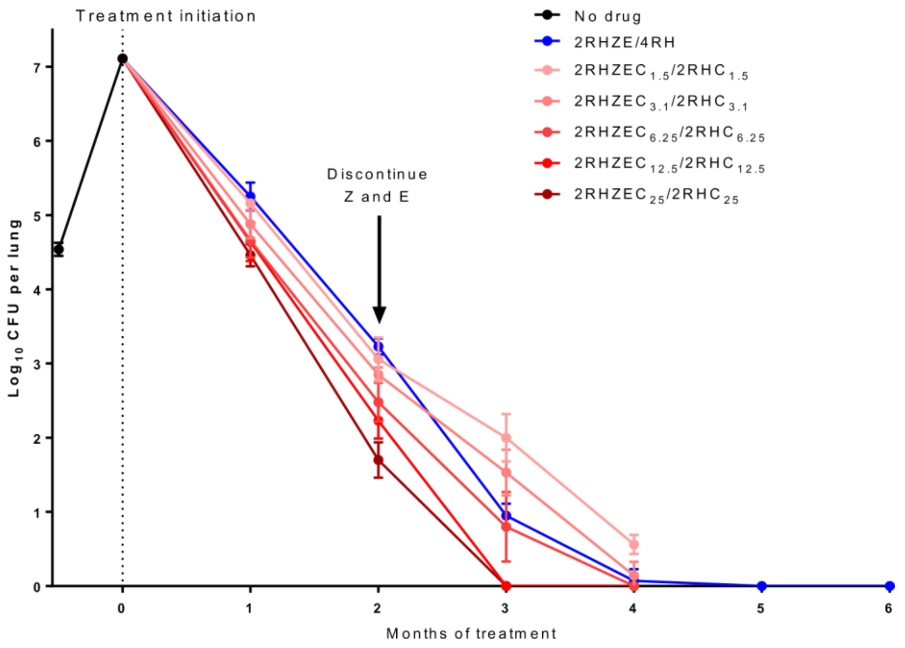

Figure 2.2-3: Decline in lung CFUs in mice treated with clofazimine plus RHZE for 12 weeks [**19**].

Table 2.2-3: Dose-dependent Effects of Clofazimine on DS-TB Regimens

Regimen	Proportion of mice relapsing after treatment for the following durations:			
	3 months		4 months	
	Study 1	Study 2	Study 1	Study 2
2RHZE/4RH	---	---	---	5/14
4RHZE	---	---	---	6/14
$2RHZEC_{1.5}/2RHC_{1.5}$	14/14	---	13/14	---
$2RHZEC_{3.1}/2RHC_{3.1}$	13/15	14/15	5/15	11/14
$4RHZEC_{3.1}$	---	14/15	---	2/11
$2RHZEC_{6.25}/2RHC_{6.25}$	6/15	9/15	0/14	0/15
$4RHZEC_{6.25}$	---	7/15	---	0/15
$2RHZEC_{12.5}/2RHC_{12.5}$	1/15	0/14	0/14	0/15
$4RHZEC_{12.5}$	---	1/14	---	0/15
$2RHZEC_{25}/2RHC_{25}$	2/15	---	0/15	---

2.2.1 Clofazimine Resistance and Potential Cross-resistance with Bedaquiline

Despite extensive use, development of CFZ resistance is conspicuously rare in leprosy [**28**-**31**]. Further, in vivo *Mtb* CFZ resistant strains have been described rarely, and the prevalence of various molecular mechanisms of resistance is largely unknown. To our knowledge, no CFZ-resistant clinical isolates have been reported; however, clinical strains resistant to BDQ have recently demonstrated cross-resistance to CFZ [**32**]. Reduced susceptibility to both CFZ and BDQ, due to de-repression of the *MmpS5-MmpL5* efflux pump (non-target based mutations in *Rv0678*) or loss-of-function mutations in *pepQ* (encoding a cytoplasmic prolinespecific aminopeptidase), has been demonstrated in mouse models, in patient cohorts treated with BDQ, and generated in vitro [**32**-**36**].

2.2.2 Clofazimine Pharmacology

CFZ's proposed antimicrobial mechanism of action includes redox cycling and toxic production of reactive oxygen species superoxide and H_2O_2. In addition, there is increased lysophospholipid production resulting in inhibition of cellular uptake of K^+. These mechanisms interfere with adenosine triphosphate (ATP) production, resulting in bacterial cell wall dysfunction and death [**37**]. In addition, CFZ has anti-inflammatory effects thought to be due to decreased activation and proliferation of T-lymphocytes [**37**-**39**].

Minimal modern PK data exist to describe the disposition of, or important drugdrug interactions with CFZ. CFZ bioavailability after oral administration is variable (45-60%) and enhanced by co-administration with food [**40**, **41**]. A single 200 mg dose of CFZ results in a mean plasma concentration of 0.41 mg/L in healthy volunteers [**40**]; daily dosing of 100, 300, and 400 mg in leprosy patients results in average plasma concentrations of 0.7, 1.0, and 1.41 mg/L, respectively [**42**].

The metabolism of CFZ is not well characterized but is known to involve glucuronidation [**42**]. CFZ is primarily eliminated as unchanged drug in the feces, due to both biliary excretion and unabsorbed orally administered drug. A smaller fraction is eliminated in the urine as both unchanged drug and metabolites [**43**]. CFZ is >99% protein bound, primarily to beta-lipoproteins but also alphalipoproteins in serum (Clofazimine Investigator's Brochure, Edition 2, August 31, 2016). CFZ distributes extensively to the reticuloendothelial system and adipose tissue. In mice, this accumulating drug in the tissues results in the serum half-life extending from 1.45 to 8.19 weeks when CFZ administration is extended from 4 to 20 weeks [**44**]. In humans, the half-life of CFZ is not well characterized. After 8 days of dosing, CFZ plasma half-life was estimated to be 10.5 days [**41**]. However, it should be noted that this is not based on steady-state (which can take up to 110 days to achieve) [**45**]. Another early report estimated the elimination half-life of CFZ based on spectrographic analysis of urine from a small number of leprosy patients receiving high-dose CFZ as at least 69 days [**46**].

Intriguingly, CFZ has been characterized as a cytochrome P450 (CYP) 3A4 inhibitor in two in vitro studies [**47**, **48**], and by Novartis probe drug-drug interaction studies (Clofazimine Investigator's Brochure, Edition 2, August 31, 2016); a single study has also suggested that it may be a weak inducer of CYP3A4 at low concentrations [**36**]. CFZ is also an inhibitor of CYP2C8 and CYP2D6, but not CYP1A2, CYP2A6, CYP2B6, CYP2C9, CYP2C19, or CYP2E1 (Clofazimine Investigator's Brochure, Edition 2, August 31, 2016). Despite this, very few drug-drug interaction studies have been conducted. A recent population pharmacokinetic model elaborated by members of our protocol team did not find a statistically significant PK drug-drug interaction between CFZ and BDQ, a substrate of CYP3A4 [**49**]. One study of six subjects suggested that RIF T_{max} was delayed and half-life was increased by CFZ co-administration [**50**] but there was no resultant change in C_{max} or area under the curve (AUC); this suggests a likely minimal clinical impact, and CFZ and RIF are routinely used in combination for leprosy. Though CFZ and RPT have not been studied in combination, these data support the proposed combination.

Recently, CFZ pharmacokinetics was characterized after 14 days in an EBA study in combination with BDQ, PZA, and pretomanid [16]. CFZ was administered as 300 mg orally for 3 days, followed by 100 mg orally daily through day 14, with a resulting median C_{max} ranging from 229-268 ng/mL and AUC_{0-24} range 3742-4459 ng*h/mL. Notably, these parameters were lower than previously described after a single dose [**40**], highlighting the uncertainties related to the optimal CFZ dosing and resulting pharmacokinetic exposure in humans. Using intensive and sparse PK sampling, A5362 will contribute significantly to the understanding of CFZ over a treatment course for DS-TB.

Potential for CFZ-ART Drug-Drug Interaction

In drug-drug interaction studies conducted by Novartis, CFZ was a moderate inhibitor of EFV, RAL, and dolutegravir (DTG) metabolism, resulting in 2.12- to 3.26-fold higher antiretroviral therapy (ART) AUC (Clofazimine Investigator's Brochure, Edition 2, August 31, 2016). Notably, these increases in exposure would not typically prevent co-administration based on FDA regulatory guidelines; therefore, there are no contraindications to combining these ART regimens with CFZ. Importantly, this observation was in the absence of RIF or rifampicin, which are known inducers of these same medications, potentially mitigating the impact of CFZ inhibition. CFZ exposure in A5362 will be evaluated in participants receiving concomitant ART via population PK modeling using intensive and sparse samples of CFZ, though sample sizes will preclude specific study of ART PK in A5362.

Despite in vitro data indicating that CFZ is a CYP3A4 inhibitor, summarized in the preceding paragraph, the only in vivo data is a population PK study [**49**] showing no effect of CFZ on bedaquiline (BDQ) concentrations (BDQ is a CYP3A4 substrate). The two ART regimens that will be used with RIF/RPT are EFV dosed once daily and DTG dosed twice daily. EFV is a CYP2B6 and a minor CYP2A6 substrate; CFZ does not inhibit either of these enzymes, so no interaction is expected. DTG is primarily metabolized by UGT1A1, with only a

minor contribution from CYP3A4. It is possible that CFZ could increase DTG concentrations modestly, but this is unlikely, as boosted protease inhibitors (which strongly inhibit CYP3A4) do not significantly alter DTG exposure. Therefore, there is no evidence of a drug-drug interaction that could reduce antiretroviral concentrations and result in ART failure.

2.2.3 Clofazimine Dosing

CFZ is available as 50 mg and 100 mg oral capsules. Despite longstanding use in leprosy, CFZ remains classified by the Food and Drug Administration (FDA) as an investigational drug without an indication for TB. Adult dosing for multi-bacillary leprosy is 50 mg daily for 12 months (21.6g total dose, WHO), or 24 months (36g total dose, National Hansen's Disease Program) in combination with RIF and dapsone. Severe type II lepra reactions unresponsive to corticosteroids can be treated with CFZ 300 mg daily, potentially for months; the anti-inflammatory activity of CFZ in these cases is thought to be paramount.

As described above, murine models provide evidence of CFZ efficacy at doses similar to 100 mg and 200 mg per day in humans. In these models, loading doses of CFZ result in serum concentrations above the minimum inhibitory concentration (MIC) more quickly, but no increase in speed of mycobacterial killing. Novartis has described a desirable CFZ dosing interval to achieve the MDR-TB CFZ MIC_{99} of 1.18 mcg/mL as early as possible (approximately week 8) and to sustain it for the remainder of the 24-week intensive phase. Because of its extremely long half-life, the previously proposed dosing of CFZ in Novartis MDR-TB studies is 200 mg once daily for 18 weeks, followed by 100 mg once daily for the remaining 6 weeks of intensive therapy [**45**].

In internal studies, Novartis has determined CFZ's MIC_{99} against drug-sensitive, clinical TB isolates to be 0.18 to 0.84 mcg/mL, much lower than those desired for MDR-TB, which was 0.3 to 1.18 mcg/mL [**51**]. However, prior studies have demonstrated a lower MIC for CFZ in treating drug-susceptible and drugresistant *Mtb* (MIC_{90} ranging from 0.06-1.0 mcg/mL, with most tested strains between 0.12-0.25 mcg/mL) [15, **52**-**56**, **57**]. However, the surrogacy of plasma drug concentrations in representing the active compartment of interest is unclear, given high intracellular concentrations and prolonged redistribution due to a large volume of distribution estimated at 1470 liters. Although loading doses did not effect decline in lung *Mtb* CFU in BALB/c mice treated with CFZ monotherapy [**44**] (Figure 2.2.3-1), extant pharmacokinetic data in humans (Figure 2.2.3-2) and a murine human translational model [Rada Savic, personal communication] are supportive of loading doses, similar to other anti-TB drugs with long elimination half-lives (e.g., bedaquiline [Sirturo (Bedaquiline) Investigator's Brochure, January 2017]). Based on population PK modeling, loading doses of 300 mg daily for 2 weeks will reach a desired concentration of 250 ng/mL by the end of the loading period, while 100 mg daily throughout therapy will reach the same

concentration approximately 7 weeks into therapy [Rada Savic, personal communication]. Given the pharmacologic properties of clofazimine, and the desire to reach target concentrations as soon as possible for a treatment shortening strategy, we will administer a 2-week 300 mg per day load, followed by 100 mg daily. This dosing regimen was futher investigated through simulations with a population PK model developed on data from the ProBex study (MDR-TB patients **[58]**) and an EBA study (DS-TB patients [**59**]. The simulations confirmed that expected active concentrations will be reached substantially faster with the loading dose. The peak concentrations at the end of the loading phase may be higher than at steady state with CFZ dosed at 100 mg daily, but lower than what is seen at long-term dosing with 200 mg daily [Elin Svensson, personal communication].

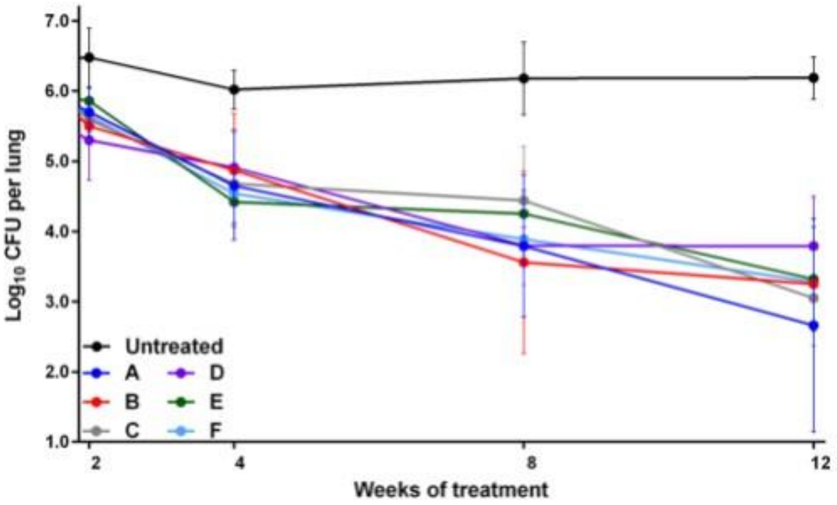

Figure 2.2.3-1: Decline in lung *Mtb* colony-forming units in mice treated with CFZ monotherapy with and without loading doses [**44**]. Dose-dependent concentrations of clofazimine in the blood and tissues did not translate into dose-dependent antimicrobial activity. *Doses*: A, 25 mg/kg daily; B, 12.5 mg/kg daily; C, 6.25 mg/kg daily; D (load), d1, 200 mg/kg; d2, 100 mg/kg; d3–14, 75 mg/kg daily; wk 2–12, 25 mg/kg; E (load), d1, 100 mg/kg; d2, 75 mg/kg; d3–14, 50 mg/kg daily; wk 2–12, 25 mg/kg; F (load), d1, 50 mg/kg; d2–14, 25 mg/kg daily; wk 2–12, 25 mg/kg.

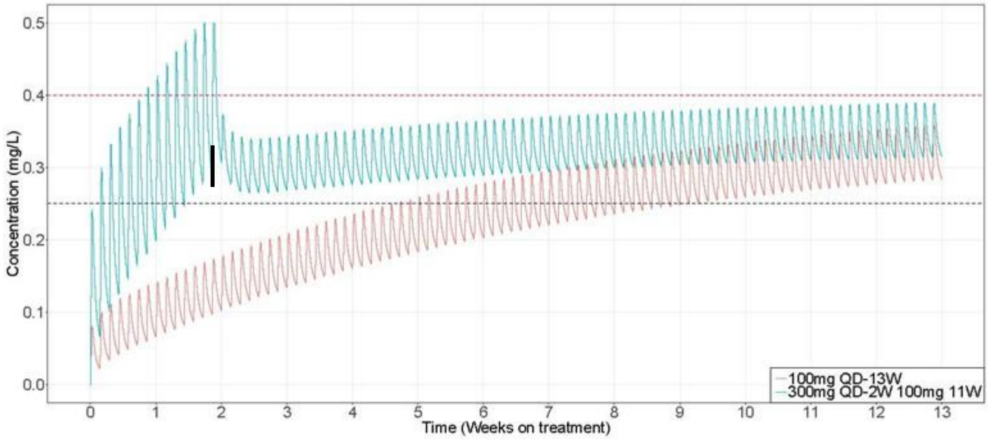

Figure 2.2.3-2: Simulated typical plasma concentration-time profiles of CFZ following multiple dosing of 100 mg daily in TB patients with and without loading phase (2 weeks 300 mg daily). Dashed lines represent (black) the suggested MIC target (0.25 mg/L) and (red) Cmax at steady for 100 mg QD in standard MDR treatment (9 months) [MT Abdelwahab et al., in manuscript].

Although existing evidence is supportive of the need for CFZ loading doses, the data are not definitive, and equipoise exists; this is evidenced by the multiple dosing strategies currently in practice and under evaluation (i.e., 100 mg daily with 300 mg daily 2-week loading dose in A5362; 200 mg daily in the TRUNCATE-TB trial [**60**]; 100 mg daily without load as WHO-recommended dosing for DR-TB [**61**]. In our trial, we propose a secondary objective to compare Arm C, a group of participants who receive the guideline- recommended dose of CFZ 100 mg daily, without a loading dose, to inform our understanding of the impact of the 2-week loading dose used in Arm 1. The impact of a loading dose on CFZ pharmacokinetics has not been evaluated outside of a modeled simulation, and this Arm will allow us, for the first time, to definitively reference the influence of time versus dose effect on how quickly CFZ achieves steady state and enable characterization of the suggested nonlinearity in bioavailability. Currently, it is unclear if this is caused by saturation of absorption at higher doses or by an induction phenomenon changing absorption over time. This will improve our ability to translate the findings of A5362 to other ongoing trials using different CFZ dosing.

2.2.4 Clofazimine Safety

CFZ is well tolerated and has been used in over 14 million patients with lepromatous leprosy over the past 40 years, usually at a dose of 50 mg daily for 12 to 24 months in conjunction with RIF and dapsone, as well as in thousands of patients with nontuberculous mycobacteria (NTM) disease within the United States.

The most common toxicities are non-melanotic hyperpigmentation (due to partitioning of the circulating, free base form of the drug into subcutaneous fat) and phototoxicity, ichthyosis, and gastrointestinal distress [**62**]. CFZ is a phenazine dye and causes a reddish-black discoloration of the skin which is slowly reversible after stopping treatment. In a recent clinical trial enrolling 2,912 participants with leprosy from India and China, CFZ-related pigmentation of the skin during 6 months of treatment was usually short-lived and acceptable to patients [**63**].

The prevalence of CFZ-associated skin discoloration demonstrates geographical variability. In studies from Bangladesh, Cameroon, and Niger, skin discoloration was unreported or reported in only 3.1% of the cases. [6, 13, **64**]. In other settings, hyperpigmentation has been much more commonly reported. In Peru, 93% of 27 MDR-TB cases treated with CFZ developed hyperpigmentation [**65**]. In China, hyperpigmentation reached 94.3% in one prospective clinical trial, [9] 23% in another [10], and 79% in a retrospective cohort [**66**]. Even in studies reporting high rates of discoloration, no participants in these settings discontinued CFZ.

A meta-analysis of cohort studies including 602 MDR-TB cases treated with CFZ reported a pooled proportion of adverse drug reactions requiring discontinuation of CFZ in 0.1% of the cases, comparable to that of first-line TB treatment. The two most reported adverse events were skin discoloration and gastrointestinal reactions. Cardiotoxicity was not systematically evaluated in the primary studies [**67**].

CFZ has the potential to prolong the QTc interval and may exacerbate QTc prolongation when given with other anti-TB agents such as fluoroquinolones or BDQ. In a recent study by Diacon [16], mean QTcF prolongation (~13ms) was reported among patients treated with CFZ alone; however, QTc interval prolongations remained within specified safety limits. No additive or synergistic QTc prolongation was seen when CFZ was combined with BDQ or PA824 after
2 weeks of exposure [16]. In a 24-week study, synergistic QTc prolongation of BDQ and CFZ was reported, but not associated with clinically relevant arrhythmia [**68**].

Studies of CFZ in patients with AIDS-related *Mycobacterium avium* complex found increased mortality when CFZ was added to clarithromycin. The QTc interval was not assessed, and individuals assigned to the CFZ arm had higher baseline mycobacterial load, implying that there may have been residual confounding despite randomization [**69**]. Increased mortality has not been replicated in animal models or in human studies of leprosy or MDR-TB.

2.2.5 Rifapentine Pharmacology

RPT is a semisynthetic rifamycin derivative used in combination with other antiTB agents for latent TB therapy and treatment of drug susceptible active pulmonary TB. RPT is bactericidal and binds the β-subunit of DNA-dependent RNA polymerase in *Mtb,* inhibiting synthesis of bacterial RNA. RPT has a microbiologic profile similar to that of RIF. Its structure differs from that of RIF by the presence of a cyclopentyl ring instead of a methyl group at the piperazinyl moiety. TBTC Study 29 demonstrated similar sputum culture conversion rates during substitution of RPT five times a week for rifampicin in standard intensive phase TB treatment regimens [**70**].

Absolute bioavailability of RPT has not been studied. RPT is well absorbed from the gastrointestinal tract, and relative bioavailability of 70% is achieved among individuals with TB compared to healthy volunteers. Food significantly increases absorption of RPT (bioavailability increased by 86% with high-fat meal. Administration with a high-fat meal increases the AUC and C_{max} of RPT (both 40 to 50%) compared to fasting conditions, and RPT should be administered with meals [Priftin package insert, 2018]. Maximum plasma concentrations occur within 5 to 6 hours following oral administration. RPT and its active 25-desacetyl metabolite are highly protein-bound (97 – 99% and 93%, respectively), primarily to albumin [**71**]. The apparent volume of distribution is 70.2 ± 9.1 L in tuberculosis patients [Priftin package insert, 2018]. RPT is hydrolyzed by arylacetamine deacetylase in the liver and blood to 25-desacetyl-rifapentine. This active metabolite contributes about 40% of the drug's overall activity. RPT and its active metabolite have half-lives from 13 to 17 hours. This is approximately four times longer than the half-life of RIF (4 hours), permitting intermittent dosing of RPT. RPT is primarily excreted in bile and eliminated in feces (70%). Less than 10% of RPT is excreted in the urine as unchanged drug.

The MIC for of RPT for activity against *Mtb* is 0.01 to 0.06 μg/ml [**71**]. RPT accumulates intracellularly and achieves intracellular concentrations that up to five times higher than RIF [**71**].

RPT, like other rifamycins, induces CYP3A4, 2C8, and 2C9, which can lead to more rapid metabolism and clearance of many drugs. Rifamycins are also known to induce the activity of phase II enzymes such as as glucuronosyl- and sulphotransferase and may reduce levels of drugs metabolized by those pathways.

2.2.5.1 Rifapentine Dosing

RPT is available as 150 mg tablets. For active pulmonary TB, RPT is used in combination with other anti-TB agents and dosed intermittently as 600 mg twice weekly during the intensive phase of therapy (2 months). RPT is continued as 600 mg once weekly during the continuation phase of

treatment (4 months) [Priftin package insert, 2018]. TBTC Study 29X (n=334) was a RPT dose-ranging extension (~10, 15, or 20 mg/kg) of TBTC Study 29 [**72**]. RPT was administered once daily for 7 days per week in combination with other anti-TB agents during intensive phase treatment compared to standard dose RIF. The study found 8-week culture conversion in the RPT arm (92.5%, 89.4%, and 94.7% in 10, 15, and 20 mg/kg RPT groups, respectively) than the RIF arm (81.3%).

2.2.5.2 Potential for Rifapentine-ART Drug-Drug Interaction

Interactions between RPT and EFV and DTG [**73**] have been studied with weekly RPT used in prevention [**74**]. There was no significant effect on EFV concentrations but, as expected, DTG trough concentrations were reduced by about 50%. Despite this, all participants maintained virologic suppression. It is expected that twice-daily DTG will overcome the induction by RPT, as is the case with RIF.

2.2.5.3 Rifapentine Safety

RPT, like other rifamycins, causes red-orange discoloration of body fluids and can stain contact lenses. In clinical trials in which RPT was combined with INH and other anti-TB drugs and administered once or twice weekly, rates of adverse reactions were similar with RIF and RPT, with increased liver aminotransferase activity in about 5% of patients. The only adverse effect that has occurred more often with RPT than with RIF has been hyperuricemia when the drug was given twice weekly; of note, hyperuricemia was attributed to PZA that was administered concomitantly. Other adverse reactions reported in 1-5% of patients included the following: hemoptysis, dizziness, hypertension, headache, gastrointestinal upset, rash, cytopenias, hematuria, pyuria, and proteinuria [Priftin package insert, 2018].

Adverse events are similar between RPT and RIF, including hepatotoxicity and rifamycin hypersensitivity syndrome [**71**]. In TBTC Study 29, 1.5% of participants in the RPT arm (n=275) discontinued RPT due to toxicities [**70**]. Three participants had either Grade 3 or 4 hepatitis in the RPT arm (1.1%), and similar proportions of participants in either the RIF or RPT group experienced hepatitis: 7 of 254 (2.8%) in the RIF group and 11 of 275 (4.0%, P = .48) in the RPT group. Frequencies of other side effects were similar between RPT and RIF arms. These included anemia (0.4%), neutropenia (1.5%), nausea or vomiting (0.7%), rash (2.2%). Increased dose RPT (n=81, 20 mg/kg) found similar adverse events rates as compared to RIF and RPT arms dosed at 10 or 15 mg/kg, including hepatitis (2.5%), neutropenia (3.7%), and pruritis/rash (1.2%) [**72**].

2.2.6 Pharmacogenetics of TB Medications

It is important to consider the impact of human genetic polymorphisms when studying efficacy, safety and PK of TB drugs. Among TB drugs, the pharmacogenetics of INH has most extensively been studied. Frequent polymorphisms in *NAT2* (which encodes N-acetyltransferase 2) predict increased plasma INH exposure [**75-78**], and have been associated with increased risk for INH hepatotoxicity [**79**, **80**]. Risk for INH-induced neuropathy may also increase with *NAT2* slow acetylator alleles [**81**, **82**]. Regarding other TB drugs, RIF is a substrate for organic anion-transporting polypeptide 1B1 (coded by *SLCO1B1*) [**83**], and one study associated an *SLCO1B1* polymorphism with RIF bioavailability in South Africans [**84**]. Parenteral aminoglycosides such as streptomycin can cause sensorineural hearing loss [**85-89**], and mitochondrial DNA mutations in the gene that encodes 12S ribosomal RNA confer increased risk [**85**, **88**, **90**, **91**]. CFZ is metabolized by glucuronidation. Although there is no information regarding the pharmacogenetics of CFZ, other drugs with glucuronidation (UGT) metabolism or associated adverse events do have known pharmacogenetic associations. Frequent polymorphisms in the gene UGT1A1 are associated with premature atazanavir discontinuation due to hyperbilirubinemia [**92-94**] and with approximately 50% increased plasma DTG exposure [**95**].

In addition, pharmacogenetics results in drug-drug interaction between INH and EFV. Rifampicin induces hepatic CYP 450 *2B6*, so should decrease plasma EFV exposure [**96**], but in patients with slow metabolizer genotypes in *CYP2B6* and *NAT2* prescribed RIF with INH, plasma EFV exposure increases [**97-99**]. This may be because high INH concentrations in *NAT2* slow acetylators inhibit *CYP2A6,* which is a necessary pathway for EFV clearance in *CYP2B6* slow metabolizers [**98-100**]. Given the above considerations, it is important to evaluate the potential impact of genetic polymorphisms on CFZ PK, efficacy and toxicity, as well as on potential drug-drug interactions involving CFZ.

2.2.7 Sputum Ribosomal RNA Synthesis (RS) Ratio

There is a need for new PD markers that maximize information gained from early-phase human clinical trials. More accurate PD markers would enable selection of the most efficacious regimens for testing in definitive Phase III trials [101, 102]. The RS ratio is a novel class of PD marker. Unlike culture-based measures, which evaluate mycobacterial load*,* the RS ratio may evaluate a physiologic state of *Mtb* potentially reflective of treatment regimen or other factors. Specifically, the RS ratio quantifies the abundance of *Mtb* precursor ribosomal RNA (rRNA) relative to total structural rRNA. The RS ratio provides a measure of ongoing *Mtb* synthesis of rRNA, a process fundamental to all bacteria.

Studies in vitro and in mice suggest that drug regimens that cure TB faster (i.e., greater sterilizing activity) suppress the RS ratio to a greater degree

than regimens with lesser sterilizing activity. Additionally, human and mouse studies demonstrate a dose-response relationship between concentrations of sterilizing drugs administered and suppression of the RS ratio [103] [Walter ND, et al. Unpublished observations on Mycobacterium tuberculosis precursor rRNA indicates treatment-shortening activity of drugs; 2020].

The RS ratio is currently being investigated as a candidate PD marker in A5349 [(TBTC S31) ClinicalTrials.gov Identifier: NCT02410772]. A5349 tests two experimental regimens including high-dose RPT (1200 mg daily) versus the standard of care regimen. A5362 will extend the findings from A5349 by determining the effect of adding CFZ to a high-dose RPT regimen. By including the RS ratio as an exploratory biomarker, A5362 will generate additional molecular information about the effect of CFZ in humans and will advance a practical PD marker that will enhance the design of future trials.

2.3 Rationale

The anti-leprosy drug CFZ contributes substantially to the sterilizing activity of TB regimens in mice and to the relapse-free cure of MDR-TB regimens in humans, but significant gaps in our knowledge concerning optimal dosing, PK-PD, safety and tolerability, and treatment duration remain. Anti-inflammatory, as well as antimycobacterial properties, rare drug resistance, abundant and reassuring safety data, and high intracellular accumulation within macrophages have maintained interest in the chemotherapeutic role of CFZ in light of a weak pipeline of alternative anti-mycobacterial agents. Reversible but potentially stigmatizing skin discoloration and known QTc prolongation continue to temper enthusiasm and delay conclusive drug trials. Release of the WHO Update to MDR-TB Treatment Guidelines in 2018 recommending treatment regimens with CFZ as a Category B drug reinforces the need for fundamental research related to the disposition, drug-drug interactions, and adverse effects related to CFZ. Although a 3-month treatment duration is unprecedented in modern TB trials, strong preclinical data, allowance to extend treatment in the absence of clinical resolution, and the close follow-up afforded by significant trial infrastructure reassure us that adverse patient outcomes will be minimal and not dissimilar from contemporaneous drugsusceptible TB treatment trials.

3.0 STUDY DESIGN

A5362 is a Phase IIc, open-label, randomized trial to study the impact of adding CFZ and substituting RPT for RIF compared with SOC combination TB therapy for participants with DS-TB. Participants will be stratified based on HIV status and the presence of advanced disease as determined by chest X-ray [**104**].

Participants will be randomized to Arm 1 and treated for 13 weeks (including a 2-week CFZ loading dose of 300 mg daily; Arm 2 and treated for 26 weeks; or Arm C and treated for 4 weeks (PK **only** subgroup).

- Arm 1 (Experimental): PHZE + CFZ 300 mg once daily for 2 weeks, PHZE + CFZ 100 mg once daily for 6 weeks; then PHZ + CFZ 100 mg once daily for 5 weeks
- Arm 2 (SOC): RHZE for 8 weeks; then RH for 18 weeks
- Arm C (PK only subgroup): PHZE + CFZ 100 mg once daily for 4 weeks; then on study, off study medications and treated according to SOC (RHZE for 4 weeks; RH for 18 weeks)

For participants who consent to intensive PK sampling, they will be randomized using a 2:1:1 randomization ratio (Arm 1:Arm 2:Arm C). For participants who do not consent to intensive PK sampling, they will be randomized using a 2:1 randomization ratio (Arm 1:Arm 2). The first 20 participants randomized to Arm 1 who also consent to intensive PK sampling will be registered to be in the Intensive PK Sampling subgroup. The first 20 participants randomized to each of Arm 1 and Arm 2 who also consent to answering an open-ended qualitative interview at the end of treatment will be registered to be in the Qualitative Interview subgroup. Participants in Arm 1 may consent to either or both intensive PK sampling and qualitative interviews, or they can decline both.

CFZ and RPT are investigational agents. Rifampicin (RIF or R), isoniazid (INH or H), pyrazinamide (PZA or Z), and ethambutol (EMB or E) are study-supplied standard TB drugs.
All participants in Arms 1, 2, and C will be followed from randomization to week 65.

The primary analysis will be a superiority comparison of time to 12-week liquid culture conversion in Arm 1 compared with Arm 2. Important secondary analyses include 65week favorable composite efficacy outcome, including cumulative relapse proportion versus 6-month SOC controls; and safety and tolerability (regimen discontinuation) in Arms 1 and 2.

4.0 SELECTION AND ENROLLMENT OF PARTICIPANTS

4.1 Inclusion Criteria

4.1.1 Pulmonary TB (among participants with or without history of prior TB treatment) identified within 5 days prior to entry by:

At least one sputum specimen positive for *M. tuberculosis* by molecular TB assay (Xpert) or line probe assay [LPA])

<u>OR</u>
At least one sputum specimen positive (1+ or greater) for acid-fast bacilli (AFB) on smear microscopy

<u>Note</u>: TB diagnosis for purposes of meeting inclusion criterion can be from a study testing laboratory or from an outside laboratory, as long as it is from a sputum sample collected within 5 days prior to entry.

4.1.2 Pulmonary TB diagnosed without known INH resistance (e.g., by LPA **or Xpert MTB/XDR**) and without known RIF resistance (e.g., by either LPA or Xpert **MTB/RIF or Ultra**).

Note: In cases later shown to be RIF or INH resistant post-enrollment by molecular test (if the LPA on direct sputum is initially invalid, it can later be followed up with LPA on the culture isolate), or in cases shown to be RIF and INH susceptible by molecular test but later confirmed to be INH or RIF resistant according to phenotypic DST, participants will discontinue study treatment. The documentation of INH and RIF susceptibility must be only from study testing laboratory.

4.1.3 Aged ≥18 years.

4.1.4 Absence of HIV-1 infection, as documented by any licensed rapid HIV test or HIV-1 enzyme or chemiluminescence immunoassay (E/CIA) test kit, within 30 days prior to entry

OR

HIV-1 infection, documented by any licensed rapid HIV test or HIV-1 E/CIA test kit at any time prior to entry and confirmed by a licensed Western blot or a second antibody test by a method other than the initial rapid HIV and/or E/CIA, or by HIV-1 antigen or plasma HIV-1 RNA viral load. Two or more HIV-1 RNA viral loads of >1,000 copies/mL are also acceptable as documentation of HIV-1 infection.

Note A: The term “licensed” refers to a US FDA-approved kit, which is recommended. For sites that are unable to obtain an FDA-approved kit, a kit that has been certified or licensed by an oversight body within the country and validated internally is acceptable.

Note B: WHO and Centers for Disease Control and Prevention (CDC) guidelines mandate that confirmation of the initial test result must use a test that is different from the one used for the initial assessment. A reactive initial rapid test should be confirmed by either another type of rapid assay or an E/CIA that is based on a different antigen preparation and/or different test principle (e.g., indirect versus competitive), or a Western blot or a plasma HIV-1 RNA viral load.

4.1.5 For participants living with HIV, CD4+ cell count ≥100 cells/mm^3, obtained within 30 days prior to study entry at any network-approved non-US laboratory that is IQA certified.

4.1.6 For participants living with HIV must be currently receiving or planning to initiate ART at or before study week 8.

Note A: For a list of study-permitted ARV medications, refer to Appendix B.

Note B: Dosing of study-permitted ART should be reflective of local standard of care based on WHO or national guidelines in combination with RIF- or RPTcontaining TB therapy.

4.1.7 A verifiable address or residence readily accessible to facilitate directly observed therapy **(DOT)**, and willingness to inform the study team of any change of address during the treatment and follow-up period.

4.1.8 The following laboratory values obtained at or within 5 days prior to entry by any US laboratory that has a Clinical Laboratory Improvement Amendments (CLIA) certification or its equivalent, or at any network-approved non-US laboratory that operates in accordance with Good Clinical Laboratory Practice (GCLP) and participates in appropriate external quality assurance programs.

- Serum or plasma alanine aminotransferase (ALT) ≤3 times the upper limit of normal (ULN)
- Serum or plasma total bilirubin ≤2.5 times ULN
- Serum or plasma creatinine ≤2 times ULN
- Serum or plasma potassium ≥3.5 mEq/L and ≤5.5 mEq/L
- Absolute neutrophil count (ANC) ≥650/mm^3
- Hemoglobin ≥7.0 g/dL
- Platelet count ≥50,000/mm^3

4.1.9 **For study candidates of child-bearing potential**, negative serum or urine pregnancy test within 5 days prior to entry by any US clinic or laboratory that has a Clinical Laboratory Improvement Amendments (CLIA) certification or its equivalent, or is using a point of care (POC)/CLIA-waived test, or at any networkapproved non-US laboratory or clinic that operates in accordance with Good Clinical Laboratory Practice (GCLP) and participates in appropriate external quality assurance programs.

4.1.10 Female participants of reproductive potential must agree not to participate in the conception process (i.e., active attempt to become pregnant, in vitro fertilization), and if participating in sexual activity that could lead to pregnancy, must agree to use at least one reliable nonhormonal method of contraception, as listed below, while on study treatment and for 30 days after stopping study medications.

Acceptable forms of contraception include:

- Condoms
- Intrauterine device or intrauterine system
- Cervical cap with spermicide

- Diaphragm with spermicide

Note: Hormonal birth control alone is not acceptable, as it may not be sufficiently reliable in combination with RPT or RIF.

4.1.11 Female participants who are not of reproductive potential must have documentation of menopause (i.e., at least 1 year amenorrheic), hysterectomy, or bilateral oophorectomy or bilateral tubal ligation.

Note: Participant-reported history is acceptable documentation.

4.1.12 Documentation of Karnofsky performance score ^{3}50 within 30 days prior to entry.

4.1.13 Documentation of either the presence or absence of advanced disease as determined by chest X-ray within 5 days prior to entry.

Note: Advanced disease will be defined as a total combined area of air-space disease, cicatricial atelectasis, and/or fibrosis across all lung fields equivalent to or greater than one entire lung, or one or more large cavitations (≥3cm). See section 6.3.11.

4.1.14 Ability and willingness of participant to provide informed consent.

4.2 Exclusion Criteria

4.2.1 More than 5 days of treatment directed against active TB for the current TB episode preceding study entry.

4.2.2 Pregnant or breast-feeding.

4.2.3 Unable to take oral medications.

4.2.4 Current receipt of clofazimine or bedaquiline or known receipt of clofazamine or bedaquiline at any time in the past.

4.2.5 QTcF interval >450 ms for men or >470 ms for women within 30 days prior to entry.

4.2.6 Weight <40 kg.

4.2.7 Current or planned use within 6 months following enrollment of one or more of the following medications: HIV protease inhibitors, HIV entry and fusion inhibitors, HIV non-nucleoside reverse transcriptase inhibitors (other than EFV), elvitegravir/cobicistat, bictegravir, quinidine, procainamide, amiodarone, sotalol, disopyramide, ziprasidone, or terfenadine.

4.2.8 Current extrapulmonary TB, in the opinion of the site investigator.

4.2.9 Current or history of known personal or family long QT syndrome.

Note: Family is considered a first-degree relative (i.e., parent, offspring, or sibling).

4.2.10 Known allergy/sensitivity or any hypersensitivity to components of study TB drugs or their formulation.

4.2.11 Active drug, alcohol use or dependence; or mental illness (e.g., major depression) that, in the opinion of the site investigator, would interfere with adherence to study requirements.

4.2.12 Known history of acute intermittent porphyria.

4.2.13 Other medical conditions (e.g., severe uncontrolled diabetes, liver or kidney disease, blood disorders, peripheral neuritis, chronic diarrhea) in which the current clinical condition of the participant is likely to prejudice the response to, or assessment of, treatment.

4.3 Study Enrollment Procedures

4.3.1 Prior to implementation of this protocol, and any subsequent full version amendments, each site must have the protocol and the protocol consent form approved, as appropriate, by their local institutional review board (IRB)/ethics committee (EC) and any other applicable regulatory entity (RE). Upon receiving final approval, sites will submit all required protocol registration documents to the DAIDS Protocol Registration Office (PRO) at the Regulatory Support Center (RSC). The DAIDS PRO will review the submitted protocol registration packet to ensure that all of the required documents have been received. Protocol activation may be required before each site can enroll any participants.

Site-specific informed consent forms (ICFs) will be reviewed and approved by the DAIDS PRO and sites will receive an Initial Registration Notification from the DAIDS PRO that indicates successful completion of the protocol registration process. A copy of the Initial Registration Notification should be retained in the site's regulatory files.

Upon receiving final IRB/EC and any other applicable RE approvals for an amendment, sites should implement the amendment immediately. Sites are required to submit an amendment registration packet to the DAIDS PRO at the RSC. The DAIDS PRO will review the submitted protocol registration packet to ensure that all the required documents have been received. Site-specific ICFs will not be reviewed and approved by the DAIDS PRO. Sites will receive an Amendment Registration Notification when the DAIDS PRO receives a complete registration packet. A copy of the Amendment Registration Notification should be retained in the site's regulatory files.

For additional information on the protocol registration process and specific documents required for initial and amendment registrations, refer to the current version of the DAIDS Protocol Registration Manual.

Once a candidate for study entry has been identified, details will be carefully discussed with the participant. The participant will be asked to read and sign the approved protocol consent form.

Participants from whom a signed informed consent has been obtained may be screened and enrolled, if they otherwise qualify. An ACTG Screening Checklist must be entered through the Data Management Center (DMC) Participant Enrollment System.

For participants in Arm 1 and Arm 2 who agree to participate in the open-ended qualitative interview evaluations **(Qualitative Interview subgroup)**, or for Arm 1 participants who agree to participate in the intensive PK evaluations **(Intensive PK Sampling subgroup)**, DM A5362 eligibility checklists **are** required through the DMC Participant Enrollment System.

4.3.2 Protocol Activation

Prior to enrollment, sites must complete the Protocol Activation Checklist found on the ACTG Member website. This checklist must be approved prior to any screening of participants for enrollment.

4.3.3 Participant Registration

For participants from whom informed consent has been obtained, but who are deemed ineligible or who do not enroll into the initial protocol step, an ACTG Screening Failure Results form must be completed and keyed into the database. Participants who meet the enrollment criteria will be randomized to the study according to standard ACTG DMC procedures.

4.4 Co-enrollment Guidelines

- Non-US sites are encouraged to coenroll participants in A5243, "Plan for Obtaining Human Biological Samples at Non-U.S. Clinical Research Sites for Currently Unspecified Genetic Analyses." Co-enrollment in A5243 does not require permission from the A5362 protocol chairs.
- For specific questions and approval for co-enrollment in other studies, sites should first check the A5362 PSWP or contact the protocol team via e-mail as described in the Study Management section.

5.0 STUDY REGIMEN

5.1 Regimens, Administration and Duration

5.1.1 Regimens

Participants will be randomized to Arm 1, 2, or C:

Table 5.1.1-1: Regimens for Arms 1, 2, and C. All study products will be given once daily.

Weeks	Arm 1 (Experimental)	Arm 2 (SOC)	Arm C (PK only subgroup)
0 – 2	Rifapentine 1200 mg<br>Isoniazid 300 mg<br>Pyrazinamide weight-based dosing*<br>Ethambutol weight-based dosing*<br>Clofazimine 300 mg	Rifampicin 600 mg<br>Isoniazid 300 mg<br>Pyrazinamide weight-based dosing*<br>Ethambutol weight-based dosing*	Rifapentine 1200 mg<br>Isoniazid 300 mg<br>Pyrazinamide weight-based dosing*<br>Ethambutol weight-based dosing*<br>Clofazimine 100 mg
3 – 4	Rifapentine 1200 mg<br>Isoniazid 300 mg<br>Pyrazinamide weight-based dosing*<br>Ethambutol weight-based dosing*<br>Clofazimine 100 mg	Rifampicin 600 mg<br>Isoniazid 300 mg<br>Pyrazinamide weight-based dosing*<br>Ethambutol weight-based dosing*	Rifapentine 1200 mg<br>Isoniazid 300 mg<br>Pyrazinamide weight-based dosing*<br>Ethambutol weight-based dosing*<br>Clofazimine 100 mg
5 – 8	Rifapentine 1200 mg<br>Isoniazid 300 mg<br>Pyrazinamide weight-based dosing*<br>Ethambutol weight-based dosing*<br>Clofazimine 100 mg	Rifampicin 600 mg<br>Isoniazid 300 mg<br>Pyrazinamide weight-based dosing*<br>Ethambutol weight-based dosing*	*To remain on study, off study medications; will complete treatment per local SOC*
9 – 13	Rifapentine 1200 mg<br>Isoniazid 300 mg<br>Pyrazinamide weight-based dosing*<br>Clofazimine 100 mg	Rifampicin 600 mg<br>Isoniazid 300 mg	

Weeks	Arm 1 (Experimental)	Arm 2 (SOC)	Arm C (PK only subgroup)
14 – 26	No treatment	Rifampicin 600 mg Isoniazid 300 mg	

*Dosed based on weight, refer to Table 5.1.1-2.

Table 5.1.1-2: Weight-based Dosing for Pyrazinamide and Ethambutol

Weight Band	Pyrazinamide Daily Dose	Number of Pyrazinamide 500 mg tablets per daily dose	Ethambutol Daily Dose	Number of Ethambutol 400 mg tablets per daily dose
40 to <55 kg	1000 mg	2	800 mg	2
55 to <71 kg	1500 mg	3	1200 mg	3
≥71 kg	2000 mg	4	1600 mg	4

5.1.2 Administration

CFZ 100 mg: will be administered as one capsule orally once daily with food.

CFZ 300 mg: will be administered as three 100 mg capsules orally once daily with food.

RPT 1200 mg: will be administered as eight 150 mg tablets orally once daily with food. Tablets may be crushed and added to semi-solid food.

INH 300 mg: will be administered as one tablet orally once daily. All participants must receive pyridoxine (vitamin B6) with each dose of INH based on current local, national or international dosing guidelines.

PZA: will be administered based on weight (Table 5.1.1-2) orally once daily.

EMB: will be administered based on weight (Table 5.1.1-2) orally once daily.

RIF 600 mg: will be administered as two 300 mg capsules orally once daily on an empty stomach, 1 hour before or 2 hours after eating.

PHZE + CFZ may be administered at the same time, with food.

PHZ + CFZ may be administered at the same time, with food.

RHZE may be administered at the same time on an empty stomach, 1 hour before or 2 hours after eating.

RH may be administered at the same time on an empty stomach, 1 hour before or 2 hours after eating.

If a daily dose is missed, the participant will be instructed to administer the missed dose as soon as possible, unless the next dose is due within 6 hours. If the next dose is due within 6 hours, the missed dose will be skipped and the next dose will be administered as originally scheduled. Participants should not double the next dose of study drug in order to "make up" what had been missed.

5.1.3 Duration

Participants will be treated for 13 weeks (Arm 1), or 26 weeks (Arm 2), then followed post-treatment to week 65. Participants will be treated for 4 weeks in Arm C, then complete treatment according to SOC, and be followed to week 65.

5.2 Formulations and Preparation

5.2.1 Clofazimine (CFZ or C): 100 mg capsules. Do not store above 25ºC (77ºF). Protect from moisture. Dispense in a tight container.

5.2.2 Rifapentine (RPT or P): 150 mg tablets. Store at 25°C (77°F) with excursions between 15° and 30°C (59°-86°F) permitted (see USP Controlled Room Temperature). Protect from excessive heat and humidity.

5.2.3 Isoniazid (INH or H): 300 mg tablets. Store below 30°C (86°F) and in the original container.

5.2.4 Pyrazinamide (PZA or Z): 500 mg tablets. Store below 30°C (86°F). Protect from moisture.

5.2.5 Ethambutol (EMB or E): 400 mg tablets. Store below 30°C (86°F) and in the original container.

5.2.6 Rifampicin (RIF or R; also referred to as rifampin): 300 mg capsules. Store at a temperature not exceeding 25°C, in a dry place, protected from light. Store capsules in blisters in the provided carton.

5.4 Concomitant Medications

Whenever a concomitant medication or study product is initiated, or a dose changed, investigators must review the concomitant medication's and study product's most recent package insert, Investigator's Brochure, or updated information from DAIDS to obtain the most current information on drug interactions, contraindications, and precautions.

Additional drug information may be found on the updated ACTG Precautionary and Prohibited Medications Database located at: http://tprc.pharm.buffalo.edu/home/di_search/.

5.4.1 Required Medications

See section 4.1.6 and Appendix B for specifications about ART. Changes in ART must be discussed with the A5362 protocol team during the study product dosing period.

While on INH, participants must receive pyridoxine (vitamin B6) with each dose of INH based on current local, national, or international dosing guidelines.

5.4.2 Prohibited Medications

For a list of prohibited medications, including those known to significantly prolong the QTc interval, see Appendix A.

6.0 CLINICAL AND LABORATORY EVALUATIONS

5.3 Pharmacy: Product Supply, Distribution, and Accountability

5.3.1 Study Product Acquisition/Distribution

Clofazimine (Lamprene®) will be manufactured by Novartis.

Rifapentine (Priftin®) will be manufactured by Sanofi.

Isonazid, pyrazinamide, ethambutol and rifampicin will be manufactured by Macleods Pharmaceuticals.

Study products will be purchased through the ACTG Leadership and Operations and made available through the NIAID Clinical Research Products Management Center (CRPMC). The site pharmacist should obtain the study product(s) for this protocol by following the instructions in the Pharmacy Guidelines and Instructions for DAIDS Clinical Trials Networks.

Antiretroviral medications are NOT provided through the study and must be obtained locally by the site. Selection and dosing of ART in combination with RIF or RPT should adhere to local guidelines at each site.

Pyridoxine (vitamin B6) will NOT be provided through the study and must be obtained locally by the site.

5.3.2 Study Product Accountability

The site pharmacist is required to maintain complete records of all study products received from the NIAID CRPMC and subsequently dispensed. The site pharmacist at non-US CRSs must follow the instructions in the Pharmacy Guidelines and Instructions for DAIDS Clinical Trials Networks for the destruction of unused study products.

6.1 Schedule of Evaluations

Table 6.1-1: Schedule of Evaluations for Arms 1 and 2

Evaluation	Screening (-30 days)	Entry (week 0)	Day 3 (±1 day)	Treatment Weeks (Visit Window ±3 days)[14]												Follow-up (Visit Window is ±7 days)				Time of Suspected Treatment Failure or Recurrence or Poor Treatment Response	Premature Treatment /Study Discontinuation
				1[1]	2	3[1]	4	6	8	10	12	13	17	21	26	34	42	52	65		
Documentation of HIV-1	X																				
Medical History	X	X																			
Medication History	X	X																			
Complete Physical Exam	X																				
**Stored Serum**		**X**										**X**[15]			**X**[2]				**X**	**X**	**X**
Targeted Physical Exam		X	X	X	X	X	X	X	X	X	X	X	X	X	X	X	X	X	X	X	X
Hyperpigmentation Assessment		X							X			X			X				X	X	X
Concomitant Medications	X	X	X	X	X	X	X	X	X	X	X	X	X	X	X	X	X	X	X	X	X
Hematology	X[3]						X		X			X			X						
Liver Function Tests	X[3]						X		X			X			X						
Chemistry	X[3]		X	X	X		X		X			X			X						
Diabetes Testing		X																			
Pregnancy Testing	X[3]	Required at week 13 for Arm 1; otherwise as clinically indicated																			
Urinalysis		X																			
Plasma HIV-1 RNA (if HIV+)		X									X										
CD4+ (if HIV+)	X																				
Sputum AFB Smear and Culture[4]	X[3]	X		X	X	X	X	X	X	X	X	X	X	X	X	X	X	X	X	X	X
Sputum **RS Ratio**[5] **(optional)**		X		X	X	X	X													X	**X**

Evaluation	Screening (-30 days)	Entry (week 0)	Day 3 (±1 day)	Treatment Weeks (Visit Window ±3 days)[14]												Follow-up (Visit Window is ±7 days)				Time of Suspected Treatment Failure or Recurrence or Poor Treatment Response	Premature Treatment /Study Discontinuation
				1[1]	2	3[1]	4	6	8	10	12	13	17	21	26	34	42	52	65		
Molecular TB Diagnostic Assay (to detect *Mtb*)	X[3]																				
Molecular Assay to Detect *Mtb* INH- and RIF- Resistance	X[3]																			X	X
Whole Genome[6] Sequencing		X																		X	
Drug-Susceptibility Testing[7]		X									X	X	X	X	X	X	X	X	X	X	X
Chest X-ray	X[3]								X			X			X[2]					X	
ECG	X[3]		X		X[8]				X			X[8]									X
Intensive PK Sampling[9]					X							X									
Sparse PK Sampling[10]					X[11]		X		X		X	X[11]	X	X	X	X				X	X
Serum for Lipoprotein Measurement					X		X		X		X	X	X	X	X	X				X	X
Pharmacogenetics[12]		X																			
**DOT**				X	X	X	X	X	X	X	X	X	X[2]	X[2]	X[2]						
WHO Quality of Life Questionnaire		X							X			X			X				X		X
Depression Scale		X							X			X			X				X		X
Open-Ended Qualitative Interview[13]												X			X						

1 Arm 1 intensive PK sampling group only: Week 1 and Week 3.

2 Arm 2 only.
3 Must be obtained within 5 days prior to study entry.
4 Two sputum samples for AFB smear microscopy and culture in liquid and solid media will be collected at each time point. Sputum specimens and culture isolates will be stored for targeted deep sequencing and phenotypic drug-susceptibility testing in the event of TB treatment failure or TB recurrence.
5 **For sites that have optionally completed the RS ratio training and material procurement,** Arm**s** 1 **and 2, sputa sample will be collected at Entry, Weeks 2 and 4, and, if applicable, at Suspected Treatment Failure or Recurrence or Poor Treatment Response or Premature Treatment/Study Dicontinuation visits; for the** intensive PK **Sampling sub**group**, sputa will also be collected** at Week 1 and Week 3.
6 For participants with suspected recurrence, whole genome sequencing performed on the baseline and recurrent isolate pair.
7 **See section 6.3.9**.
8 ECG should be performed 6-8 hours after CFZ dosing in Arm 1 during the week 2 and week 13 intensive PK visit. 9 Arm 1 intensive PK sampling **sub**group only. See section 11.2.1 for sampling time points.
10 Arm 1 only. See section 11.2.2 for sampling time points.
11 Sampling at these visits will occur only for those not assigned to complete intensive PK visit at the same study week.
12 See section 6.3.14.
13 Qualitative interview will be among a subset of participants in Arm 1 at week 13, and in Arm 2 at week 26.
14 Visit windows cannot extend beyond last day of treatment. Visit must take place while participant is still on treatment.
15 **Arm 1 only.**

Table 6.1-2: Schedule of Evaluations for Arm C

Evaluation	Screening (-30 days)	Entry (week 0)	Day 3 (±1 day)	Treatment Weeks (Visit Window ±3 days)[10]				Follow-up (Visit Window is ±7 days)			Time of Suspected Treatment Failure or Recurrence or Poor Treatment Response	Premature Treatment/ Study Discontinuation
				1	2	3	4	8	26	65		
Documentation of HIV-1	X											
Medical History	X	X										
Medication History	X	X										
Complete Physical Exam	X											
**Stored Serum**		**X**										
Targeted Physical Exam		X	X	X	X	X	X	X	X	X	X	X
Concomitant Medications	X	X	X	X	X	X	X	X	X	X	X	X
Hematology	X[1]						X					
Liver Function Tests	X[1]						X					
Chemistry	X [1]		X	X	X		X					
Diabetes Testing		X										
Pregnancy Testing	X[1]	Required at week 4; otherwise as clinically indicated										
Urinalysis		X										
Plasma HIV-1 RNA (if HIV+)		X						X				
CD4+ (if HIV+)	X											
Sputum AFB Smear and Culture[2]	X[1]	X		X	X	X	X				X	X
Sputum **RS Ratio**[3] **(optional)**		X		X	X	X	X				X	**X**
Molecular TB Diagnostic Assay (to detect *Mtb*)	X											

Molecular Assay to Detect *Mtb* INH- AND RIFResistance	X										X	
Evaluation	Screening (-30 days)	Entry (week 0)	Day 3 (±1 day)	Treatment Weeks (Visit Window ±3 days)[10]				Follow-up (Visit Window is ±7 days)			Time of Suspected Treatment Failure or Recurrence or Poor Treatment Response	Premature Treatment/ Study Discontinuation
				1	2	3	4	8	26	65		
Whole Genome[4] Sequencing		X									X	
Drug-Susceptibility Testing[5]		X									X	X
Chest X-ray	X[1]										**X**	
ECG	X[1]		X		X[6]							
Intensive PK Sampling[7]					X							
Sparse PK Sampling[8]							X					
Serum for Lipoprotein Measurement					X		X					
Pharmacogenetics[9]		X										
**DOT**				X	X	X	X					
Depression Scale		X						X				

1 Must be obtained within 5 days prior to study entry.
2 Two sputum samples for AFB smear microscopy and culture in liquid and solid media will be collected at each time point. Sputum specimens and culture isolates will be stored for targeted deep sequencing and phenotypic drug-susceptibility testing in the event of TB treatment failure or TB recurrence.
3 **For sites that have optionally completed the RS ratio training and material procurement, s**putum sample at each **indicated** visit will be collected for **determination of RS ratio.**
4 For participants with suspected recurrence, whole genome sequencing performed on the baseline and recurrent isolate pair.
5 **See section 6.3.9.**
6 ECG should be performed 6-8 hours after CFZ dosing during the week 2 intensive PK visit.
7 Intensive PK sampling: See section 11.2.1 for sampling time points.
8 Sparse PK sampling. See section 11.2.2 for sampling time points. 9 See section 6.3.14.
10 Visit windows cannot extend beyond last day of treatment. Visit must take place while participant is still on treatment.

6.2 Timing of Evaluations

6.2.1 Screening Evaluations

Screening evaluations must occur prior to the participant starting any study medications, treatments, or interventions.

Screening
Screening evaluations to determine eligibility must be completed within 30 days prior to study entry unless otherwise specified.

Note: Screening evaluations for the identification of pulmonary TB and sputum samples collected for molecular and phenotypic drug susceptibility testing must be completed within 5 days prior to study entry.

In addition to data being collected on participants who enroll into the study, demographic, clinical, and laboratory data on screening failures and participants who do not enroll will be captured in a Screening Failure Results form and entered into the ACTG database.

6.2.2 Entry Evaluations

Entry evaluations can occur on the same day as screening evaluations if all screening criteria have been met prior to enrollment and prior to randomization. Participants must not have taken more than 5 days of total TB treatment prior to starting study medications. Participants must begin study medications within 72 hours following randomization.

6.2.3 Post-Entry Evaluations

On-Treatment Evaluations Arm 1:
The window for study visit at day 3 is ± 1 day. The window for study visits during weeks 1-13 is ± 3 days. Visit window cannot extend beyond last day of treatment. Week 13 visit must take place while participant is still on treatment.

Arm 2:
The window for study visit at day 3 is ± 1 day. The window for study visits during weeks 2-26 is ± 3 days. Visit window cannot extend beyond last day of treatment. Week 26 visit must take place while participant is still on treatment.

Arm C:
The window for study visit at day 3 is ± 1 day. The window for study visits during weeks 1-4 is ± 3 days. Visit window cannot extend beyond last day of treatment. Week 4 visit must take place while participant is still on treatment.

Post-Treatment Evaluations
Arm 1: The window for study visits during weeks 17-26 is ± 3 days. The window for study visits during weeks 34-65 is ± 7 days.

Arm 2: The window for study visits during weeks 34-65 is ± 7 days.

Arm C: The window for study visits at weeks 8, 26 and 65 is ± 7 days.

Event Driven Evaluations

Participants whose screening, entry, and first post-entry visit sputum cultures all fail to grow *Mtb* complex, or whose molecular or drug susceptibility test results demonstrate resistance to INH or RIF after enrollment, must be discontinued from study treatment immediately and followed on study, off study drugs. Complete the Premature Treatment/Study Discontinuation Evaluations as indicated in section 6.1 at the time of discontinuing study treatment.

Suspected TB Treatment Failure or TB Recurrence, or Poor Treatment Response

For study purposes a possible poor treatment response evaluation is triggered by any one or more of the following, but not limited to:

a. Worsening signs and/or symptoms consistent with TB at or after week 13 (Arm 1) or week 17 (Arm 2, SOC)

b. Radiographic worsening consistent with TB at or after week 13 (Arm 1) or week 17 (Arm 2, SOC)

c. AFB sputum smear-positivity involving at least two separate sputa at or after week 13 (Arm 1) or week 17 (Arm 2, SOC)

d. The site investigator is considering extension of TB treatment beyond that of the participant’s assigned regimen

e. The site investigator is considering retreatment with any TB therapy after the participant has completed assigned study treatment

f. For a participant on assigned study treatment, the site investigator is considering a change in treatment for efficacy reasons (this does not apply to changes in treatment for pregnancy, temporary drug challenge, or toxicity).

Perform the evaluations according to section 6.1 as soon as feasible when a possible poor treatment response is suspected, and ideally prior to extension, change or re-start of TB medications (in consultation with the Steering Committee) if the participant’s clinical condition permits. Complete the Possible Poor Treatment Response case report form. For study purposes, those participants should continue to be followed in the study per section 6.1 for outcome determination unless the participant withdraws consent.

Persistent Clinical Disease and Extension of Treatment

In the event of poor clinical response at week 13 among Arm 1 participants, treatment extension to a maximum of 17 weeks (119 doses) will occur if, at week 13 (or after completion of 91 daily doses if treatment has been extended for missed doses), the participant has persistent symptoms consistent with ongoing

active TB without alternative, more likely explanation for those symptoms, along with at least one of the following: (a) lack of radiographic improvement, or presence of radiographic worsening consistent with TB; (b) week 8 culture result is not negative; or (c) AFB sputum smear-positivity at week 13 involving at least two separate sputa.

All decisions regarding treatment extension should be discussed with and approved by the A5362 Steering Committee before implementation. Perform the evaluations according to section 6.1, Time of Suspected Treatment Failure or Recurrence, as soon as feasible, and ideally before a restart or change in treatment regimen.

The study regimen should not be extended beyond 17 weeks from randomization (or after completion of 119 daily doses if treatment has been extended for missed doses). If the participant continues to have persistent clinical disease at week 17, TB treatment may be further extended by switching to SOC, guided by DST and Xpert MTB/RIF results obtained at week 13. For study purposes, those participants should continue to be followed in the study per section 6.1 for outcomes determination unless the participant withdraws consent.

If culture-positivity occurs at EOT (week 13 for Arm 1) in an otherwise well participant is not noted until week 19, approximately 6 weeks later, the participant will be retreated with local SOC anti-TB medications guided by DST and molecular DST results obtained at week 13.

Follow-up of Arm C

Although data are limited, what is known from drug-drug interaction studies (see section 2.2.2) suggest lack of adverse clinical impact related to combination of either CFZ-ART or CFZ-RIF. Further, murine studies suggest that PHZEC without CFZ loading doses is a more potent regimen than RHZE, therefore, the existing evidence supports the likelihood that Arm C participants will receive treatment enhanced beyond SOC, without known or expected drug-drug interactions that would influence the remaining SOC drugs. By extension, Arm C participants should be less likely to experience treatment failure than Arm 2 SOC participants. Nevertheless, monitoring of the trial will be comprehensive (detailed in section 10.5), including Data Safety and Monitoring Board (DSMB) oversight.

6.2.4 Discontinuation Evaluations

Evaluations for Randomized Participants Who Do Not Start Study Treatment All eCRFs must be completed and keyed for the period up to and including the entry visit. Participants who do not start study treatment will be taken off study with no further evaluations required.

Premature Treatment Discontinuation Evaluations

Participants who prematurely and permanently discontinue study drug will have the treatment discontinuation evaluations performed as noted in section 6.1 within 4 weeks

after discontinuation. Premature treatment discontinuation evaluations that overlap within 4 weeks of regularly scheduled evaluations do not have to be repeated.

If the participant discontinues study drug prematurely, the participant will be encouraged to continue on study and receive all evaluations per section 6.1 through week 65.

Premature Study Discontinuation Evaluations

Participants who prematurely discontinue from the study for any reason will have the study discontinuation evaluations performed as noted in section 6.1 prior to being taken off study.

Evaluations for Participants Who Die

All eCRFs must be completed and keyed for the period up to the week of death.

6.3 Instructions for Evaluations

Each study site and laboratory involved in this study will comply with the DAIDS policy on Requirements for Laboratories Performing Testing for DAIDS-Supported and/or Sponsored Clinical Trials, which is available at https://www.niaid.nih.gov/sites/default/files/laboratorypolicy1.pdf.

All clinical and laboratory information required by this protocol must be present in the source documents. Sites must refer to the Source Document Guidelines on the DAIDS website for information about what must be included in the source document: https://www.niaid.nih.gov/sites/default/files/daids-sourcedocpolicy.pdf.

All stated evaluations are to be recorded on the eCRF unless otherwise specified. Refer to section 7.0 for information on the DAIDS AE Grading Table and AE reporting of adverse events requirements.

6.3.1 Documentation of HIV-1

Section 4.1.4 specifies assay requirements for HIV-1 documentation. HIV-1 documentation is not recorded on the eCRF.

6.3.2 Medical History

The medical history must include all signs and symptoms regardless of grade and all diagnoses identified by the ACTG criteria for clinical events and other diagnoses regardless of grade within the past 30 days. In addition, the following diagnoses should be recorded regardless of when the diagnosis was made:

- AIDS-defining conditions
- Bone fractures
- Coronary heart disease
- Cancer (exclusive of basal/squamous cell skin cancer)
- Diabetes
- Tuberculosis
- Chronic hepatitis A
- Chronic hepatitis B

- Acute hepatitis
- Long QT syndrome
- History of, or ongoing, inflammatory skin disorder such as leprosy, eczema, psoriasis, lichen planus, or other skin rash
- History of intestinal obstruction
- Major depression
- **SARS-CoV-2 infection**

Any allergies to any medications and their formulations must also be documented.

6.3.3 Medication History

A medication history must be present, including start and stop dates. Alternative therapies and dietary supplements should be recorded on source documents only. The table below lists the medications that must be included in the history:

Table 6.3.3-1: Medication History

Medication Category	Complete History or Timeframe
TB therapy for the current episode	Complete History
Prior TB treatment	Complete History
ART (for participants living with HIV only)	Complete History
Immune-based therapy	Within 30 days prior to study entry
Blinded study treatment	Complete History
Prescription drugs for all acute and chronic diseases, including for treatment and/or prophylaxis of opportunistic infections	Within 30 days prior to study entry
Alternative therapies	Currently being taken
Dietary supplements	Currently being taken
Sex-hormone medications or sex-hormone analogues or antagonists*	Last 12 months

*Hormone-releasing intrauterine devices (e.g., Mirena inserted in the last 5 years); oral, injectable, implanted, or patch contraceptives; vaginal ring, creams, or inserts; estrogen, progesterone, or testosterone therapy; leuprolide or other synthetic gonadotropin-releasing hormone; tamoxifen, raloxifene, aromatase inhibitors or any other androgen, estrogen, or progesterone analogue or antagonist therapy.

6.3.4 Clinical Assessments

Complete Physical Exam
A complete physical examination will be performed at screening only and is to include, at a minimum, an examination of the skin, head, mouth, and neck; auscultation of the chest; cardiac exam; abdominal exam; examination of the lower extremities for edema and Karnofsky performance test. The complete physical exam will also include signs and symptoms, diagnoses, height, weight and vital signs (temperature, pulse, respiration rate, and blood pressure).

Targeted Physical Exam
At and after entry, a targeted physical examination is to include weight and vital signs (temperature, pulse, respiration rate, and blood pressure), and is to be driven by any previously identified or new adverse event/targeted condition (as described in below bullets) that the participant has experienced since the previous study visit.

Post-entry, see section 8.3 for collection requirements for pregnancy.

Post-entry, record the following targeted event regardless of grade:

- AIDS-defining conditions (refer to the CDC HIV Classification and the WHO Staging System for HIV Infection and Disease)
- Coronary heart disease
- Arrhythmia
- Cancer (exclusive of basal/squamous cell skin cancer)
- Diabetes
- Tuberculosis Immune Reconstitution Inflammatory Syndrome (IRIS)
- Chronic hepatitis C
- Chronic hepatitis B
- Uterine pregnancy
- Intestinal obstruction
- Major depression
- Inflammatory skin disorder such as leprosy, eczema, psoriasis, lichen planus, or other skin rash
- **SARS-CoV-2 infection**

Post-entry, refer to section 7.2 for AE reporting requirements.

Hyperpigmentation Assessment
A skin colorimeter will be used to take colorimetric images at four facial triangulated anatomic locations unobstructed by hair (forehead, left cheek, right cheek, chin**) and at the upper inner arm (for assessment of baseline pigmentation)** at time points specified in section 6.1. Clinical images (standard photographs, used for quality assurance and as a visual record of outcomes) will be obtained at the same anatomic locations in sequence with the colorimeter under reproducible settings and lighting. Colorimetric measurements will be manually entered on an eCRF. Refer to the A5362 Manual of Procedures (MOPS) for details.

Subjective: Participant-reported changes in skin pigment and distress specifically related to these changes will be assessed by verbal response to a 10-point numeric rating scale (NRS):

- Skin pigment change: 0 (none) to 10 (most significant possible)
- Distress related to skin pigment changes: 0 (none) to 10 (worst possible)

Concomitant Medications

Post-entry, the following new and discontinued concomitant medications must be recorded on the eCRFs:

- Sex-hormone medications or sex-hormone analogues or antagonists (see section 6.3.3 for examples).
- All prescription medications, excluding study-provided TB medications.
    - **All study-provided TB medications for Arms 1 and 2 should not be recorded as Concomitant Medications.**
    - **All study-provided TB medications for Arm C weeks 0-4 should not be recorded as Concomitant Medications. ○ Site-provided standard of care TB medications for Arm C after week 4 should be recorded as Concomitant Medications.**

The following concomitant medications must be present only in the source documents:

- Nonprescription medications

Study**-provided** TB Medication Modifications

Record **as study treatment** all study-provided TB medication modifications, including initial doses, participant-initiated and/or protocol-mandated modifications, inadvertent and deliberate interruptions since the last visit. Record any permanent discontinuation of treatment.

6.3.5 Laboratory Evaluations

At screening and entry all laboratory values must be recorded on the eCRF. For post-entry assessments, record on the eCRF all laboratory values regardless of grade and refer to section 7.2 for AE reporting requirements for abnormal laboratory findings.

Hematology

Hemoglobin, white blood cell count (WBC), differential WBC (to include only neutrophils, lymphocytes, and monocytes), absolute neutrophil count (ANC), and platelet count will be performed in real time at the local laboratory.

Liver Function Tests

AST [SGOT], ALT [SGPT], total and direct bilirubin, and alkaline phosphatase will be performed in real time at the local laboratory.

Blood Chemistry

Lipase, creatine kinase, electrolytes (potassium, magnesium, calcium), and creatinine will be performed in real time at the local laboratory.

Diabetes Mellitus Test

Hemoglobin A1c (if unavailable, fasting blood glucose) will be performed at the local laboratory. If fasting blood glucose must be performed rather than Hemoglobin A1c, participants who are not fasting at the time of blood glucose

ascertainment (e.g., at baseline) will be counseled to fast prior to their next (e.g., week 1 or week 2) visit.

Pregnancy Test

For women of reproductive potential: Serum or urine b-HCG may be used (urine test must have a sensitivity of ≤25 mIU/mL). Record pregnancy and pregnancy outcome per section 8.3.

Urinalysis

A dipstick urinalysis will be performed at entry to assess proteinuria and hematuria.

6.3.6 Virologic Studies

Plasma HIV-1 RNA (if HIV positive)

Obtain HIV-1 RNA from a laboratory that is VQA approved.

6.3.7 Immunologic Studies

CD4+ (if HIV positive)

Obtain absolute CD4+ count within 30 days prior to study entry from a laboratory that possesses IQA certification.

6.3.8 Stored Serum

Stored serum will be collected at entry (all participants), at end of treatment (weeks 13 and 26 for Arms 1 and 2, respectively), and at end of follow-up (week 65, Arms 1 and 2 only) and may be analyzed for SARS-CoV-2 serology within a central laboratory at the end of the study if indicated to assess study outcomes.

6.3.**9** TB Laboratory Diagnostics and Microbiology

Sputum AFB Smear and Mycobacterial Culture in Liquid/Solid Media Two sputum samples will be collected for smear and culture in liquid and solid medium within 5 days prior to study entry and at other time points noted in section 6.1. Sputum AFB smear must be reported per WHO criteria (i.e., negative, scanty, +, ++, +++). Bacterial load will be determined and recorded for all specimens using the time to positivity output automatically provided by the mycobacteria growth indicator tube (MGIT) system. For all cultures demonstrating growth of AFB, species will be identified to at least the level of *Mtb* complex versus non-TB mycobacteria. Sputum and isolates from positive cultures will be stored at the site laboratory or the local TB laboratory performing the TB testing (as appropriate to the site procedures) unless shipment to a central lab is requested. Refer to the A5362 MOPS for laboratory procedure details.

<u>Sputum **Ribosomal RNA Synthesis (RS) Ratio (Optional)**</u>
Sites and participants are not required to participate in this collection. Participant selection must be documented in the Sample Informed Consent. For sites that have completed the RS ratio training and material procurement, sputa will be collected **for the RS ratio at the intervals identified in section 6.1 to test this exploratory candidate molecular biomarker. Because of its putative role as a PD marker, the frequency of sample collection is higher** for **participants in Arm 1 and Arm C undergoing intensive PK than for other participants. Participants in the intensive PK Sampling subgroups will have sputa for RS ratio collected at entry, weeks 1, 2, 3, and 4, and, if applicable, at Suspected Treatment Failure or Recurrence or Poor Treatment Response or Premature Treatment/Study Discontinuation visits. All other participants not in the intensive PK Sampling subgroups will have sputa for RS ratio collected at entry, weeks 2 and 4, and, if applicable, at Suspected Treatment Failure or Recurrence or Poor Treatment Response or Premature Treatment/Study Discontinuation visits.**

<u>Molecular TB Diagnostic Assay (to detect *Mtb*)</u>
Where available, WHO-endorsed molecular diagnostic TB assays (Xpert, Hain GenoType MTBDR*plus* v2.0 or greater (LPA), performed within 5 days prior to study entry for initial identification of *Mtb* will be documented.

<u>Molecular Assay to Detect *Mtb* INH- and RIF- Resistance</u>
The detection of resistance to INH and RIF via LPA **or Xpert MTB/XDR or Xpert MTB/RIF or Ultra** is required within 5 days prior to study entry to determine eligibility, and in the event of suspected TB treatment failure, TB recurrence, poor treatment response, or premature treatment or study discontinuation.

<u>Whole Genome Sequencing (WGS)</u>
All relapse isolates will undergo WGS according to internationally accepted protocols to determine molecular genotyping relative to baseline isolates. (https://support.illumina.com/sequencing/sequencing_kits/nextera-dna-flexkit.html)

<u>Drug-Susceptibility Testing (DST)</u>
For all participants, phenotypic (i.e., culture-based) DST for **INH and RIF** will be performed **at entry** on *Mtb* isolates collected using the MGIT system as indicated in section 6.1.

For participants experiencing treatment failure or relapse, the following tests will be perfomed on positive Mtb sputum cultures (1) at time points of treatment failure or relapse (i.e., Mtb isolates at or after the week 12 study visit for Arm 1, and at or after week 17 for Arm 2 and Arm C) for INH, RIF, PZA, CFZ, and BDQ, and (2) on stored baseline (i.e., entry) Mtb isolates for PZA, CFZ, and BDQ:

- **Phenotypic DST**
- **Minimal inhibitory concentrations (MICs)**
- **Genotypic DST (i.e., whole genome and targeted deep sequencing [single molecule-overlapping reads {SMOR}], see paragraph below).**

Because of challenges in interpretation of MIC values for PZA, CFZ, and BDQ (due to a relative lack of data from susceptible controls for these drugs), two additional samples will be used as control determinations for PZA, CFZ, and BDQ MICs. Mtb isolates collected at entry from two consecutive prior participants not known to have experienced a poor clinical outcome will undergo MIC testing for PZA, CFZ, and BDQ; if two consecutive prior participants with favorable outcome are not available, up to two consecutive subsequent participants with favorable outcome may be substituted.

Single molecule-overlapping reads (SMOR) analysis, a novel targeted deep sequencing approach, will be used to identify microbes resistant to CFZ, BDQ, and all SOC drugs representing ≥0.1% of a microbial community in longitudinal fashion [**105**]. Given storage and batched analysis, and the unknown nature and significance of small microbial-resistant subpopulations, SMOR results will not be used for clinical management in real-time.

6.3.**10** Possible Poor Treatment Response Evaluation

At the time of suspected TB treatment failure, TB recurrence, poor treatment response, or at the time the site investigator is considering extension of TB treatment beyond that of the participant's assigned regimen, participants will be evaluated for possible poor treatment response. Trigger for poor treatment response, type and date of poor response, treatment outcome, and confirmation of poor response will be documented.

6.3.1**1** Chest X-ray

The chest X-ray will be posterior-anterior at time points described in section 6.1. Extent of disease (limited to one lobe or region, unilateral, bilateral, or diffuse) and cavitation status (cavities present [location] or absent) will be documented by numerical score for grading chest X-ray in adult smear-positive pulmonary TB [**106**]. For purposes of randomization stratification, screening chest X-ray will be read, interpreted, and recorded at each site as advanced or non-advanced disease. Advanced disease will be defined as a total combined area of pulmonary opacity across all lung fields equivalent to or greater than one entire lung, or one or more large cavitations (≥3cm) [**104**]. Chest radiographs will be retrospectively reviewed in batched fashion centrally (in DICOM format by two Board Certified Chest Radiologists) for confirmation of advanced disease stratification and detailed findings. Refer to the A5362 MOPS for details.

6.3.1**2** ECG

Screening and longitudinal ECGs will be conducted at the study site according to section 6.1. At all visits, ECG will be performed in triplicate (collection of three ECGs 5-10 minutes apart). Specific instructions related to ECG procedures are described in the A5362 MOPS.

After entry, the ECG should be performed 6-8 hours after CFZ dosing in Arm 1 during intensive PK sampling at weeks 2 and 13, and in Arm C during intensive PK sampling at week 2. At all other visits, the ECG should be performed 4-8 hours after CFZ dosing.

To minimize the variability in QT intervals, ECG trace data are also automatically transmitted to the central or core ECG laboratory for determination of QT interval.

6.3.1**3** Pharmacokinetic Studies: Arm 1 and Arm C only

For visits occurring at week 13 or earlier, all PK sampling visits must be completed prior to discontinuation of study treatment. Sparse PK for CFZ will be collected after discontinuation of study treatment.

Intensive PK Sampling
Arm 1: 20 participants
Arm C: 20 participants
See sections 6.1 and 11.2.1 for designated PK visits and sampling time points.

Sparse PK Sampling
Arm 1: Participants not assigned to intensive PK
Arm C: 20 participants
See sections 6.1 and 11.2.2 for designated PK visits and sampling time points.

Serum for Lipoprotein Measurement
A single serum sample will be collected and stored for assessment of alpha- and beta-lipoprotein at each intensive or sparse PK visit.

6.3.1**4** Pharmacogenetics

A single whole blood sample will be obtained at entry from all study participants for genotyping of polymorphisms in human genes that may affect metabolism, disposition, and toxicity of study drugs as well as concomitant medications (e.g., *NAT2* and *CYP2B6*).

6.3.1**5** Directly Observed Therapy (DOT)

DOT will be performed throughout treatment. Each site must follow local TB guidelines about DOT. All drugs must be taken orally, 7 days per week. At least five doses per week must be administered as DOT by trained study outreach workers living close to the participant’s home. Doses taken on weekends and on

holidays may be under DOT or self-administered, as permitted by local TB guidelines.

6.3.16 Questionnaires

Questionnaires are posted on the DMC Portal in the Forms Management Utility.

WHO Quality of Life-BREF (WHOQOL-BREF): Arm 1 and Arm 2 only
The WHOQOL-BREF will be administered to assess how participants feel about their quality of life, health and other areas of their life, and will be conducted according to section 6.1.

Center for Epidemiologic Studies Depression Scale (CES-D)
The CES-D will be administered to screen for depression in participants, and will be conducted according to section 6.1.

6.3.17 Open-Ended Qualitative Interview: Arm 1 and Arm 2 only

An open-ended interview will be conducted in 20 consenting participants in each arm balanced by site to obtain ethnographic data on the impact of perceived skin color changes on their quality of life.

7.0 ADVERSE EVENTS AND STUDY MONITORING

7.1 Definition of Adverse Events

An adverse event (AE) is any unfavorable and unintended sign (including an abnormal laboratory finding), symptom, or diagnosis that occurs in a study participant during the conduct of the study REGARDLESS of the attribution (i.e., relationship of event to medical treatment/study product/device or procedure/intervention). This includes any occurrence that is new in onset or aggravated in severity or frequency from the baseline condition.

7.2 Adverse Event Collection Requirements for this Study

All AEs must be recorded on the eCRFs if any of the following criteria have been met.

- All AEs that led to a change in study treatment/intervention regardless of grade
- All AEs meeting SAE definition or EAE reporting requirement
- All Grade ≥3 AEs
- All Grade ≥2 AEs for the following conditions:
  - Conduction abnormality/atrioventricular heart block
  - Hyperpigmentation ○ Dry skin ○ Photosensitivity ○ Pruritus/itching ○ Rash

- Hair loss/alopecia
- Suicidal ideation or attempt

Note: SAEs or events meeting EAE reporting requirements should also be entered into the DAIDS Adverse Experience Reporting System (DAERS), an Internet-based reporting system.

All AEs that are reported must have their severity graded. To grade AEs, sites must refer to the Division of AIDS Table for Grading the Severity of Adult and Pediatric Adverse Events (DAIDS AE Grading Table), Corrected Version 2.1, July 2017, which can be found on the DAIDS RSC website at https://rsc.niaid.nih.gov/clinical-researchsites/daids-adverse-event-grading-tables.

Serious Adverse Events (SAEs)
A SAE is defined as any untoward medical occurrence that:
- Results in death
- Is life-threatening
- Requires inpatient hospitalization or prolongation of existing hospitalization
- Results in persistent or significant disability/incapacity
- Is a congenital anomaly/birth defect
- Is an important medical event that may not be immediately life-threatening or result in death or hospitalization but may jeopardize the patient or may require intervention to prevent one of the other outcomes listed in the definition above).

7.3 Expedited Adverse Event (EAE) Reporting to DAIDS

7.3.1 Expedited Reporting of Adverse Events to DAIDS

Requirements, definitions and methods for expedited reporting of adverse events (AEs) are outlined in Version 2.0 of the DAIDS EAE Manual, which is available on the RSC website at https://rsc.niaid.nih.gov/clinical-research-sites/manualexpedited-reporting-adverse-events-daids.

The DAIDS Adverse Experience Reporting System (DAERS), an internet-based reporting system, must be used for EAE reporting to DAIDS. In the event of system outages or technical difficulties, EAEs may be submitted using the DAIDS EAE Form. This form is available on the DAIDS RSC website at https://rsc.niaid.nih.gov/clinical-research-sites/paper-eae-reporting.

For questions about DAERS, please contact NIAID CRMS Support at CRMSSupport@niaid.nih.gov. Please note that site queries may also be sent from within the DAERS application itself.

For questions about expedited reporting, please contact the DAIDS RSC Safety Office at (DAIDSRSCSafetyOffice@tech-res.com).

7.3.2 Reporting Requirements for this Study

- The SAE Reporting Category, as defined in Version 2.0 of the DAIDS EAE Manual, will be used for this study from the time of enrollment through week 65 for all participants.

- The study agents for which expedited reporting is required are:
  - Investigational agents:
    - Clofazimine
    - Rifapentine
  - Study-supplied standard TB drugs:
    - Isoniazid
    - Pyrazinamide
    - Ethambutol
    - Rifampicin

7.3.3 Grading Severity of Events

Except for QTcF prolongation (which will be graded according to Table 8.2.1-1), adverse events must be graded according to the Division of AIDS Table for Grading the Severity of Adult and Pediatric Adverse Events (DAIDS AE Grading Table), Corrected Version 2.1, July 2017, available on the DAIDS RSC website at https://rsc.niaid.nih.gov/clinical-research-sites/daids-adverse-event-grading-tables.

7.3.4 Expedited AE Reporting Period

- The expedited AE reporting period for this study is the entire study duration for an individual participant (from study enrollment until study completion or discontinuation of the participant from study participation for any reason).
- After the protocol-defined EAE reporting period, unless otherwise noted, only suspected unexpected serious adverse reactions (SUSARs) as defined in Version 2.0 of the DAIDS EAE Manual will be reported to DAIDS if the study staff become aware of the events on a passive basis (e.g., from publicly available information).

7.4 Study Monitoring

The protocol team will monitor the conduct and safety of the study via regular summaries of **screening failures,** accrual, deaths, SAEs, AEs, study discontinuation, **and delinquency of forms, data, or specimens** pooled across treatment arms. **The A5362 Clinical Management Committee (CMC) will be provided with and monitor QT prolongation, early recurrence, and unfavorable composite outcome in Arm 1 participants.**

The DAIDS clinical representative will review and assess select EAE reports for potential impact on the study participant safety and protocol conduct as per DAIDS policies, guidance documents, and SOPs, as applicable. Additionally, the DAIDS clinical representative will review aggregated AE**s pooled over treatment arms** prepared quarterly by the Statistical and Data Analysis Center (SDAC).

The study will be reviewed by the DSMB at least every 6 months or on another schedule requested by the DSMB. The first interim review will occur 4-6 months after the first enrollment. In addition, there are several triggers for unplanned reviews based on Arm 1 participants with prolonged QTcF interval, recurrences, and unfavorable composite outcomes during the course of the study. The primary outcome of 12-week time to culture conversion will be analyzed as soon as the complete data is available. Depending on the timing of the culture conversion analysis, the DSMB review schedule may need to be adjusted in order to review the final results for the primary outcome and make a recommendation as to whether the study should continue as planned. An ad hoc DSMB review of the data may be requested by the study team or the DAIDS clinical representative if there are serious concerns. See section 10.5 for additional considerations (e.g., triggers) related to interim monitoring.

Detailed plans for study monitoring will be outlined in a Study Progress, Data, and Safety Monitoring Plan developed by the SDMC prior to enrollment of the first participant.

8.0 CLINICAL MANAGEMENT ISSUES

Criteria for participant management, dose interruptions, modifications, and discontinuation of treatment are mandated only for toxicities attributable to studyprovided drugs. The protocol Clinical Management Committee (CMC) must be notified within 48 hours regarding toxicities that result in a change in study treatment (including transitory or permanent discontinuation) during the study-defined treatment period.

8.1 Toxicity

8.1.1 Grade 1 or Grade 2 Toxicity

Participants who develop a Grade 1 or 2 AE or toxicity may continue study drugs without dose adjustment. Participants experiencing Grade 1 or 2 toxicities will be managed at the discretion of the site investigator. Electrolyte abnormalities should be corrected and rechecked.

8.1.2 Grade 3 Toxicity

For any Grade 3 AE or toxicity thought to be secondary to study drugs or of unknown etiology, inclusive of those noted in section 8.2, site investigators must discuss toxicity management with the A5362 CMC. If there is compelling evidence that the AE has NOT been caused by the study drugs, dosing may continue at the discretion of the site investigator/clinician. Except as stated below, participants who develop a Grade 3 AE or toxicity thought to be secondary to study drugs or of unknown etiology will have study drugs withheld. Investigators will discuss toxicity management with the A5362 CMC and study

drugs may be restarted sequentially, depending on the clinical situation; such decisions will be made on a case-by-case basis after consultation with the CMC. The participant should be reevaluated weekly until the AE returns to Grade £2 or until stabilized and clinically no longer in need of weekly monitoring. Participants developing Grade 3 toxicity felt to be due to study drugs that does not resolve within 14 days will be discontinued from the study medications and referred to the National Tuberculosis Program (NTP) for treatment according to local standards of care. They will continue to be followed on-study, off study drugs. In some cases, this will require discontinuation of the co-formulated drugs in place of individual components or alternate anti-TB medications. These cases should be managed individually in conjunction with the A5362 CMC. To the extent possible, such participants will continue to be followed on-study, off study drugs.

8.1.3 Grade 4 Toxicity

For any Grade 4 AE or toxicity thought to be secondary to study drugs or of unknown etiology, inclusive of those noted in section 8.2, site investigators will discuss toxicity management with the A5362 CMC. For a Grade 4 event thought to be secondary to study drugs (decided in conjunction with the A5362 CMC), the participant will be hospitalized and discontinued permanently from study TB medications. The participant will be referred to the NTP for TB treatment according to local standards of care. The participant will continue to be followed on study, off study drugs.

8.2 Specific Conditions

8.2.1 QTcF Prolongation

Participants who receive CFZ (with some also receiving EFV) have the potential of prolongation of the QTcF interval. Grading is based on the average of triplicate ECGs at each visit. Grading is as follows:

Table 8.2.1-1: <u>QTcF Prolongation Grading</u>

Grade 1	Grade 2	Grade 3	Grade 4
(1) Absolute QTcF >480 and ≤500 ms and QTcF change from baseline >0 ms and ≤30 ms; or	(1) Absolute QTcF >480 ms and ≤500 ms and QTcF change from baseline >30 ms and ≤ 60 ms; or	(1) Absolute QTcF >500 ms; or	Life-threatening consequence, e.g., torsades de pointes or other associated serious ventricular dysrhythmia
(2) Absolute QTcF ≤480 ms and QTcF change from baseline >30 and ≤60 ms	(2) Absolute QTcF ≤480 and QTcF change from baseline >60 ms	(2) Absolute QTcF >480 and QTcF change from baseline >60 ms	

If Grade 2 QTc prolongation develops during the course of study treatment, the participant will be monitored more closely, with once weekly ECG testing, and correction of electrolytes where necessary. If Grade 3 QTc prolongation occurs, CFZ will be discontinued and the participant will be hospitalized for monitoring until the abnormality returns to Grade ≤2. Electrolytes will be checked and repleted where necessary. Repeat ECG following repletion of electrolytes will be performed within 3 days, and if QTcF reading is a Grade ≤2, CFZ may be restarted, at the discretion of the investigator. If Grade 3 finding remains, CFZ will be permanently discontinued.

For a Grade 4 event, the participant will be hospitalized and discontinued permanently from study TB medications. The participant will be referred to the NTP for TB treatment according to local standards of care. The participant will continue to be followed on study, off study drugs.

The following events should be reported to the A5362 CMC and to DAIDS immediately:

- Grade 4 QT prolongations
- Deaths
- Results of repeated ECGs after holding CFZ for Grade 3 events

8.2.2 ALT or Total Bilirubin Elevation

Many anti-TB drugs, including the study-provided drugs and components of SOC can cause alterations in liver chemistry tests. Participants entering this trial will have active TB. Elevation in liver function tests is expected. Concomitant illnesses, including HIV infection, and other medications, such as antiretrovirals, may also alter these laboratory parameters. Therefore, changes in liver chemistry tests (ALT, bilirubin) should be evaluated within the clinical context of the abnormalities. Liver chemistry tests will be checked regularly for all study participants, as per section 6.1. All participants who have new Grade ≥3 elevation of ALT should be evaluated for hepatitis B and C virus infection. If there has been documented acute viral hepatitis, the A5362 CMC should be consulted regarding management of study medications.

For participants who develop asymptomatic or symptomatic Grade 3 elevations during study treatment, and, after discussion with the study team), the elevations are not attributed to documented acute viral hepatitis, study-provided drugs and SOC (and ART, if applicable) should be discontinued for up to 2 weeks. Liver function and chemistry tests should be repeated weekly and study-provided drugs and SOC (and ART, if applicable) held until levels and symptoms are Grade ≤2, at which time therapy may be reintroduced. After consultation with the team, if there has been documented acute viral hepatitis and the ALT or total bilirubin takes longer than 14 days to reach Grade ≤2 toxicity, study-provided drugs may be restarted.

If Grade 3 toxicity recurs after re-introduction of study-provided drugs and SOC (and ART, if applicable), or if Grade 3 toxicity does not resolve within 14 days, or if any Grade 4 toxicity develops, the participant will have study-provided drugs permanently discontinued and will be referred to the NTP for TB treatment according to local standards of care. Participants permanently discontinued from ART will be referred to their local HIV clinic or provider for HIV treatment according to local standards of care. The participant will continue to be followed on study, off study-provided drugs.

8.2.3 Allergic Reaction

Participants may continue study drugs for Grade 1 or Grade 2 allergic reactions at the discretion of the study investigator. The participant should be advised to contact the study team immediately if there is any worsening of symptoms or if further systemic signs or symptoms develop. Antihistamines, topical corticosteroids, or antipruritic agents may be prescribed.

Participants with Grade ≥3 allergic reactions that are considered to be possibly or probably related to a study drug should permanently discontinue that study drug. Participants should be treated as clinically appropriate and followed until resolution of the AE. These participants will remain on study until the completion of study follow-up.

8.2.4 Severe Rash/Cutaneous Reaction

Moderate to severe rash potentially related to drug hypersensitivity may occur with any of the study provided TB drugs as well as other drugs participants may be taking.

Participants with a Grade 1 rash may continue study drugs at the study investigator's discretion. Investigators are advised to contact the A5362 CMC immediately if there is any worsening of the rash, if any systemic signs or symptoms worsen, or if mucosal involvement develops. Participants may continue study drugs for an isolated Grade 2 rash. However, study drugs (and all other concurrent medications suspected in the investigator's causality assessment) should be permanently discontinued for any Grade ≥3 rash or for any Grade ≥2 rash that is associated with any of the following:

- Increase in ALT
- Stevens-Johnson syndrome
- Toxic epidermal necrolysis
- Severe hypersensitivity
- Fever, generalized malaise or fatigue
- Muscle or joint aches
- Blisters
- Oral lesions
- Eye inflammation
- Facial swelling

- Swelling of the eyes, lips, mouth
- Breathing difficulty
- Signs and symptoms of liver abnormalities (e.g., jaundice, dark or tea colored urine, pale colored stools/bowel movements, nausea, vomiting, loss of appetite, or right upper quadrant abdominal pain).

If the etiology of the rash can be definitely diagnosed as being unrelated to study drugs and due to a specific medical event or a concomitant non-study medication, routine management should be performed**,** and documentation of the diagnosis provided.

Participants taken off study drug for rash will be referred to the NTP and the local HIV clinic for TB and HIV treatment, according to local standards of care. The participant will continue to be followed on study, off study drugs.

8.2.5 Abdominal Pain, Nausea and/or Vomiting

Although not uncommon, nausea following initiation of therapy with CFZ and/or antiretroviral medications is usually mild and subsides or resolves during treatment. Steps in the management of nausea include taking the medication with food and administration of antiemetic. Antiemetics can only be used if they do not have drug-drug interactions with the study-provided drugs and if they do not have overlapping toxicities with the study-provided drugs.

Participants with Grade ≥3 vomiting or abdominal pain, in particular during the loading dose phase of CFZ, should have their hydration status assessed and be referred to appropriate local medical care for monitoring and volume resuscitation or nasogastric suction, respectively, if clinically indicated depending on sitespecific standard practices. If Grade 3 or Grade 4 abdominal pain, nausea and/or vomiting occurs, if there is compelling evidence that these have NOT been caused by study-provided drugs, dosing may continue and supportive care given. If, after discussion with the A5362 CMC, the site investigator determines that Grade 3 or Grade 4 abdominal pain, nausea and/or vomiting is possibly or probably related to study-provided drugs then study-provided drugs may be temporarily held for up to 14 days until these symptoms return to Grade ≤2. If symptoms do not reach Grade ≤2 within 14 days or if Grade 3 or Grade 4 symptoms occur after reintroduction, then study-provided drugs should be permanently discontinued and the participant referred to his/her national TB program or local TB clinic/provider for treatment of his/her TB according to local SOC. These participants will continue to be followed on study, but off study-provided drugs.

8.2.6 Diarrhea

Diarrhea is a common side effect of infection and medication toxicity. If no infectious cause of diarrhea is found and onset is temporally related to new medication, symptomatic management with antidiarrheal agents is appropriate. Anti-diarrheals (e.g., loperamide) can be used only if they do not have clinically

significant drug-drug interactions with the study drugs and if they do not have overlapping toxicities with study drugs. Participants with diarrhea should have their hydration status assessed daily and given volume resuscitation if clinically indicated depending on local standard practices.

Participants with Grade 3 or Grade 4 diarrhea can have their study medications held for up to 14 days until toxicity resolves to Grade ≤2. If toxicity does not resolve to Grade ≤2 within 14 days, or if Grade 3 or Grade 4 diarrhea occurs after reintroduction, the participant will be discontinued from study medications and referred to the NTP for TB treatment and to the local HIV clinic for HIV treatment according to local standards of care. The participant will continue to be followed on study, off study drugs.

8.2.7 Peripheral Neuropathy

Isoniazid has been associated with the development of peripheral neuropathy. If a participant develops new or worsening Grade 1 or Grade 2 peripheral neuropathy during the course of the study, the participant will continue on isoniazid, undergo investigation for treatable causes of the impairment (e.g., glucose, vitamin B12 levels), be treated symptomatically according to local standards, and given pyridoxine 100 mg daily. If a participant develops new or worsening Grade 3 or Grade 4 peripheral neuropathy, isoniazid will be temporarily discontinued and the participant will be provided pyridoxine 100 mg daily. If symptoms do not stabilize or improve after 14 days, isoniazid will not be restarted, and the participant will be discontinued from study medications and referred to the NTP for TB treatment according to local standards of care and followed on study, off study drugs.

8.2.8 Arthritis/Arthralgia

Arthritis and arthralgia are common symptoms experienced by participants on pyrazinamide.

If a participant develops Grade ≤3 arthritis or arthralgia during the course of the study, the participant will continue on study drugs and be treated according to local standard practices. If the participant develops Grade 4 arthritis or arthralgia, pyrazinamide will be held for up to 14 days until toxicity resolves to Grade ≤2. If toxicity does not resolve to Grade ≤2 within 14 days, the participant will be discontinued from study medications and referred to the NTP for TB treatment according to local standards of care and followed on study, off study drugs. If toxicity resolves to Grade <2 within 14 days but Grade 4 arthritis or arthralgia occur after reintroduction of pyrazinamide, pyrazinamide will be permanently stopped.

8.2.9 Depression

Participants living with HIV may occasionally present with symptoms of depression and/or suicidality (suicidal ideation or behavior). In addition, there have been reports of depression, suicidal ideation and behavior (particularly in

participants with a pre-existing history of depression or psychiatric illness) in participants treated with CFZ or integrase inhibitors. Therefore, it is appropriate to monitor participants for depression before and during treatment.

If depression occurs, participants should be monitored and referred appropriately for unusual changes in behavior. It is recommended that the investigator consider mental health consultation or referral for participants who experience moderate (score 16-19 on CES-D) or severe (score >20 on CES-D) depression. In the case of severe depression, the investigator should arrange for escort of the participant to mental health services.

If Grade 3 (severe mood alteration interfering with activities of daily living (CES-D score ^{3}16) or Grade 4 (suicidal ideation; danger to self or others) occur, and if there is compelling evidence that these have NOT been caused by study-provided drugs, dosing may continue, and supportive care given. If, after discussion with the study team, the site investigator determines that Grade 3 or Grade 4 mood alteration is possibly or probably related to study-provided drugs then study-provided drugs will be temporarily held for up to 14 days until symptoms return to Grade ≤2. If symptoms do not reach Grade ≤2 within 14 days or if Grade 3 or Grade 4 symptoms occur after reintroduction, then study-provided drugs should be permanently discontinued, and the participant referred to his/her national TB program or local TB clinic/provider for treatment of his/her TB according to local SOC. These participants will continue to be followed on study, but off study-provided drugs.

If the participant expresses suicidal ideations or intents, the data will be captured as an AE. Any suicidal thought or suicide attempt that qualifies as an EAE will be reported using the standard EAE mechanism.

8.3 Pregnancy

Pregnancy and pregnancy outcomes that occur on study among females taking ART will be recorded on the eCRFs. Pregnancies that occur on study should be reported prospectively to the Antiretroviral Pregnancy Registry. More information is available at www.apregistry.com. Telephone: 910-679-1598; Fax: 44-1628-789-666 or 910-256-0637.)

<u>Pregnancy Outcomes and Reporting</u>
If a participant becomes pregnant during the study, she will be discontinued from study medications and referred to the NTP for the treatment of TB according to local standards of care, and to a prenatal care program for management of her pregnancy according to local standards of care. Women living with HIV will be referred to their local HIV clinic for appropriate care. Women will be followed through the end of the study period. At the end of the pregnancy, the outcome and AEs for the participant and the infant will be recorded on the outcome eCRFs.

If a female participant has completed the study or chooses to discontinue from the study before the end of the pregnancy, site staff should request permission to contact her regarding pregnancy outcomes at the end of pregnancy. If the information is obtained, pregnancy outcomes will be submitted on the eCRFs at the end of the pregnancy.

9.0 CRITERIA FOR DISCONTINUATION

9.1 Permanent and Premature Treatment Discontinuation

- Serious drug-related toxicity (see sections 8.1 and 8.2).
- Requirement for prohibited concomitant medications (see section 5.4).
- Completion of treatment as defined in the protocol.
- Participants later found to have isolates with INH **or RIF** resistance.
- Participants whose screening, entry, and first post-entry visit sputum cultures all fail to grow *Mtb* complex.
- Pregnancy or breastfeeding.
- Request by participant to terminate treatment.
- Clinical reasons believed life threatening by the physician, even if not addressed in the toxicity section of the protocol.

9.2 Premature Study Discontinuation

- Request by the participant to withdraw.
- Request of the primary care provider if he or she thinks the study is no longer in the best interest of the participant.
- At the discretion of the ACTG, IRB/EC, FDA, NIAID, Office for Human Research Protections (OHRP), and other US, local, and international regulatory entities as part of their duties to ensure that research participants are protected, or the industry supporter or its designee.

10.0 STATISTICAL CONSIDERATIONS

10.1 General Design Issues

This is an open-label, randomized phase IIc study to evaluate whether a 3-month regimen of PHZE + 100 mg CFZ with a 2-week loading dose is superior to the standard 6-month regimen of RHZE for DS-TB with respect to early bacteriologic efficacy defined as time to culture conversion by 12 weeks post-randomization. Additionally, participants will be followed for up to 65 weeks in order to characterize and compare safety, relapse rates, and a composite long-term favorable outcome 1 year post-randomization as key secondary outcomes.

10.2 Outcome Measures

Primary and secondary outcome measures below will be addressed in the study's primary Statistical Analysis Plan, which will define the content of the Primary Analysis Report. This report will form the basis for the primary study manuscript and results reporting to ClinicalTrials.gov. Outcomes of interest for secondary and exploratory objectives intended for subsequent publication are listed under "Exploratory Outcome Measures."

10.2.1 Primary Outcomes

10.2.1.1 Time to stable culture conversion in liquid media, defined as the first of two (consecutive or non-consecutive) negative sputum cultures without an intervening positive culture, and/or visits wherein the participant is unable to produce sputum and has no signs of active TB, up to 12 weeks post-randomization. **[Objective 1.2.1.1]**

10.2.1.2 Safety: proportion across study arms experiencing any Grade 3 or higher AE that is at least a one grade increase from baseline over 65 weeks. **[Objective 1.2.1.2]**

10.2.2 Secondary Outcomes

10.2.2.1 A. Efficacy: proportion with favorable clinical/bacteriologic outcome (definition 10.2.4.A) at 65 weeks post-randomization. **[Objective 1.3.8]**

B. Efficacy: proportion with favorable composite outcome including treatment completion (definition 10.2.4.B) at 65 weeks postrandomization.

C. Efficacy: proportion with favorable composite outcome including treatment completion (definition 10.2.4.B) at 65 weeks postrandomization. **[Objective 1.3.1]**

10.2.2.2 Tolerability: proportion **with** premature treatment **discontinuation through 65 weeks**, defined as discontinuation other than due to violent death, natural disaster, or administrative censoring. **[Objective 1.3.2]**

10.2.2.3 A. Mean baseline QTcF and change from baseline in mean QTcF at weeks 2, 8, and end of treatment (EOT) **(Arm 1, week 13; Arm 2, week 26)**.

B. Occurrence of absolute QTcF ≥480 ms and ≤500 ms, and ≥500 ms, at any time during study treatment.

C. Occurrence of QTcF change from baseline of ≥30 ms and ≤60 ms, and ≥60 ms, at any time during study treatment. **[Objectives 1.3.3 and 1.4.1.1]**

10.2.2.4 Time to stable culture conversion in **liquid and** solid media **through week 65** defined as the first of two (consecutive or non-consecutive) negative sputum cultures without an intervening positive culture, and/or visits wherein the participant is unable to produce sputum and has no signs of active TB. **[Objectives 1.3.4, 1.4.1.2, and 1.4.7.2]**

10.2.2.5 Proportion of participants **achieving** culture conversion at weeks 8 and 12 (**liquid** MGIT and solid media considered separately). **[Objective 1.3.5]**

10.2.2.6 Time (days) to positivity in liquid culture (MGIT) after start of treatment through week 65. [Objectives 1.3.6 and 1.4.1.3]

10.2.2.7 Change in chest X-ray score from baseline to EOT. [Objective 1.3.7]

10.2.2.8 Proportion of participants with one or more SAEs **through week 65. [Objective 1.2.1.2]**

10.2.2.9 Proportion of participants who have a TB relapse, from EOT until week 65. **[Objective 1.3.9]**

10.2.2.10 Proportion of participants who have a TB recurrence, from EOT until week 65. **[Objective 1.3.9]**

10.2.2.11 Plasma pharmacokinetic parameters for CFZ, estimated using noncompartmental methods applied to concentrations from intensive PK sampling visits at weeks 2 **(Arm 1 and Arm C)** and 13 **(Arm 1).** Minimum concentration (C_{min}), maximum concentration (C_{max}), time of C_{max} (T_{max}), and area under the concentration curve ($AUC_{0\text{-}24h}$). Weeks 2 and 13 PK parameters will be summarized separately. **[Objectives 1.3.10 and, 1.3.11]**

10.2.2.12 Change from baseline in skin pigmentation (colorimetric L*, a*, b* parameters) utilizing ambient light-independent reflectance mapping at weeks 8, 13, 26, and 65. [Objective 1.3.12]

10.2.2.13 Change from baseline in participant-reported changes in skin pigment and distress related to perceived skin hyperpigmentation at weeks 8, 13, 26, and 65. [Objective 1.3.12]

10.2.3 Exploratory Outcomes

10.2.3.1 CFZ-associated adverse events through week 65. [Objective 1.4.1.4]

10.2.3.2 Acquired drug resistance to CFZ through week 65. [Objective 1.4.1.5]

10.2.3.3 Plasma pharmacokinetic parameters for rifapentine (RPT), estimated using noncompartmental methods applied to concentrations from intensive PK sampling visits for Arms 1 (Week 2 and Week 13) and C (Week 2) (C_{min}, C_{max}, T_{max}, $AUC_{0\text{-}24h}$). [Objective 1.4.2]

10.2.3.4 Qualitative interview at EOT (Arm 1, week 13; Arm 2, week 26) to assess participant experiences with social stigma and impact of perceived skin changes on quality of life. [Objective 1.4.4]

10.2.3.**5** Change from baseline in participant-reported quality of life as measured by the WHOQOL-BREF at weeks 8, 13, 26, and 65. **[Objectives 1.4.1 and 1.4.5]**

10.2.3.**6** Change from baseline in CES-D score at weeks 8, 13, 26, and 65. **[Objective 1.4.6]**

10.2.3.7 Sputum RS ratio at weeks 1, 2, 3, 4 (Arm 1 and Arm C) or weeks 2 and 4 (Arm 2) and at time of Possible Poor Treatment Response (Arm 1, Arm 2, and Arm C). [Objectives 1.4.7.1, 1.4.7.2, 1.4.7.3, 1.4.7.4, and 1.4.7.5]

10.2.3.**8** Population PK model parameters of CFZ **through week 65. [Objectives 1.3.10, 1.4.1.1, 1.4.1.2, 1.4.1.3, 1.4.1.4, and 1.4.1.5]**

10.2.4 Definitions of Secondary Composite Outcome Status

For the key secondary analysis of favorable composite outcome, each participant will be classified into one of three outcome categories: Favorable, Unfavorable, or Unevaluable. If unevaluable, the participant will be censored at the time of their last visit from efficacy analyses.

A. Clinical/bacteriologic efficacy outcome measure (refers to 10.2.2.1A)

Favorable Outcome (any one of the following scenarios) •
Participants with liquid culture negative status at week 65.

- Participants without signs or symptoms of ongoing active TB and are unable to produce a sputum specimen at 65 weeks.
- Participants who at the end of the follow-up period are clinically without symptoms/signs of ongoing active TB and produce a sputum specimen that is contaminated in two liquid cultures without evidence of TB.

For any of the above to be considered favorable, participants must not already have been considered unfavorable, as defined below.

Unfavorable Outcome

- Absence of cure. A participant will be considered to have absence of bacteriological cure if a sputum sample obtained at or after end of treatment **for Arm 1** (week 13), or at or after week 17 for Arm 2 is culture-positive in liquid or solid media for an *Mtb* strain genotypically matched with the initial isolate. A second sputum sample, obtained at least four hours following the first sputum collection (see section 6.2.3), is required to confirm absence of bacteriological cure.
- Participants who die from any cause during study treatment or follow-up except from violent or accidental cause (e.g., road traffic accident).

- Participants who had a positive culture for *Mtb* when last seen, whether confirmed by a second sample or not, unless determined to have been reinfected.
- Participants experiencing extension of treatment beyond the nominal level (week 13 for Arm 1 and week 26 for Arm 2) due to clinically inadequate response. These cases should be managed individually in conjunction with and after having notified the A5362 CMC. Extension of treatment to make up missed doses will not count as unfavorable.

<u>Unevaluable Outcome</u>

- Participants lost to follow-up during treatment or post-treatment follow-up with their last culture being negative for *Mtb*.
- Violent or accidental death.
- Participants with recurrent TB due to a new strain of *Mtb* confirmed by conventional molecular genotyping (as defined by MIRU and IS6110 typing).
- Women who become pregnant during their assigned active treatment and stop their assigned treatment.

B. Composite efficacy outcome measure (refers to 10.2.2.1B)

<u>Favorable Outcome</u>

Same as in Outcome Measure 10.2.2.1A.

<u>Unfavorable Outcome</u>

Same as in Outcome Measure 10.2.2.1A, and:

- Participants lost to follow-up during treatment phase of each Arm (i.e., 13 weeks and 26 weeks for Arms 1 and 2, respectively).
- Participants failing to complete treatment (see sections 10.2.5 and 10.2.6, Definitions of Adequate Treatment) and not assessable at the end of the follow-up period.
- Participants receiving any one or more of the following:
    - Extension of treatment beyond the nominal level, except to make up missed doses
    - A re-start of treatment following ≥30 consecutive days lost to follow-up ○ A change in at least one drug in treatment regimen for any reason except re-infection, pregnancy, or temporary drug challenge.

<u>Unevaluable Outcome</u>

- Participants lost to follow-up after treatment phase, and not assessable at the end of follow-up, with their last culture being negative for *Mtb.*
- Violent or accidental death.
- Participants re-infected with a new strain of *Mtb* confirmed by conventional molecular genotyping (as defined by MIRU and IS6110 typing).
- Women who become pregnant during their assigned active treatment and stop their assigned treatment.

10.2.5 Definitions of Adequate Treatment Within 3-month Regimen (Arm 1)

- Taken a total of at least 56 doses of allocated intensive phase treatment within 70 days of starting treatment
- AND taken at least 28 doses of continuation phase treatment within 49 days (7 weeks) of completion of the intensive phase
- AND missed no more than 21 doses of medication overall.

10.2.6 Definitions of Adequate Treatment within 6-month Regimen (Arm 2)

- Taken a total of at least 56 doses of allocated intensive phase treatment within 70 days of starting treatment
- AND taken at least 100 doses of continuation phase treatment within 154 days (22 weeks) of completion of the intensive phase
- AND missed no more than 42 doses of medication overall.

10.3 Randomization and Stratification

If willing to consent to random assignment in Arm C, participants will be randomized in a 2:1:1 ratio between Arms 1, 2, or C, using permuted blocks, balanced by institution. If unwilling to consent to random assignment in Arm C, participants will be randomized in a 2:1 ratio between Arms 1 or 2 only, using permuted blocks, balanced by institution. Participants will be stratified based on HIV status, and by presence of advanced disease on chest X-ray (see section 6.3.11) conducted at screening.

The first 20 participants randomized to Arm 1 who also consent to intensive PK sampling will be registered to the Intensive PK Sampling subgroup. Participants who withdraw consent for participation in the Intensive PK sampling in Arm 1 or Arm C will be replaced. In both Arm 1 and Arm C, accrual will be limited to 6 participants per site with intensive PK sampling. If fewer than 4 sites are able to carry out intensive PK sampling then the accrual limit per site will be increased in order to reach the target sample size of 20.

The first 20 participants randomized to each of Arm 1 and Arm 2 who also consent to answering an open-ended qualitative interview at the end of treatment will be registered to the Qualitative Interview subgroup. We will use accrual limits to balance enrollment to the Qualitative Interview subgroup by site and by treatment arm. Participants who withdraw consent for participation in the Qualitative Interview subgroup will be replaced.

NOTE: Participants in Arm 1 may consent to both intensive PK sampling and qualitative interview, one, or neither.

Consent into Arm C predicated on participant willingness to undergo intensive PK studies, potentially indicating a relatively “healthier” group, raises the possibility of selection bias for a comparison of safety rates between Arm C (CFZ no loading dose) with Arm 1 (CFZ loading dose). However, (1) PK concentrations are the primary comparison of interest between these arms, and these should be unbiased (since the comparative groups are both willing to undergo intensive PK studies by nature of their inclusion); (2) we note the expectation that better safety will occur with Arm C vs. Arm 1,

given the lack of loading dose; and (3) alternative randomization schemes, such as requiring ALL recruited participants to be willing to undergo intensive PK as a condition of enrollment, risk considerable correlate problems with respect to external validity.

10.4 Sample Size and Accrual

Primary Efficacy Outcome

For the primary efficacy outcome, we plan to compare time to liquid culture conversion between Arm 1 (CFZ load) and Arm 2 (SOC) up to 12 weeks, a common surrogate endpoint for Phase II TB trials. For a treatment-shortening regimen to work effectively, it is reasonable to assume that it is necessary for culture conversion rates in the experimental arms to be faster than in SOC. Under a proportional hazards model, effect sizes that are inversely proportional to length of treatment would correspond to a necessary hazard ratio of 2.0 for the 3-month arm. The high-dose rifampicin trial conducted by the PanACEA [**107**] consortium assumed a true hazard ratio (HR) of 1.8 would indicate potential for treatment shortening since it represented an increase over the HR 1.68 seen in the Phase II trial that led to the Phase III REMox trial (which was ultimately unable to demonstrate non-inferiority). We also propose that a true HR of at least 1.8 would be necessary for the 3-month arm to have potential as a shortened regimen comparable to SOC.

Under a 70% 12-week culture conversion event proportion in the control arm (Table 10.4-1), a true hazard ratio of 1.8 for Arm 1 vs. Arm 2, a one-sided alpha level of 0.05, and a 2:1 randomization allocation to Arm 1: Arm 2, we would need a total of 150 evaluable participants to have 90% power to reject the null hypothesis that there is no difference in time to 12-week culture conversion between the experimental and control arms. Assuming approximately 10% are late exclusions, withdraw, or have several missing culture results, we propose to enroll 165 participants for efficacy analyses (110 Arm 1, 55 Arm 2).

Table 10.4-1: Comparative Historical SOC Efficacy Rates

Trial	12-week MGIT Culture Conversion in Control Arms (Primary Outcome)	52-week Bacteriologic/ Clinical Efficacy (Secondary Outcome; 10.2.2.1A)	52-week Composite Treatment Outcome (Secondary Outcome; 10.2.2.1B) (mITT)	52-week Composite Treatment Outcome (Secondary Outcome; 10.2.2.1B) (Per Protocol)
REMox [**108**]	65-70% (12-week)	89%	89%	91%
OFLOTUB [**109**]	-- [2]	88.4% [4]	82.8% (104 weeks) [1]; N=694	88.7% [4]

RIFAQUIN [**110**]	81% (8-week)[3]	91.2%[4]	85.6% (78 weeks);[4] N=188	95.1%[4]
Trial	12-week MGIT Culture Conversion in Control Arms (Primary Outcome)	52-week Bacteriologic/ Clinical Efficacy (Secondary Outcome; 10.2.2.1A)	52-week Composite Treatment Outcome (Secondary Outcome; 10.2.2.1B) (mITT)	52-week Composite Treatment Outcome (Secondary Outcome; 10.2.2.1B) (Per Protocol)
PanACEA [**107**]	70.1% (12-week)	--	--	--
Adopted A5362 parameters	70%	88-92%	84-90%	90-94%

Please see section 10.2.4 for definition of efficacy outcomes.

[1] 24 months from end of treatment; other values are from time of randomization

[2] Performed only on solid culture

[3] MGIT only performed at certain sites and only at week 8

[4] Only 76-week outcome available

Primary Safety Outcome

For the primary safety outcome, we will compare proportions of participants with any Grade 3 or higher AE between arms. Table 10.4-2 gives the power we would have to detect that Arm 1 is inferior to Arm 2 assuming a two-sided type 1 error of 0.10, 10% probability of developing an AE in the SOC arm, and varying probabilities (15-30%) of developing an AE in experimental arm. The 90% confidence interval width is also provided for the comparison.

Table 10.4-2: Primary Safety Outcome Comparison

Power	N1	N2	N	Arm 1 Proportion with Grade ≥3 AE	Arm 2 Proportion with Grade ≥3 AE Arm 2	Two-sided type 1 error	90% CI Width
0.13	100	50	150	0.15	0.10	0.10	0.210
0.36	100	50	150	0.20	0.10	0.10	0.218
0.64	100	50	150	0.25	0.10	0.10	0.225
0.85	100	50	150	0.30	0.10	0.10	0.230

Secondary Efficacy Outcome

For the secondary clinical/bacterial efficacy outcome (10.2.2.1A), we assume a favorable proportion of 88-92% in the control arm by week 65 (Col 2, Table 10.4-3). This estimate is based on the mITT control arms of the RIFAQUIN and REMox trials. Therefore, we assume a favorable proportion of 84-92% in the control arm by week 65 for efficacy outcome 10.2.2.1B.

For secondary outcome 10.2.2.1 (proportion with favorable composite outcome at 65 weeks), using an unpooled Z-test with one-sided alpha of 0.05 and sample sizes of 100 in Arm 1 and 50 in Arm 2, the power for a range of non-inferiority margins and favorable outcome proportions from 84% to 92% is given in Table 10.4-3. Note that a margin of 67% (with a one-sided alpha of 0.025) is commonly used in Phase III drug-sensitive TB treatment-shortening trials [**107-110**], but we would have little power on this secondary endpoint to show non-inferiority based on that margin.

Table 10.4-3: Composite Secondary Efficacy Outcome Power

Favorable proportion in each arm	NI Margin	Power (%)
0.84	0.08	35.0
0.84	0.10	47.2
0.84	0.12	59.7
0.88	0.08	41.2
0.88	0.10	55.2
0.88	0.12	68.7
0.92	0.08	52.3
0.92	0.10	68.6
0.92	0.12	81.8

For this reason, we will not be making a formal comparison with a pre-specified margin, and will solely provide the 95% confidence interval for the difference in proportions between arms. If observed sample proportions in both arms were 0.84, 0.88, or 0.92, the 95% one-sided lower confidence bound for the difference in proportions between arms would be -0.106, -0.094, or -0.080, respectively, using a Score confidence interval with continuity correction.

<u>Accrual</u>
The accrual rate is expected to be 30-40 participants per month, so the study is expected to fully accrue within approximately 6 months.

<u>Sample Size for PK Study</u>
Table 10.4-4 provides a summary of the expected margin of error (ME) under different levels of variability (quantified as coefficient of variance, CV%) and sample sizes. The sample size was selected to give sufficient precision in the estimation of geometric mean AUC_{0-24h} for both CFZ and P. To account for a worst-case scenario, the highest identified and reported CV% was used which in this case is up to 83% variability in CFZ AUC reported by Nix et al. The variability in P is expected to be much lower, ~40 CV% according to Savic et al. Twenty patients with rich PK sampling is expected to be sufficient to get a margin of error of ~20% for the geometric mean of AUC_{0-24h} for both CFZ and P. **There will be 20 participants randomized to Arm C, the intensive PK sampling arm. Additionally, the first 20 participants on Arm 1 who consent to intensive PK sampling will be registered to the Intensive PK Sampling subgroup.**

Table 10.4-4: Margin of Error (ME) for PK Parameter Estimates Given Different Expected Levels of Variability (CV%) and Sample Size (N)

CV%	30	50	80	100
N	ME	ME	ME	ME
10	0.136	0.219	0.327	0.387
15	0.106	0.171	0.255	0.312
20	0.090	0.145	0.216	0.256
25	0.080	0.128	0.191	0.226
30	0.072	0.116	0.173	0.205
60	0.050	0.080	0.120	0.142

Sample Size for Qualitative **Interview Subgroup**

For the qualitative study assessing social stigma related to skin color changes, we plan to sample a total of 40 participants (20 participants in Arm 1 and 20 participants in Arm 2), balanced by site and treatment arm. **The first 20 participants in each of Arm 1 and Arm 2 who consent to participation in the open-ended qualitative interview will be registered to the Qualitative Interview subgroup.**

10.5 Data and Safety **Event** Monitoring

Section 7.4 describes study monitoring, including details of reviews by the DSMB. In this section, the statistical considerations of stopping guidelines and interim analysis are described.

For all reviews, the DSMB will be provided detailed information on safety, tolerability, and administrative aspects (including accrual, retention, and compliance with study requirements).

10.5.1 Interim Monitoring Guidelines

Early Assessment of QT Prolongation **in Arm 1**

After we enroll the first 12 participants to Arm 1 and have evaluable AE data through week 2, we will assess the proportion of participants with prolonged QT interval defined by QTcF >500 ms**.** If the upper bound of the two-sided PearsonKlopper exact 95% confidence interval (CI) around the proportion of Arm 1 participants exhibiting this AE (QTcF >500 ms) exceeds a threshold of 40%, the team will evaluate the events carefully in conjunction with the DAIDS Clinical Representatives and the DSMB to consider whether there should be a reduction in the CFZ loading dose to 200 mg daily for 2 weeks. Based on 12 participants having data, if two or more participants experience the AE**,** this evaluation will be prompted (i.e., the upper confidence limit would be >40%). Otherwise, if one or no participants experience the AE, the CFZ daily loading dose will remain at 300 mg.

Ideal characteristics of the trigger for DAIDS Clinical Representatives and DSMB evaluation are that the threshold is: (1) unlikely to be exceeded for low probabilities of the targeted AE (e.g., less than 5%) and (2) likely to be exceeded for high probabilities of the AE (e.g., 20% and above). Table 10.5-1 shows the probabilities of a trigger occurring for a range of considered scenarios defined by the number of evaluated participants and several threshold values for a range of true AE rates. The team selected to evaluate the first 12 participants using a threshold of 40% because of its good operating characteristics. Specifically, if the true probability of the targeted AE is 5%, there is a low probability (11%) of triggering evaluation. Conversely, if the true AE probability is 30%, there is a high probability (91%) of triggering evaluation.

Table 10.5-1: Probability that Upper Bound of an Exact Pearson-Klopper 95% Confidence Interval for the Probability of Adverse Event (AE, QTcF >500 ms) Is Above Several Thresholds for Selected Number of Observed Participants and True Rates of AE

Number of Participants Evaluated	True probability of QTcF 500 ms	Threshold		
		0.3	0.35	0.4
8	0.01	1.0	1.0	0.08
	0.05	1.0	1.0	0.34
	0.10	1.0	1.0	0.58
	0.20	1.0	1.0	0.83
	0.30	1.0	1.0	0.94
	0.50	1.0	1.0	1.0
12	0.01	0.11	0.12	0.01
	0.05	0.47	0.46	0.11
	0.10	0.71	0.72	0.34
	0.20	0.93	0.93	0.72
	0.30	0.99	0.99	0.91
	0.50	1.0	1.0	1.0
15	0.01	0.14	0.01	0.01
	0.05	0.55	0.17	0.17
	0.10	0.79	0.44	0.45
	0.20	0.97	0.82	0.83
	0.30	1.0	0.97	0.97
	0.50	1.0	1.0	1.0

Interim Futility Analysis

There will be one interim futility analysis for the primary efficacy endpoint **once 50% of participants have reached week 12 of follow-up**. The conditional power given the data available will be computed, and the DSMB could consider using a conditional power <0.2 as one criterion among many for early stoppage.

Unfavorable Composite Outcome Monitoring **in Arm 1**
Monitoring for unfavorable **composite** outcome will begin **approximately 1 month** after the first participant completes treatment in **Arm 1 and will continue approximately monthly and for each DSMB review until the end of the study**. Two-sided 95% confidence bands for the cumulative probability of unfavorable outcome over time will be generated. If the lower confidence band for the cumulative probability exceeds 12% at any time, this will trigger a DSMB review.

Early **Recurrence** Monitoring **in Arm 1**
TB **recurrence** monitoring will begin **approximately 2 months** after the first participant completes treatment in **Arm 1 and will continue approximately monthly and for each DSMB review until the end of the study**. Two-sided 95% confidence bands for the cumulative probability of **recurrence** over time will be generated. If the lower confidence band for the cumulative probability exceeds 3% at any time, this will trigger a DSMB review.

10.5.**2** Interim Analysis Plan

Conditional Power **for the Futility Analysis**
The conditional power of the test statistic will be estimated **once 50% of participants have reached week 12 of follow-**up using two methods. The first method will be under the assumption that the future data follows the hypothesized difference between Arm 1 and Arm 2 (hazard ratio of 1.8 between arms). The second method will use the assumption that future data follows the estimated difference between Arm 1 and Arm 2 at the time of the review (the estimated hazard ratio).

Confidence Bands for Unfavorable **Composite** Outcome Probabilities **The unfavorable composite outcome will only include treatment failures, treatment extensions, loss to follow-up while culture positive, recurrences, and deaths.** Two-sided 95% confidence bands will be generated for **the** cumulative unfavorable **composite** outcome probabilit**y** in **Arm 1**. **Since Arm 2 data will be lagging 3 months behind Arm 1, t**he will be presented alongside historical unfavorable proportions for SOC on the REMox, OFLOTUB, and RIFAQUIN trials, and A5362 SOC data as **it becomes** available. There will be no adjustment to the confidence bands for multiple looks.

Confidence Bands for Early **Recurrence** Probabilities
Recurrence captures both relapses and reinfections. Two-sided 95% confidence bands will be generated for **the** cumulative **recurrence** probabilit**y** in **Arm 1**. **Since Arm 2 data will be lagging 3 months behind Arm 1, t**he **recurrence data** will be presented alongside historical re**currence** proportions for SOC on the REMox, OFLOTUB, and RIFAQUIN trials, and A5362 SOC data as **it becomes** available. There will be no adjustment to the confidence bands for multiple looks.

10.6 Analysis Sets

- **Efficacy set:**
  All participants who are randomized to study treatment and are not late exclusions. (Participants will be late exclusions if they do not have any positive culture at screening, entry, or week 1, or if they have TB that is RIF resistant or INH resistant.)

- **Safety set:**
  All participants who start assigned study treatment.

- **Start treatment/not late exclusion set:**
  All participants who start assigned study treatment and are not late exclusions.

- **Alive/adequate treatment set:**
  All participants who complete an adequate course of treatment as defined within sections 10.2.5 and 10.2.6 of the protocol and who do not die during treatment.

- **Per protocol set:**
  All participants who start and complete assigned study treatment.

10.7 Analyses

A separate Statistical Analysis Plan document describes the analysis of each outcome measure in detail.

Primary Outcome Measures

Analysis of Outcome Measure 10.2.1.1

The hazard ratio and 95% one-sided lower confidence interval for time to stable culture conversion in liquid media up to 12 weeks will be estimated using a Cox proportional hazards model **in the efficacy set,** comparing **Arm 1** with **Arm 2**. The model will be adjusted for, at a minimum, stratification variables (HIV status, and radiographically advanced disease status), and site. Participants lost to follow-up prior to week 12 will be censored, and all participants who do not achieve culture conversion will be censored at week 12. If the lower confidence bound is greater than 0 (i.e., superiority/faster time to culture-conversion is demonstrated), we would conclude that the experimental arm has superior time to culture conversion and adequate clinical benefit to be studied further.

Hazard ratios and confidence intervals will also be reported within subgroups of **natal** sex and race/ethnicity, and we will formally test if hazard ratios are independent of **natal** sex and race/ethnicity using interaction terms.

As noted earlier, this analysis will be completed prior to the end of the study, and results will be provided to the DSMB as soon as they are available.

Analysis of Outcome Measure 10.2.1.2

We will estimate the proportion of participants with any AE Grade 3 or higher during follow-up that is at least one grade increase from baseline in the safety set comparing Arm 1 with Arm 2. Proportions will be estimated using the Kaplan-Meier estimator, with participants who are lost to follow-up censored at the time of their last follow-up visit.

Secondary **Outcome Measures**

Analysis of Outcome Measures 10.2.2.1A and 10.2.2.1B

For the composite efficacy outcomes 10.2.2.1 A and B, we will calculate the 90% and 95% two-sided confidence intervals for the difference in **cumulative** proportion having a favorable outcome by the final follow-up visit (week 65) between **Arm 1 and Arm 2**. The analysis **for 10.2.2.1A** will be carried out in **the safety set**, defined as all randomized participants who started their assigned study treatment. Participants who never started study treatment in either arm will be excluded from the analysis because including these participants would make the arms more similar and bias the comparison toward noninferiority. The favorable outcome proportion at week 65 will be estimated using a Kaplan-Meier **estimator**, with Greenwood's estimate for the standard error. For outcome 10.2.2.1A, participants who are re-infected, are late screening failures, or are lost to follow-up and culture negative at their last visit, will be censored at the time of their last follow-up. For the secondary composite outcome measure 10.2.2.1B, **the analysis will be carried out in the per protocol set, defined as all participants who start and complete assigned study treatment. T**he same censoring criteria apply.

Analysis of Outcome Measure 10.2.2.2

We will estimate the difference in proportions of participants who permanently and prematurely discontinue study treatment between Arm 1 and Arm 2 using binomial proportions. We will report the exact 95% confidence interval for the difference in proportions. We will carry out this analysis in the start treatment/not late exclusion set.

Analysis of Outcome Measure 10.2.2.3

Change from baseline in QTcF over time will be compared between Arm 1 and Arm 2 using a linear mixed-effects model. We will carry out this analysis in the safety set. Proportions of participants in each arm with maximum observed QTcF above 500 ms, between 480 and 500 ms, and 480 ms or less will be compared between arms using multinomial logistic regression; analogously for proportions of participants with observed change in QTcF from baseline above 60 ms, between 30 and 60 ms, and 30 ms or less.

Analysis of Outcome Measure 10.2.2.4

Time to stable culture conversion at week 65 will be assessed separately in liquid and solid media in each CFZ arm against the control arm using log-rank tests, and hazard ratios with two-sided 95% confidence intervals will be provided. Time to stable culture conversion within each study arm will be described graphically using Kaplan-Meier estimates. We will carry out this analysis in the efficacy set.

Analysis of Outcome Measure 10.2.2.5
We will estimate the proportion with culture conversion separately for liquid and solid media at weeks 8 and 12 in Arm 1 and Arm 2 with the Kaplan-Meier estimator. We will carry out the analysis in the efficacy set.

Analysis of Outcome Measure 10.2.2.6
Time to positivity will be **compared separately at weeks 1, 2, 3, 4, 6, 8, 10, and 12 using Cox proportional hazards models or log-rank tests. We will carry out this analysis in the efficacy set.**

Analysis of Outcome Measure 10.2.2.7
The Chest X-ray Score (CXR Score) will be computed based on the chest x-rays conducted during screening and at the end of treatment using the following validated formula [106]: CXR Score = percent of total lung affected by any pathology + 40 (if cavitation is present). We will compute the CXR scores based on both site assessment of the chest x-ray and an assessment by independent central reviewers blinded to study treatment. We will compare the mean change in CXR score between Arms 1 and 2 from baseline to end of treatment using t-tests. We will carry out this analysis in the efficacy set.

Analysis of Outcome Measure 10.2.2.8
We will estimate the proportion of participants in Arm 1 and Arm 2 with one or more SAEs using the Kaplan-Meier estimator. We will carry out this analysis in the safety set.

Analysis of Outcome Measure 10.2.2.9
The proportion of participants who experience relapses **in Arm 1 and Arm 2** will be estimated using the Kaplan-Meier **estimator**. **We will carry out this analysis in the start treatment/not late exclusion set.**

Analysis of Outcome Measure 10.2.2.10
The proportion of participants who experience recurrence will be estimated similarly as above.

Analysis of Outcome Measure 10.2.2.11
A population PK model will be developed based on the intensive and sparse PK sampling. **The PK parameters for CFZ in Arm 1 and Arm C will be estimated using non-compartmental methods applied to concentrations from intensive PK sampling visits. We will estimate the geometric mean ratio for each PK parameter together with the 90% confidence interval using available data in the alive/adequate treatment set.**

Analysis of Outcome Measure 10.2.2.12
We will estimate the total color difference scores using the CIE76 formula. We will compare the mean change in total color difference from week 0 to each of weeks 8, 13, 26, and 65 at each anatomical location of interest. We will compare differences between arms with a t-test. We will carry out each of these analyses in the safety set.

<u>Analysis of Outcome Measure 10.2.2.13</u>
We will compare the subjective "mean change in coloration score" and "mean distress related to skin coloration score" for Arm 1 versus Arm 2 with t-tests. We will compare the proportion of participants with each degree of skin discoloration in Arm 1 and Arm 2 using subjective assessments of clinical imaging. We will provide point estimates together with Pearson Klopper exact 95% confidence intervals. We will carry out each of these analyses in the safety set.

<u>Analysis of Exploratory Outcome Measures</u>

A separate statistical analysis plan will be developed for the exploratory objectives and corresponding outcome measures.

11.0 PHARMACOLOGY PLAN

A combined intensive and sparse sampling strategy will be used to characterize the CFZ PK in Arms 1 and C. Sparse sampling will enrich these data and provide exposure over the study duration to assess the relationship between CFZ PK parameters and study related outcomes. Finally, RPT exposure will be assessed in Arms 1 and C.

11.1 Pharmacology Objectives

See section 1.4. Additionally, exposure-response will be evaluated using (1) a time-toevent analysis of time to stable culture conversion in the framework of non-linear modeling where more complex parametric hazard functions and continuous covariates (for example PK) can be evaluated **[111]**; (2) a model-based PK-PD analysis of the quantitative culture data (time to positivity in MGIT).

11.2 Pharmacology Study Design

11.2.1 Intensive PK Sampling: Arms 1 and C only

Forty participants (20 participants in Arm 1 and 20 participants in Arm C) will have an intensive PK sampling at week 2.

<u>Week 2</u>: The exact date and time, plus information about concurrent food intake **(±1 hour)** (yes/no, if yes also **date and** time of the meal) of the two doses of PHZE and CFZ prior to the intensive PK visit should be recorded on the eCRF. During the PK visit, plasma will be collected pre-dose (0h), and then PHZEC will be administered with food. Following PHZEC administration, plasma will be collected 1, 2, 4, 6, 8, 10, and 24 hours post-CFZ dose. The exact time of study medication doses, meals**, potential vomiting in connection to the drug administration,** and samples **collected** during the PK day should be recorded.

Twenty participants in Arm 1 will have an intensive PK sampling at week 13.

Week 13: The exact date and time, plus information about concurrent food intake (yes/no, if yes also time of the meal) of the two doses of PHZ-CFZ prior to the intensive PK visit should be recorded on the eCRF. Ideally, this PK visit should occur surrounding the last dose of CFZ in each respective experimental arm, but this is not required. PHZ -CFZ should be administered with food; plasma will be collected pre- PHZ -CFZ dose (0h), and then 1, 2, 4, 6, 8, 10, and 24 hours postCFZ dose. If the observed dose is the last CFZ dose, an additional sample should be drawn 48 hours post-CFZ dose. This final sample may be drawn up to 96 hours post-dose to accommodate weekend clinic closures. The exact time of doses, meals**, potential vomiting in connection to the drug administration,** and samples **collected** during the PK day should be recorded.

11.2.2 Sparse PK Sampling: Arms 1 and C only

For participants in Arm 1: Sparse PK sampling at weeks 2 and 13 will occur only for those not completing intensive PK visits. For all other occasions sampling will occur in all Arm 1 participants as described in section 6.1.

For participants in Arm C: Only one sparse PK sampling will occur at week 4.

Sparse plasma samples should ideally be collected 22-26 hours after the last dose of CFZ and RPT; however, the sample should be collected irrespective of time since last CFZ dose. The participants should be instructed to bring their dose of TB medication to their appointment for administration after the sparse PK is collected; the time of the dose administered after PK sampling is not required on the eCRF. The exact time of the PK sample collection should be recorded at all visits. The exact date and time, plus information about concurrent food intake (yes/no, if yes also time of the meal) **and potential vomiting in connection to the drug administration,** of the two doses of CFZ and RPT prior to the PK sample collection should be recorded on the eCRF.

Additional sparse PK samples collected after CFZ therapy is discontinued: Because of the long half-life of CFZ, samples for quantitation of CFZ will be collected after therapy is complete, according to section 6.1. Samples at these visits can be collected at any time during the study visit, irrespective of any medication dosing. The exact time of the PK sample should be recorded at all visits. The exact date and time of the final dose of CFZ should be collected.

11.2.3 Protein Binding: Arms 1 and C only

A single, serum sample will be collected from all participants at each study visit where intensive or sparse PK is measured to assess protein binding of CFZ. Both alpha- and beta-lipoprotein (apoA1 and apoB) can be assessed in one serum sample.

11.2.4 Pharmacogenetic Sampling

A single, whole blood sample will be collected at entry.

11.3 Primary and Secondary Data, Modeling and Data Analysis

11.3.1 CFZ PK Assessment

Standard non-compartmental techniques will be used to determine PK parameters (C_{min}, C_{max}, T_{max}, $AUC_{0\text{-}24h}$) from the intensive PK studies. The AUC will be determined using the trapezoidal rule. C_{min} will be taken as the minimum observed concentration after the observed dose. C_{max} will be taken as the maximum observed concentration. T_{max} is the time at which C_{max} occurs. The software package Phoenix (Certara Corporation) will be used for this analysis.

This non-compartmental analysis will be in conjunction with non-linear mixedeffects modeling to estimate the primary PK parameters (e.g., apparent oral clearance (CL/F), volume of distribution, rate of absorption). The model-based analysis will use the intensive PK samples as well as the sparse samples collected throughout the study period and all protein concentration data. The PK observations will be modeled continuously over time, taking in account each participant's actual dosing history. A covariate analysis to evaluate the potential impact of demographics and concomitant medications will be conducted. In addition to the primary population PK parameters, summaries of derived PK metrics, such as $AUC_{0\text{-}24h}$ and C_{max} will be reported for CFZ at each intensive PK visit. The derived secondary metrics will be compared with the noncompartmental analysis results in a posterior predictive check [**112**]. Furthermore, the accumulation of CFZ at weeks 2 and 4 in the arms with and without CFZ loading dose will be compared.

11.3.2 Rifapentine PK Assessment

Standard non-compartmental techniques will be used to determine PK parameters (C_{min}, C_{max}, T_{max}, $AUC_{0\text{-}24h}$, CL/F) from the intensive PK studies. The AUC will be determined using the trapezoidal rule. C_{min} will be taken as the minimum observed concentration after the observed dose. C_{max} will be taken as the maximum observed concentration. T_{max} is the time at which C_{max} occurs. Apparent oral clearance will be calculated as CL/F = dose/$AUC_{0\text{-}24h}$. The elimination half-life will be determined using regression analysis when possible. The software package Phoenix (Certara Corporation) will be used for this analysis.

11.3.3 Pharmacogenetic Analyses

Evaluation of pharmacogenetic associations between human pharmacogenetic polymorphisms that may affect metabolism, disposition and toxicity of study drugs and concomitant medications, and study drug efficacy, safety, and PK.

In all study participants who provide DNA for genetic testing, assays will be performed for polymorphisms in genes relevant to study drugs (e.g., *NAT2* which affects PK and hepatotoxicity of INH) and concomitant medications (e.g., *CYP2B6* which affects plasma PK and central nervous system side effects of EFV). Associations will be explored between selected genetic polymorphisms and outcome measures. Among the outcome measures to be studied will be study culture conversion, treatment failure, relapse, TB-related death, premature discontinuation of regimen other than due to violent death, natural disaster, or administrative censoring, premature discontinuation of regimen due to hyperpigmentation, gastrointestinal intolerance, ichthyosis, or QTcF interval prolongation, new Grade 3 or Grade 4 laboratory value or sign or symptom that is at least one grade increase from baseline, occurrence of SAEs, WHOQOL-BREF scores, CES-D scores, change in objective skin color parameters measured, plasma PK parameters for CFZ, plasma PK parameters for rifampicin, QTcF interval prolongation, participant subjective assessment of hyperpigmentation, physician subjective assessment of hyperpigmentation, and acquired CFZ resistance among participants failing treatment or relapsing.

11.3.4 Pharmacodynamic Analyses

Following finding the model that best describes the plasma PK characteristics of CFZ, we will next develop a linked PK and PD model to investigate relationships among the PK parameters of CFZ, and measures of safety and tolerance and TB outcomes. These PD models may be in the form of a linear or a sigmoid E_{max} relationship where, for example, higher CFZ concentrations are related to CFZ associated AEs. In addition to the pharmacologic assessment of PK-PD, PK measures will be used in the statistical analysis (see section 10.0) to evaluate the study outcomes associated with the CFZ-containing regimens.

11.4 Anticipated Outcomes

The pharmacologic evaluations will provide information on the steady-state PK of CFZ under two proposed dose strategies (Arms 1 and C). The measures of drug exposure determined from the PK data will be used to evaluate the relationship between the CFZ PK parameters achieved in the CFZ-containing regimens and study outcomes.

12.0 DATA COLLECTION AND MONITORING

12.1 Records to Be Kept

Electronic case report forms (eCRFs) will be provided for each participant. Participants must not be identified by name on any eCRFs. Participants will be identified by the patient identification number (PID) and study identification number (SID) provided by the ACTG DMC upon registration.

12.2 Role of Data Management

12.2.1 Instructions concerning entering study data on eCRFs will be provided by the ACTG DMC. Each CRS is responsible for keying the data in a timely fashion.

12.2.2 It is the responsibility of the ACTG DMC to assure the quality of computerized data for each ACTG study. This role extends from protocol development to generation of the final study databases.

12.3 Clinical Site Monitoring and Record Availability

12.3.1 Site monitors under contract to the NIAID will visit participating clinical research sites to review the individual participant records, including consent forms, eCRFs, supporting data, laboratory specimen records, and medical records (physicians' progress notes, nurses' notes, participants' hospital charts), to ensure protection of study participants, compliance with the protocol, and accuracy and completeness of records. The monitors also will inspect sites' regulatory files to ensure that regulatory requirements are being followed and sites' pharmacies to review product storage and management.

12.3.2 The site investigator will make study documents (e.g., consent forms, drug distribution forms, eCRFs) and pertinent hospital or clinic records readily available for inspection by the ACTG, local IRB/EC, the site monitors, the FDA, the NIAID, the OHRP, the industry supporter(s) or designee, other local, US, and international regulatory authorities for confirmation of the study data.

12.3.3 Monitoring visits may be conducted on-site or remotely. Remote visits may include remote source document verification using methods specified for this purpose by NIAID. Remote monitoring visits may be performed in place of, or in addition to, onsite visits to ensure the safety of study participants and data integrity [113]. The site will make available study documents for site monitors to review utilizing a secure platform that is HIPAA and 21 CFR Part 11 compliant. Potential platform options include: Veeva SiteVault, sitecontrolled SharePoint or cloud-based portal, direct access to Electronic Medical Record (EMR), and Medidata Rave Imaging Solutions. Other secure platforms that are 21 CFR Part 11 compliant may be utilized, as allowed by the DAIDS Office of Clinical Site Oversight (OCSO).

13.0 PARTICIPANTS

13.1 Institutional Review Board (IRB) Review and Informed Consent

This protocol, the informed consent documents (Appendices I, I-A, and I-B), and any subsequent modifications will be reviewed and approved by the IRB/EC responsible for oversight of the study. A signed consent form will be obtained from the participant (or legal guardian or person with power of attorney for participants who cannot consent for themselves). The consent form will describe the purpose of the study, the procedures to be followed, and the risks and benefits of participation. A copy of the consent form will be

given to the participant or legal guardian, and this fact will be documented in the participant's record.

13.2 Participant Confidentiality

All laboratory specimens, evaluation forms, reports, and other records that leave the site will be identified by coded number only to maintain participant confidentiality. All records will be kept locked. All computer entry and networking programs will be done with coded numbers only. Clinical information will not be released without written permission of the participant, except as necessary for monitoring by the ACTG, IRB/EC, FDA, NIAID, OHRP, other local, US, or international regulatory authorities as part of their duties, or the industry supporter(s) or designee.

13.3 Study Discontinuation

The study may be discontinued at any time by the ACTG, FDA, IRB/EC, NIAID, industry supporter, OHRP, or other government agencies as part of their duties to ensure that research participants are protected.

14.0 PUBLICATION OF RESEARCH FINDINGS

Publication of the results of this trial will be governed by ACTG policies. Any presentation, abstract, or manuscript will be made available for review by the industry supporter prior to submission.

15.0 BIOHAZARD CONTAINMENT

As the transmission of HIV and other blood-borne pathogens can occur through contact with contaminated needles, blood, and blood products, appropriate blood and secretion precautions will be employed by all personnel in the drawing of blood and shipping and handling of all specimens for this study, as currently recommended by the Centers for Disease Control and Prevention and the National Institutes of Health.

All dangerous goods and materials, including diagnostic specimens and infectious substances, must be transported using packaging mandated by CFR 42 Part 72. Please refer to instructions detailed in the International Air Transport Association (IATA) Dangerous Goods Regulations.

16.0 REFERENCES

1. Barry VC, Belton JG, Conalty ML, et al. A new series of phenazines (riminocompounds) with high antituberculosis activity. Nature 1957;179:1013-15.
2. Barry VC, Buggle K, Byrne J, Conalty ML, Winder F. Absorption, distribution and retention of the riminocompounds in the experimental animal. Ir J Med Sci 1960;416:345-52.

3. Barry VC, Conalty ML. Anti-tuberculosis activity in the phenazine series. II. N3substituted anilinoaposafranines (rimino-compounds) and some derivatives. Am Rev Tuberc 1958;78:62-73.
4. Chang YT. Chemotherapy of murine leprosy. IV. The effects of amithiozone (TB1/698), p-aminosalicylic acid (PAS), B 283 (aphenazine pigment), five antibiotics and three diphenylthiourea compounds on mouse leprosy. Int J Lepr 1955;23:167-80.
5. Van Deun A, Maug AK, Salim MA, et al. Short, highly effective, and inexpensive standardized treatment of multidrug-resistant tuberculosis. Am J Respir Crit Care Med 2010;182:684-92.
6. Aung KJ, Van Deun A, Declercq E, et al. Successful '9-month Bangladesh regimen' for multidrug-resistant tuberculosis among over 500 consecutive patients. Int J Tuberc Lung Dis 2014;8:1180-7.
7. Nunn AJ, Rusen ID, Van Deun A, et al. The evaluation of a standardized treatment regimen of anti-tuberculosis drugs for patients with multi-drug-resistant tuberculosis (STREAM): study protocol for a randomized controlled trial. Trials 2014;5:353.
8. Nunn AJ, Phillips P, Meredith S, et al. A trial of a shorter regimen for rifampin-resistant tuberculosis. N Engl J Med 2019;380:1201-13.
9. Tang S, Yao L, Hao X, et al. Clofazimine for the treatment of multidrug-resistant tuberculosis: prospective, multicenter, randomized controlled study in China. Clin Infect Dis 2015;60:1361-7
10. Wang Q, Pang Y, Jing W, Liu Y, et al. Clofazimine for the treatment of pulmonary extensively drug-resistant tuberculosis in China. Antimicrob Agents Chemother 2018;62:e02149-17.
11. Gopal M, Padayatchi N, Metcalfe JZ, et al. Systematic review of clofazimine for the treatment of drug-resistant tuberculosis. Int J Tuberc Lung Dis 2013;17:1001-7.
12. Padayatchi N, Gopal M, Naidoo R, et al. Clofazimine in the treatment of extensively drug-resistant tuberculosis with HIV coinfection in South Africa: a retrospective cohort study. J Antimicrob Chemother 2014;69:3103-7.
13. Mitnick CD, Shin SS, Seung KJ, et al. Comprehensive treatment of extensively drugresistant tuberculosis. N Engl J Med 2008;359:563-74.
14. Kuaban C, Noeske J, Rieder HL, Ait-Khaled N, et al. High effectiveness of a 12-month regimen for MDR-TB patients in Cameroon. Int J Tuberc Lung Dis 2015;19:517-24.
15. Abate G, Miorner H, Ahmed O, Hoffner SE. Drug resistance in Mycobacterium tuberculosis strains isolated from re-treatment cases of pulmonary tuberculosis in Ethiopia: susceptibility to first-line and alternative drugs. Int J Tuberc Lung Dis 1998;2:580-4.
16. Diacon AH, Dawson R, von Groote-Bidlingmaier F, et al. Bactericidal activity of pyrazinamide and clofazimine alone and in combinations with pretomanid and bedaquiline. Am J Respir Crit Care Med 2015;191:943-53.
17. Grosset JH, Tyagi S, Almeida DV, et al. Assessment of clofazimine activity in a secondline regimen for tuberculosis in mice. Am J Respir Crit Care Med 2013;188:608-12.

18. Tyagi S, Ammerman NC, Li SY, et al. Clofazimine shortens the duration of the first-line treatment regimen for experimental chemotherapy of tuberculosis. Proc Natl Acad Sci USA 2015;112:869-74.
19. **Ammerman NC, Swanson RV, Bautista EM, et al. Impact of clofazimine dosing on treatment shortening of the first-line regimen in a mouse model of tuberculosis. Antimicrob Agents Chemother 2018;62:e00636-18.**
20. Rosenthal IM, Tasneen R, Peloquin CA, et al. Dose-ranging comparison of rifampin and rifapentine in two pathologically distinct murine models of tuberculosis. Antimicrob Agents Chemother 2012;56:4331-40.
21. Li, SY, Tasneen R, Tyagi S, et al. Bactericidal and sterilizing activity of a novel regimen with bedaquiline, pretomanid, moxifloxacin and pyrazinamide in a murine model of tuberculosis. Antimicrob Agents Chemother 2017;61:e00913-17.
22. Tasneen R, Li, SY, Peloquin CA. Sterilizing activity of novel TMC207- and PA-824containing regimens in a murine model of tuberculosis. Antimicrob Agents Chemother 2011;55:5485-92.
23. Irwin SM, Gruppo V, Brooks E, et al. Limited activity of clofazimine as a single drug in a mouse model of tuberculosis exhibiting caseous necrotic granulomas. Antimicrob Agents Chemother 2014;58:4026-34.
24. Prideaux B, Via LE, Zimmerman MD, et al. The association between sterilizing activity and drug distribution into tuberculosis lesions. Nat Med 2015;21:1223-7.
25. Lanoix JP, Loerger T, Ormond A, et al. Selective inactivity of pyrazinamide against tuberculosis in C3HeB/FeJ mice is best explained by neutral pH of caseum. Antimicrob Agents Chemother 2015;60:735-43.
26. Mitchison DA. Assessment of new sterilizing drugs for treating pulmonary tuberculosis by culture at 2 months. Am Rev Respir Dis 1993;147:1062-3.
27. Te Brake LH, Russel FG, van den Heuvel JJ, et al. Inhibitory potential of tuberculosis drugs on ATP-binding cassette drug transporters. Tuberculosis (Edinb) 2016;96:150-7.
28. Browne SG, Hogenzeil LM. Apparent resistance of M. leprae to "B 663". Lepr Rev 1962; 33:185-9.
29. Warndorff-van Diepen T. Clofazimine-resistant leprosy, a case report. Int J Lepr and Other Mycobact Dis 1982;50):139-42.
30. Shetty VP, Uplekar MW, Antia NH. Primary resistance to single and multiple drugs in leprosy--a mouse footpad study. Lepr Rev 1996;67:280-6.
31. Ebenezer, GJ, Norman, G, Joseph, GA, et al. (2002): Drug resistant-Mycobacterium leprae-results of mouse footpad studies from a laboratory in south India. Indian J Lepr 2002;74:301-12.
32. Zhang S, Chen J, Cui P, et al. Identification of novel mutations associated with clofazimine resistance in Mycobacterium tuberculosis. J Antimicrob Chemother 2015;70:2507-10.
33. Andries K, Villellas C, Coeck N, et al. Acquired resistance of Mycobacterium tuberculosis to bedaquiline. PLoS One 2014;9:e102135.
34. Hartkoorn RC, Uplekar S, Cole ST. Cross-resistance between clofazimine and bedaquiline through upregulation of mmpL5 in Mycobacterium tuberculosis. Antimicrob Agents Chemother 2014;58:2979-81.

35. Almeida D, Loerger T, Tyagi S, et al. Mutations in pepQ confer low-level resistance to bedaquiline and clofazimine in Mycobacterium tuberculosis. Antimicrob Agents Chemother 2016;60:4590-9.
36. Somoskovi A, Bruderer V, Homke R, et al. A mutation associated with clofazimine and bedaquiline cross-resistance in MDR-TB following bedaquiline treatment. Eur Respir J 2015;45:554-7.
37. Cholo MC, Steel HC, Fourie PB, et al. Clofazimine: current status and future prospects. J Antimicrob Chemother 2012;67:290-8.
38. Bezerra EL, Vilar MJ, da Trindade Neto PB, Sato EI. Double-blind, randomized, controlled clinical trial of clofazimine compared with chloroquine in patients with systemic lupus erythematosus. Arthritis Rheum 2005;52:3073-8.
39. Lee SJ, Wegner SA, McGarigle CJ, et al. Treatment of chronic graft-versus-host disease with clofazimine. Blood 1997;89:2298-302.
40. Nix DE, Adam RD, Auclair B, et al. Pharmacokinetics and relative bioavailability of clofazimine in relation to food, orange juice and antacid. Tuberculosis (Edinb) 2004;84:365-73.
41. Schaad-Lanyi Z, Dieterle W, Dubois JP, et al. Pharmacokinetics of clofazimine in healthy volunteers. Int J Lepr Other Mycobact Dis 1987;55:9-15.
42. Yawalkar SJ, Vischer W. Lamprene (clofazimine) in leprosy. Basic information. Lepr Rev 1979;50:135-44.
43. Holdiness MR. Clinical pharmacokinetics of clofazimine. A review. Clin Pharmacokinet 11989;16:74-85.
44. Swanson RV, Adamson J, Moodley C, et al. Pharmacokinetics and pharmacodynamics of clofazimine in a mouse model of tuberculosis. Antimicrob Agents Chemother 2015;59:3042-51.
45. Ganesan S, Sunkara G, McNeeley D, Hughes D. Identification of optimal dose and dosing regimen of clofazimine for the treatment of multi-drug resistant tuberculosis based on pharmacokinetic modeling. Paper presented at: 46th World Conference on Lung Health of the International Union Against Tuberculosis and Lung Disease Conference; 2-6 December 2015; Cape Town, South Africa.
46. Levy L. Pharmacologic studies of clofazimine. Am J Trop Med Hyg 1974;23:1097-109.
47. Horita Y, Doi N. Comparative study of the effects of antituberculosis drugs and antiretroviral drugs on cytochrome P450 3A4 and P-glycoprotein. Antimicrob Agents Chemother 2014;58:3168-76.
48. Shimokawa Y, Yoda N, Kondo S, et al. Inhibitory potential of twenty five antituberculosis drugs on CYP activities in human liver microsomes. Biol Pharm Bull 2015;38:425-9.
49. Maartens G, Brill MJE, Pandie M, Svensson EM. Pharmacokinetic interaction between bedaquiline and clofazimine in patients with drug-resistant tuberculosis. Int J Tuberc Lung Dis 2018;22:26-9.
50. Mehta J, Gandhi IS, Sane SB, et al. Effect of clofazimine and dapsone on rifampicin (Lositril) pharmacokinetics in multibacillary and paucibacillary leprosy cases. Indian J Lepr 1985;57:297-310.

51. McNeeley, DF. Elucidating the role of clofazimine (lamprene®) in the treatment of MDRTB. MDR-TB Landscape Meeting, December 2014.
52. Jagannath C, Reddy MV, Kailasam S, et al. Chemotherapeutic activity of clofazimine and its analogues against Mycobacterium tuberculosis. In vitro, intracellular, and in vivo studies. Am J Respir Crit Care Med 1995;151:1083-6.
53. Reddy VM, Nadadhur G, Daneluzzi D, et al. Antituberculosis activities of clofazimine and its new analogs B4154 and B4157. Antimicrob Agents Chemother 1996;40:633-6.
54. Van Rensburg CE, Joone GK, Sirgel FA, et al. In vitro investigation of the antimicrobial activities of novel tetramethylpiperidine-substituted phenazines against Mycobacterium tuberculosis. Chemotherapy 2000;46:43-8.
55. Lu Y, Zheng M, Wang B, et al. Clofazimine analogs with efficacy against experimental tuberculosis and reduced potential for accumulation. Antimicrob Agents Chemother 2011;55:5185-93.
56. Xu J, Lu Y, Fu L, et al. In vitro and in vivo activity of clofazimine against Mycobacterium tuberculosis persisters. Int J Tuberc Lung Dis 2012;16:1119-25.
57. Ismail NA, Omar SV, Joseph L, et al. Defining bedaquiline susceptibility, resistance, cross-resistance and associated genetic determinants: a retrospective cohort study. EBioMedicine 2018;28:136-42.
58. **Abdelwahab MT, Wasserman S, Brust JCM, et al. Clofazimine pharmacokinetics in patients with TB: dosing implications. J Antimicrob Chemother 2020;75:3269-77.**
59. Diacon AH, Dawson R, von Groote-Bidlingmaier F. Bactericdal activity of pyrazinamide and clofazimine alone and in combinations with pretomanid and bedaquiline. Am J Respir Crit Care Med 2015;15:943-53.
60. Phillips P, Crook A, Li L, et al. Two-month Regimens Using Novel Combinations to Augment Treatment Effectiveness for Drug-sensitive Tuberculosis (TRUNCATE-TB). https://clinicaltrials.gov/ct2/show/NCT03474198
61. World Health Organization. WHO treatment guidelines for rifampicin- and multidrugresistant tuberculosis, 2018 update. Accessed at: https://www.who.int/tb/areas-ofwork/drug-resistant-tb/treatment/gdg-meeting-mdr-rr-tb-treatment-2018-update/en/
62. Nix DE, Adam RD, Auclair B, et al. Pharmacokinetics and relative bioavailability of clofazimine in relation to food, orange juice and antacid. Tuberculosis 2004;84:365-73.
63. Kroger A, Pannikar V, Htoon MT, et al. International open trial of uniform multi-drug therapy regimen for 6 months for all types of leprosy patients: rationale, design and preliminary results. Trop Med Int Health 2008;13:594-60.
64. Piubello A, Harouna SH, Souleymane MB, et al. High cure rate with standardised shortcourse multidrug-resistant tuberculosis treatment in Niger: no relapses. Int J Tuberc Lung Dis 2014;18:1188-94.
65. Furin JJ, Mitnick CD, Shin SS, et al. Occurrence of serious adverse effects in patients receiving community-based therapy for multidrug-resistant tuberculosis. Int J Tuberc Lung Dis 2001;5:648-55.

66. Xu HB, Jiang RH, Tang SJ, et al. Notice of retraction. Role of clofazimine in the treatment of multidrug-resistant tuberculosis: a retrospective observational cohort assessment. J Antimicrob Chemother 2014;69:e1.

67. Hwang TJ, Dotsenko S, Jafarov A, et al. Safety and availability of clofazimine in the treatment of multidrug and extensively drug-resistant tuberculosis: analysis of published guidance and meta-analysis of cohort studies. BMJ Open 2014;4:e004143.

68. Dannemann BR, Bakare N, De Marez T, et al. Corrected QT interval (QTcF) prolongation in a phase 2 open-label trial of TMC207 plus background regimen as treatment for MDR-TB: effect of coadministration with clofazimine [Abstract A-1259]. In Program and Abstracts of the Interscience Conference on Antimicrobial Agents and Chemotherapy (ICAAC); September 9-13, 2013; Denver, CO.

69. Chaisson RE, Keiser P, Pierce M, et al. Clarithromycin and ethambutol with or without clofazimine for the treatment of bacteremic Mycobacterium avium complex disease in patients with HIV infection. AIDS 1997;11:311-7.

70. Dorman SE, Goldberg S, Stout JE, et al. Substitution of rifapentine for rifampin during intensive phase treatment of pulmonary tuberculosis: study 29 of the tuberculosis trials consortium. J Infect Dis 2012;206:1030-40.

71. Alfarisi O, Alghamdi WA, Al-Shaer MH, Dooley KE, Peloquin, CA. Rifampin vs. rifapentine: what is the preferred rifamycin for tuberculosis? Expert Review of Clinical Pharmacolog 2017;10:1027-36.

72. Dorman SE, Savic RM, Goldberg S, et al. Daily rifapentine for treatment of pulmonary tuberculosis. a randomized, dose-ranging trial. Am J Respir Crit Care Med 2015;191:333-43.

73. Dooley, KE, Savic, RM, Gupte A, et al. Safety and PK of weekly rifapentine/isoniazid (3HP) in adults with HIV on dolutegravir. Conference on Retroviruses and Opportunistic Infections (CROI); March 4-7, 2019; Seattle, WA.

74. Podany AT, Bao Y, Swindells S, et al. Efavirenz pharmacokinetics and pharmacodynamics in HIV-infected persons receiving rifapentine and isoniazid for tuberculosis prevention. Clin Infect Dis 2015;61:1322-7.

75. Huang YS, Chern HD, Su WJ, Wu JC, Lai SL, Yang SY, et al. Polymorphism of the Nacetyltransferase 2 gene as a susceptibility risk factor for antituberculosis drug-induced hepatitis. Hepatology 2002;35:883-9.

76. Huang YS, Chern HD, Su WJ, Wu JC, Chang SC, Chiang CH, et al. Cytochrome P450 2E1 genotype and the susceptibility to antituberculosis drug-induced hepatitis. Hepatology 2003;37:924-30.

77. Roy PD, Majumder M, Roy B. Pharmacogenomics of anti-TB drugs-related hepatotoxicity. Pharmacogenomics 2008;9:311-21.

78. Ramachandran G, Swaminathan S. Role of pharmacogenomics in the treatment of tuberculosis: a review. Pharmacogenomics and Personalized Medicine 2012;5:89-98.

79. Sun F, Chen Y, Xiang Y, Zhan S. Drug-metabolising enzyme polymorphisms and predisposition to anti-tuberculosis drug-induced liver injury: a meta-analysis. Int J Tuberc Lung Dis 2008;12:994-1002.

80. Wang PY, Xie SY, Hao Q, Zhang C, Jiang BF. NAT2 polymorphisms and susceptibility to anti-tuberculosis drug-induced liver injury: a meta-analysis. Int J Tuberc Lung Dis 2012;16:589-95.

81. Goel U, Bajaj S, Gupta O, Dwivedi N, Dubey A. Isoniazid induced neuropathy in slow versus rapid acetylators: an electrophysiological study. J Assoc Physicians India 1992;40:671-2.

82. Yamamoto M, Sobue G, Mukoyama M, Matsuoka Y, Mitsuma T. Demonstration of slow acetylator genotype of N-acetyltransferase in isoniazid neuropathy using an archival hematoxylin and eosin section of a sural nerve biopsy specimen. J Neurol Sci 1996;135:51-4.

83. Kim RB. Organic anion-transporting polypeptide (OATP) transporter family and drug disposition. Eur J Clin Invest 2003;33(Suppl 2):1-5.

84. Chigutsa E, Visser ME, Swart EC, Denti P, Pushpakom S, Egan D, et al. The SLCO1B1 rs4149032 polymorphism is highly prevalent in South Africans and is associated with reduced rifampin concentrations: Dosing implications. Antimicrob Agents Chemother 2011;55:4122-7.

85. Fischel-Ghodsian N. Genetic factors in aminoglycoside toxicity. Pharmacogenomics 2005;6:27-36.

86. Selimoglu E. Aminoglycoside-induced ototoxicity. Current Pharmaceutical Design 2007; 13:119-26.

87. Guthrie O. Aminoglycoside induced ototoxicity. Toxicology 2008;249:91-6.

88. Guan M-X. Mitochondrial 12S rRNA mutations associated with aminoglycoside ototoxicity. Mitochondrion 2011;11:237-45.

89. Xie J, Talaska AE, Schacht J. New developments in aminoglycoside therapy and ototoxicity. Hear Res 2011;281:28-37.

90. Prezant TR, Agapian JV, Bohlman MC, et al. Mitochondrial ribosomal RNA mutation associated with both antibiotic–induced and non–syndromic deafness. Nature Genetics 1993;4:289-94.

91. Pacheu-Grau D, Gómez-Durán A, Montoya J, Ruiz-Pesini E. Influence of mtDNA genetic variation on antibiotic therapy. Pharmacogenomics 2010;11:1185-7.

92. Lubomirov R, Colombo S, di Iulio J, et al. Association of pharmacogenetic markers with premature discontinuation of first-line anti-HIV therapy: an observational cohort study. J Infect Dis 2011;203:246-57.

93. Ribaudo HJ, Daar ES, Tierney C, et al. Impact of UGT1A1 Gilbert variant on discontinuation of ritonavir-boosted atazanavir in AIDS Clinical Trials Group Study A5202. J Infect Dis 2013;207:420-5.

94. Vardhanabhuti S, Ribaudo HJ, Landovitz RJ, et al. Screening for UGT1A1 genotype in study A5257 would have markedly reduced premature discontinuation of atazanavir for hyperbilirubinemia. Open Forum Infect Dis 2015;2:ofv085.

95. Chen S, St Jean P, Borland J, et al. Evaluation of the effect of UGT1A1 polymorphisms on dolutegravir pharmacokinetics. Pharmacogenomics 2014;15:9-16.

96. Ward BA, Gorski JC, Jones DR, et al. The cytochrome P450 2B6 (CYP2B6) is the main catalyst of efavirenz primary and secondary metabolism: implication for HIV/AIDS

therapy and utility of efavirenz as a substrate marker of CYP2B6 catalytic activity. J Pharmacol Exp Ther 2003;306:287-300.

97. Kwara A, Lartey M, Sagoe KW, et al. Paradoxically elevated efavirenz concentrations in HIV/tuberculosis-coinfected patients with CYP2B6 516TT genotype on rifampincontaining antituberculous therapy. AIDS 2011;25:388-90.

98. Dooley KE, Denti P, Martinson N, et al. Pharmacokinetics of efavirenz and treatment of HIV-1 among pregnant women with and without tuberculosis coinfection. J Infect Dis 2015;211:197-205.

99. Luetkemeyer AF, Rosenkranz SL, Lu D, et al. Combined effect of CYP2B6 and NAT2 genotype on plasma efavirenz exposure during rifampin-based antituberculosis therapy in the STRIDE study. Clin Infect Dis 2015;60:1860-3.

100. di Iulio J, Fayet A, Arab-Alameddine M, et al. In vivo analysis of efavirenz metabolism in individuals with impaired CYP2A6 function. Pharmacogenet Genom 2009;19:300-9.

101. **Nahid P, Saukkonen J, Kenzie WRM, et al. Tuberculosis biomarker and surrogate endpoint research roadmap. Am J Respir Crit Care Med 2011;184:972-9.**

102. **Dooley KE, Phillips PPJ, Nahid P, Hoelscher M. Challenges in the clinical assessment of novel tuberculosis drugs. Adv Drug Deliv Rev 2016;102:116-22.**

103. **Walter ND. A novel PD metric: pre-rRNA measures drug impact on Mtb physiologic state. [Presentation]. TB Science Conference, Hyderabad, India, October 2019.**

104. Chiang CY, Lee JJ, Chien ST, et al. Glycemic control and radiographic manifestations of tuberculosis in diabetic patients. PLoS One 2014;3;9:e93397.

105. Colman R, Schupp JM, Hicks ND, et al. Detection of low-level mixed-population drug resistance in Mycobacterium tuberculosis using high fidelity amplicon sequencing. PLoS One 2015;10:e0126626.

106. Ralph A, Ardian M, Wiguna A, et al. A simple, valid, numerical score for grading chest xray severity in adult smear-positive pulmonary tuberculosis. Thorax 2010;65:863-9.

107. Boeree, MJ, Heinrich N, Aarnoutse R, et al.; PanACEA consortium. High-dose rifampicin, moxifloxacin, and SQ109 for treating tuberculosis: a multi-arm, multi-stage randomised controlled trial. Lancet Infect Dis 2017;17:29-39.

108. Gillespie SH, Crook AM, McHugh TD, et al.; REMoxTB Consortium. Four-month moxifloxacin-based regimens for drug-sensitive tuberculosis. N Engl J Med 2014;371:1577-87.

109. Merle CS, Fielding, K, Bah Sow O, et al.; OFLOTUB/Gatifloxacin for Tuberculosis Project. A four-month gatifloxacin-containing regimen for treating tuberculosis. N Engl J Med 2014;371:1588-98.

110. Jindani A, Harrison TS, Nunn A, et al. ; RIFAQUIN Trial Team. High-dose rifapentine with moxifloxacin for pulmonary tuberculosis. N Engl J Med 2014;371:1599-608.

111. Chigutsa E, Kashyap P, Denti P, et al. A time-to-event pharmacodynamic model describing treatment response in patients with pulmonary tuberculosis using days to positivity in automated liquid mycobacterial culture. Antimicrob Agents Chemother 2013;57:789-95.

112. Acharya C, Hooker AC, Türkyılmaz GY, et al. A diagnostic tool for population models using non-compartmental analysis: The ncappc package for R. Comput Methods Programs Biomed 2016;127:83-93.

113. **FDA Guidance on Conduct of Clinical Trials of Medical Products During the COVID-19 Public Health Emergency: Guidance for Industry, Investigators, and Institutional Review Boards, March 2020, Updated on January 27, 2021. Accessed at: https://www.fda.gov/media/136238/download.**

APPENDIX A: A5362 PROHIBITED AND PRECAUTIONARY MEDICATIONS

Following is a list of A5362 precautionary and prohibited medications. To avoid adverse drug interactions, package inserts of antiretroviral agents and other concomitant medications should be referenced whenever a concomitant medication is initiated or dose changed to avoid drug interaction adverse events.

PROHIBITED MEDICATIONS

Arm 1 and C Prohibited Medications

- Protease inhibitors (HIV and HCV)
- HIV entry and fusion inhibitors
- Elvitegravir/cobicistat
- Bictegravir
- Non-nucleoside reverse transcriptase inhibitors (except efavirenz)
- Prohibited medications during administration of DLM because of potential for QTc prolongation:
- Anti-arrhythmic medications (e.g., quinidine, procainamide, disopyramide, encainide, flecainide, sotalol, amiodarone, dofetilide, ibutilide)
- Certain antimalarials with QT-prolonging potential (e.g., halofantrine, quinine, chloroquine, artesunate/amodiaquine, dihydroartemisinin/piperaquine)
- Neuroleptics (e.g., phenothiazines, thioridazine, haloperidol, chlorpromazine, trifluoperazine, pericycline, prochlorperazine, fluphenazine, sertindole, sultopride, and pimozide)
- Tricyclic antidepressants: (e.g., amitriptyline, doxepin, desipramine, imipramine, and clomipramine)
- Some antimicrobials, including:
    - Fluoroquinolones (e.g., moxifloxacin and sparfloxacin)
    - Macrolides (e.g., azithromycin, clarithromycin, erythromycin) ○ Pentamidine
    - Triazole antifungal agents (e.g., fluconazole, itraconazole, posaconazole, voriconazole)
- Miscellaneous: Droperidol, domperidone, bepridil, diphemanil, probucol, levomethadyl, methadone, vinca alkaloids, arsenic trioxide, ziprasidone, terfenadine

Contact the protocol team with questions about other QT prolonging therapies

Arm 2 Prohibited Medications

- No specific prohibited medications. Follow local treatment guidelines for co-therapy with RHZE.

PRECAUTIONARY MEDICATIONS

Arm 1 and C Precautionary Medications

- Rifapentine is an inducer of cytochrome P4503A4 and 2C8/9. Therefore, it may increase the metabolism and decrease the activity of other co-administered drugs that are metabolized by these enzymes.

Arms 1, 2 and C Precautionary Medications

- Rifapentine and rifampicin are potent inducers of the cytochrome P450 enzyme system (CYP) and concomitant use with drugs metabolized by this enzyme system may result in suboptimal exposure of the co-administered medication. Carefully review all concomitant therapies during TB treatment.
- Acetaminophen
- Carbamazepine
- Chlorzoxazone
- Disulfiram
- Ketoconazole
- Phenytoin
- Propacetamol
- Theophylline
- Valproate
- Aluminum hydroxide containing antacids within 4 hours of ethambutol administration

Please contact the A5362 protocol team if you have any questions.

APPENDIX B: A5362 PERMITTED ANTIRETROVIRALS MEDICATIONS

Permitted antiretroviral metications are:

- Efavirenz 600 mg with dual NRTIs
- Dolutegravir 50 mg 12 hourly (reduced to 50 mg daily 2 weeks after stopping rifampicin) with dual NRTIs

APPENDIX I: SAMPLE INFORMED CONSENT
DIVISION OF AIDS
AIDS CLINICAL TRIALS GROUP (ACTG)

For protocol A5362

A5362, **FINAL Version 2.0, 08Jul2022**, A Phase IIc Trial of Clofazimine- and RifapentineContaining Treatment Shortening Regimens in Drug-Susceptible Tuberculosis: The CLO-FAST Study

SHORT TITLE FOR THE STUDY: A5362, **FINAL Version 2.0, 08Jul2022**, Clofazimine and Rifapentine for Drug-Susceptible Tuberculosis

SUMMARY

PURPOSE	This is a research study and your participation in this study is voluntary. The purpose of this study is to determine if taking a 3-month TB treatment including rifapentine/isoniazid/pyrazinamide/ethambutol/clofazimine (PHZEC) is better than taking a 6-month standard of care TB treatment. Studies have suggested a powerful effect for the combination of drugs in the 3-month treatment, such that standard TB treatment can be shortened by half. Standard of care TB treatment includes rifampicin/isoniazid/ pyrazinamide/ethambutol (RHZE) given for 2 months, then rifampicin/isoniazid (RH) given for 4 months. This study will also look at the tolerability of the study drugs, the effect the drugs have on the electrical activity of the heart, and will measure the level of these drugs in blood.
NUMBER OF PARTICIPANTS	There will be a total of 185 participants.
LENGTH OF STUDY	The study will last about 65 weeks.
REQUIRED ACTIVITIES	Blood and urine collections<br>• At most visits, blood will be collected from a vein in your arm.<br>• You will be asked to provide a urine sample.<br><br>Special procedures<br>• At several visits, you will have a chest X-ray and ECG (looks at the electrical activity of your heart.).<br>• At several visits, you will be asked to provide sputum samples by coughing deeply and then spitting into a cup. You may also be asked to provide a sputum sample by briefly breathing a mist of saltwater through a tube or a mask followed by coughing and spitting into a cup.

- Because the medication may cause skin discoloration, you will have pictures taken of **sites on** your **body (forehead, left cheek, right cheek, chin, and arm)** to look for any changes in the color of your skin.

RISKS

The following are possible:

Clofazimine Rare:

- Colicky (cramping) or burning abdominal or stomach pain
- Mental depression
- Loss of appetite
- Changes in taste
- Dryness, burning, itching, or irritation of the eyes
- Increased sensitivity of skin to sunlight
- Bloody or black, dark red stools
- Disturbance in heart rhythm

More common:

- Diarrhea, nausea or vomiting, or abdominal pain
- Dry, rough, or scaly skin, which may or may not be itchy
- Red-brown skin, urine, sweat, tears, or stool discoloration. This side effect goes away after stopping clofazimine but it may take months to (rarely) years.

Rifapentine/rifampicin/isoniazid/pyrazinamide/ethambutol

- Tears, sweat, saliva, feces, urine may turn orange-colored while taking medicines; contact lenses may be stained permanently

BENEFITS

You may receive a direct benefit from participating in this study, but no guarantee can be made.

OTHER CHOICES

Instead of being in this study, you have the option of continuing with your current treatment or starting a new treatment under the care of your regular doctor or other health care provider. You also have the choice to be referred to the National Tuberculosis Program for TB treatment according to local standard of care, or the option of no treatment.

INTRODUCTION:

You are being asked to take part in this research study because you have pulmonary tuberculosis (TB) of your lungs that can treated by drugs generally used to treat it. This is known as drug-susceptible TB. If someone is infected with TB bacteria that are fully susceptible, it means that all of the TB drugs will be effective (work against TB) as long as they are taken

properly. It still means that several drugs need to be taken together to provide effective TB treatment.

This study is sponsored by the National Institutes of Health (NIH). The doctor in charge of this study at this site is: (*insert name of Principal Investigator*). Before you decide if you want to be a part of this study, we want you to know about the study.

This is a consent form. It gives you information about this study. The study staff will talk with you about this information. You are free to ask questions about this study at any time. If you agree to take part in this study, you will be asked to sign this consent form. You will get a copy to keep.

WHY IS THIS STUDY BEING DONE?

The purpose of this study is to determine if taking a 3-month TB treatment including rifapentine/isoniazid/pyrazinamide/ethambutol/clofazimine (PHZEC) is better than taking a 6month standard of care TB treatment. Studies have suggested a powerful effect for the combination of drugs in the 3-month treatment, such that standard TB treatment can be shortened by half. Standard of care TB treatment includes the combination of four drugs, rifampicin/isoniazid/ pyrazinamide/ethambutol (RHZE) given for 2 months, then rifampicin/isoniazid (RH) given for 4 months. The safety and effectiveness of the PHZEC arms will be compared to standard combination TB therapy. This study will also look at the tolerability of the study drugs, the effect the drugs have on the electrical activity of the heart, and will measure the level of these drugs in blood.

WHAT DO I HAVE TO DO IF I AM IN THIS STUDY?

If you decide to take part in this research study, you will be asked to sign this consent form. A screening visit will be done to make sure you are eligible to join the study. If you are found eligible, you will be in the study for 65 weeks. The schedule of visits and study procedures are explained in Appendix I-A.

Information collected at screening

There is some information that we collect on everyone who is screened for an ACTG study. The ACTG is a group of researchers that conducts research related to HIV and TB infection. As part of your screening visit, some demographic (for example, age, gender, race), clinical (for example, disease condition, diagnosis), and laboratory (for example, CD4 cell count, viral load) information will be collected from you. We also collect information on whether you use (or have used) IV drugs.

We will collect this information even if you do not enroll in this study. This information is collected so that ACTG researchers may determine whether there are patterns and/or common reasons why people do not join a study.

CAN I CHOOSE THE TYPES OF RESEARCH THAT MY SAMPLES AND INFORMATION ARE USED FOR?

Some of your blood and/or sputum will be stored and used for study-required immunologic (structure and function of the immune system) testing and pharmacogenetic testing (testing of material passed from parent to child that determines the makeup of the body and mind that can affect your response to the study drugs). **Some of your blood may be used for whole genome sequencing (researchers look at all of your genes and at almost all of your DNA).** Some of these samples will be shipped and stored outside of the country from which they are collected.

Identifiers will be removed from your samples and from any private information that has been collected about you. This means that no one looking at the labels or at other information will be able to know that the samples or information came from you.

The tests described above are required by this study. If you do not agree to the storage or testing that has been described above, you should not join this study.

If you enter the study

There are three treatment groups in this study; Arm 1, Arm 2, and Arm C. At the study entry visit, you will be asked if you agree to be in Arm C. In Arm C, participants will have fewer study visits than participants in Arm 1 or Arm 2. If you agree to be in Arm C, you will be randomized (like the flip of a coin) to be in Arm 1, Arm 2, or Arm C. Once 20 participants agree to be in Arm C, enrollment in Arm C will stop. If you are not willing to consent to be in Arm C, you will be randomized (like the flip of a coin) to be in Arm 1 or Arm 2. More participants will be assigned to Arm 1 than to Arm 2 or Arm C, so it is more likely that you will be in this arm. You will not be able to choose your group, though you and the study staff will know which group you are in.

- If you are in Arm 1, you will take rifapentine/isoniazid/pyrazinamide/ethambutol (PHZE) + clofazimine (CFZ) 300 mg for 2 weeks; then PHZE + CFZ 100 mg for 6 weeks; then rifapentine/isoniazid/pyrazinamide (PHZ) + CFZ 100 mg for 5 weeks, for a total of 13 weeks of study treatment. Study treatment may be extended to 17 weeks on study treatment if you continue to have symptoms consistent with active TB, and tests show that your TB infection has worsened or has not improved. If this continues after 17 weeks of study treatment, you will remain on the study and receive treatment consistent with standard of care for TB.
- If you are in Arm 2, you will take rifampicin/isoniazid/pyrazinamide/ethambutol (RHZE) for 8 weeks; then rifampicin/isoniazid (RH) for 18 weeks, for a total of 26 weeks of study treatment. Thus the standard of care for TB treatment.
- If you are in Arm C, you will take rifapentine/isoniazid/pyrazinaminde/ethambutol (PHZE) + clofazimine (CFZ) 100 mg daily for 4 weeks. This will be followed by standard of care TB treatment (received from your health care provider) that will include 4 weeks of rifampicin/isoniazid/pyrazinamide/ethambutol (RHZE) and 18 weeks of rifampicin/isoniazid (RH).
- You will also take pyridoxine (vitamin B6) with each dose of isoniazid prescribed by your health care provider. Vitamin B6 plays an important role in the body. It is needed to maintain the health of nerves, skin, and red blood cells. Pyridoxine has been used to prevent or treat a certain nerve disorder (peripheral neuropathy) caused by certain medications (such as isoniazid).

If you join the study and are living with HIV (the virus that causes AIDS), your doctor will talk with you about the best options for treating your HIV. If you are taking anti-HIV drugs that are

not recommended to be used with the TB drugs used in this study, the doctor will advise you to change your HIV drug treatment.

HOW MANY PEOPLE WILL TAKE PART IN THIS STUDY?

About 185 participants will take part in the study; 110 in Arm 1, 55 in Arm 2, and 20 in Arm C.

HOW LONG WILL I BE IN THIS STUDY?

You will be in the study for 65 weeks.

WHY WOULD THE DOCTOR TAKE ME OFF THIS STUDY EARLY?

The study doctor may need to take you off the study early if:

- You request to be taken off the study.
- Your doctor decides the study is no longer in your best interest or that it is unlikely that you will be able to comply with the study requirements.
- The study is stopped or cancelled.

The study doctor may also need to take you off the study drug(s) without your permission if:

- Continuing the study drug(s) may be harmful to you.
- You need a treatment that you may not take while on the study.
- You are found to have TB with INH or **rifampicin** resistance.
- Your screening, entry, and first post-entry visit sputum cultures all fail to grow *Mtb* complex.
- You are a woman who becomes pregnant or is breast-feeding.
- You are not able to take the study drug(s) as required by the study.

If you must stop taking the study drug(s) before the study is over, you will be asked to complete an Early Treatment Discontinuation Visit, and the study doctor will ask you to continue to be part of the study and return for all study visits and procedures through week 65.

IF I HAVE TO PERMANENTLY STOP TAKING STUDY-PROVIDED DRUGS, OR STANDARD COMBINATION TB THERAPY, HOW WOULD THESE BE PROVIDED?

During the study:
If you must permanently stop taking study-provided drugs or standard combination TB therapy before your study participation is over, the study staff will discuss other options that may be of benefit to you.

After the study:

After you have completed your study participation, the study will not be able to continue to provide you with CFZ or standard combination TB therapy that you received on the study. If continuing to take these or similar drugs would be of benefit to you, the study staff will discuss how you may be able to obtain them.

WHAT ARE THE RISKS OF THE STUDY?

The drugs used in this study may have side effects, some of which are listed below. Please note that these lists do not include all the side effects seen with these drugs. These lists include the more serious or common side effects with a known or possible relationship. Many of the side effects of the study TB drugs have similar side effects as other treatments you would receive for your TB infection. If you have questions concerning the additional study drug side effects, please ask the medical staff at your site.

There is a risk of serious and/or life-threatening side effects when non-study medications are taken with the study medications, and use of a non-standard regimen may have unexpected consequences. For your safety, you must tell the study doctor or nurse about all medications you are taking before you start the study and also before starting any new medications while on study. Also, you must tell the study doctor or nurse before enrolling in any other clinical trials while on this study.

<u>Clofazimine</u> Rare:

- Colicky or burning abdominal or stomach pain
- Mental depression, followed with use of a validated questionnaire
- Loss of appetite
- Changes in taste
- Dryness, burning, itching, or irritation of the eyes
- Increased sensitivity of skin to sunlight
- Bloody or black, dark red stools
- Disturbance in heart electrical activity (prolonging the QTcF interval). While on the study, your heart rhythm will be monitored with an ECG test at multiple visits. If a disturbance in electrical activity is found, you will be monitored more closely with once weekly ECG testing. The severity of the disturbance in electrical activity will be evaluated with specific criteria. Depending on the severity, clofazimine and other study medications may be discontinued, and in extreme cases you may even be hospitalized for monitoring until your QTcF interval improves. In this case, you would be referred to the National Tuberculosis Program for TB treatment according to local standard of care.

More common:

- Diarrhea, nausea or vomiting, or abdominal pain
- Dry, rough, or scaly skin, which may or may not be itchy
- Red-brown skin, urine, sweat, tears, or stool discoloration. This side effect goes away after stopping clofazimine but it may take months to (rarely) years.

Rifapentine

- Some medications used to treat TB may be associated with diarrhea (loose or watery bowels), including bloody diarrhea, which may be serious.
- Reddish coloring of sputum and breast milk. Tears, sweat, saliva, feces, urine may turn orange-colored. Stains to your dentures or contact lenses that may not go away.
- Liver damage; if you develop any of the following symptoms, you should call your doctor right away:
    - Unexplained loss of appetite ○ Nausea and or vomiting ○ Pale colored stools ○ Yellowing of the eyes or skin
    - Pain in the upper abdomen (where your stomach is) ○ Dark urine
- Additional side effects may include:
    - Loss of appetite ○ Low blood counts ○ Low blood sugar
    - Decreased effectiveness of hormonal birth control (contraceptives) and other medications, including some anti-HIV medications. Tell your doctor about all medications that you are taking.
- **Although rare, flu-like symptoms or a severe allergic reaction may occur. The reaction should go away when you stop the rifapentine. Symptoms may include:**
    - **Fever** ○ **Rash**
    - **Inflammation of the liver** ○ **Low blood pressure** ○ **Muscle aches**
    - **Temporary decrease in white blood cells called lymphocytes** ○ **Headache** ○ **Nausea** ○ **Vomiting**

Rifampicin/isoniazid/pyrazinamide/ethambutol Common, not serious:

- Tears, sweat, saliva, feces, urine may turn orange-colored while taking medicines; contact lenses may be stained permanently

Occasional, some may be serious:

- Upset stomach, nausea, vomiting, belly pain
- Diarrhea

- Poor appetite
- Dizziness
- Rash
- Headaches
- Low blood count
- Inflammation of the liver
- Joint pain
- Flu-like symptoms: fever, chills, headache, body ache, fatigue
- Numbness and/or tingling of arms, legs

Rare and serious:

- Allergic reaction that may cause rash, low blood pressure, shortness of breath, swelling of face or throat
- Low blood platelets
- Loss of vision (temporary or permanent)
- Seizures or trouble thinking or depression
- Kidney damage

Risks of Nitrosamine Impurities

The US Food and Drug Administration (FDA) has been investigating the presence of impurities called nitrosamines in some types of medications, including rifapentine (RPT). Low amounts of nitrosamines are common in water and foods, including cured and grilled meats, dairy products, and vegetables. Everyone is exposed to some level of nitrosamines through their diet. Some types of nitrosamines have been shown to cause cancer in laboratory animals. Nitrosamine impurities may increase the risk of cancer if people are exposed to them above acceptable levels.

The study drug RPT contains small amounts of a nitrosamine impurity. The FDA recently noted that, because tuberculosis is a potentially deadly disease that affects the lungs and sometimes other parts of the body, the risk of not taking the medicine outweighs any potential risk from nitrosamine.

Seventeen weeks of daily RPT has been shown to be safe for treatment of active TB. Use of the currently available RPT for treatment of active TB has clear benefits, while the cancer risk is very low. All medications have risks and benefits. You should talk with your study doctor about any concerns.

Risks of Drug Interactions (Arm 1)

Although felt to be unlikely, it is currently unknown if clofazimine (CFZ) may interact with antiHIV drugs. If you are living with HIV, your HIV viral load (how much HIV is in your blood) will be monitored during study treatment to find out if CFZ interacts with your anti-HIV drugs. This information can be used by your treatment site to make decisions about your HIV treatment.

Risks of Looking for Changes in Skin Color

There is no risk to you when pictures of your skin are taken.

Risks of Drawing Blood

Taking blood may cause some discomfort, bleeding, bruising, and/or swelling where the needle enters the body, lightheadedness, and in rare cases, fainting or infection.

Risks of Electrocardiogram (ECG)

The electrodes from the ECG machine are small, plastic patches that stick to the skin. Therefore, you may experience mild irritation, slight redness and itching on your skin where the electrodes are placed.

Risks of Chest X-Rays

The amount of radiation exposure from chest X-rays is very low. Your chest X-rays will be read at a central location by two Board Certified Chest Radiologists. Your screening chest X-ray will also be read at your local clinic. Chest X-ray reading is used to determine how advanced your TB disease is.

Risks of Social Harm

It is possible that participating in this study will make it difficult for you to keep your HIV or TB status secret from people close to you. This may lead to unwelcome discussions about or reactions to your HIV or TB status. Please talk with the study staff if you have any concerns about this.

ARE THERE RISKS RELATED TO PREGNANCY?

It is not known if the drug or drug combinations in this study harm unborn babies. If you are a woman and having sex that could lead to pregnancy, you must agree not to become pregnant. If you are participating in sexual activity that could lead to pregnancy, you must agree to use an adequate method of contraception (acceptable forms of contraception include condoms, intrauterine device or intrauterine system, cervical cap with spermicide, and diaphragm with spermicide; hormonal birth control alone is not acceptable) before you start taking the study drugs, while you are taking the study drugs, and for 30 days after you stop taking the study drug.

If you can become pregnant, you must have a pregnancy test before you enter this study. The test must show that you are not pregnant. If you think you may be pregnant at any time during the study, tell your study staff right away. The study staff will talk to you about your choices. You will be referred for TB treatment and prenatal care, and if applicable, to a local HIV clinic. You will be followed on the study through the end of the study period.

If you become pregnant while on study, the study staff would like to obtain information from you about the outcome of the pregnancy (even if it is after your participation in the study ends). If you are taking anti-HIV drugs when you become pregnant, your pregnancy will be reported to the Antiretroviral Pregnancy Registry, an international database that collects information about pregnancies in women taking anti-HIV drugs. This report will not use your name or other information that could be used to identify you.

ARE THERE BENEFITS TO TAKING PART IN THIS STUDY?

If you take part in this study, there may be a direct benefit to you, but no guarantee can be made. It is also possible that you may receive no benefit from being in this study. Information learned from this study may help others who have TB.

WHAT OTHER CHOICES DO I HAVE BESIDES THIS STUDY?

Instead of being in this study you have the choice of:

- Treatment with prescription drugs available to you**. You have the option of continuing with your current treatment or starting a new treatment under the care of your regular doctor or other health care provider.**
- Referral to the National Tuberculosis Program for TB treatment according to local standard of care**. You have the option to receive standard therapy, which contains rifampicin. The standard therapy rifampicin also contains nitrosamine levels similar to the studyprovided drugs that are above normally acceptable levels. Any possible nitrosamine risk is therefore likely to be similar.**
- No treatment**.**

Please talk to your doctor about these and other choices available to you. Your doctor will explain the risks and benefits of these choices.

WHAT ABOUT CONFIDENTIALITY?

Efforts will be made to keep your personal information confidential. We cannot guarantee absolute confidentiality. Your personal information may be disclosed if required by law. Any publication of this study will not use your name or identify you personally.

Your records may be reviewed by the U.S. Food and Drug Administration (FDA), the ACTG, the U.S. Office for Human Research Protections (OHRP), or other local, US, and international regulatory entities as part of their duties (*insert name of site*) institutional review board (IRB) or Ethics Committee (a committee that protects the rights and safety of participants in research), National Institutes of Health (NIH), study staff, study monitors, drug companies supporting this study, and their designees.

All information collected about you as part of the study will be sent securely to the ACTG Statistical and Data Management Center in the United States for combining with information from other study participants and statistical analysis of study results. Your name and other personal identifiers will not be sent. Your research site is responsible for sending your information in accordance with the laws, regulations, and policies of your country and research site.

A description of this clinical trial will be available on **https://www.**ClinicalTrials.gov, as required by US law. This **Web site** will not include information that can identify you. At most, the **Web site** will include a summary of the results. You can search this **Web site** at any time.

WHAT ARE THE COSTS TO ME?

Taking part in this study may lead to added costs to you and your insurance company. In some cases it is possible that your insurance company will not pay for these costs because you are taking part in a research study. There is no cost to you for study medications, standard of care TB medications, study-related visits, physical examinations, laboratory tests, or other study procedures. Anti-HIV medicines and vitamin B6 will not be provided by the study.

WILL I RECEIVE ANY PAYMENT?

You may be reimbursed for your time and travel expenses as part of your participation in this study. *(Site to insert site-specific information about payment.)*

WHAT HAPPENS IF I AM INJURED?

If you are injured as a result of being in this study, you will be given immediate treatment for your injuries. **The NIH does not have a mechanism to provide direct compensation for research related injury.** [*Sites: Please modify (if necessary) and insert one of these two statements, as appropriate to your site. If your site is required to carry CTI, this must be indicated in the informed consent.]*

- *This site has clinical trials insurance. This insurance will allow the site to provide you with monetary compensation if you suffer harm as a result of participating in this research study.*

The cost for this treatment will be charged to you or your insurance company. You will not be giving up any of your legal rights by signing this consent form.

WHAT ARE MY RIGHTS AS A RESEARCH PARTICIPANT?

Taking part in this study is completely voluntary. You may choose not to take part in this study or leave this study at any time. Your decision will not have any impact on your participation in other studies conducted by NIH and will not result in any penalty or loss of benefits to which you are otherwise entitled.

We will tell you about new information from this or other studies that may affect your health, welfare, or willingness to stay in this study. If you want the results of the study, let the study staff know.

WHAT DO I DO IF I HAVE QUESTIONS OR PROBLEMS?

For questions about this study or a research-related injury, contact:
- Name of the investigator or other study staff
- Telephone number of above

For questions about your rights as a research participant, contact:
- Name or title of person on the IRB or other organization appropriate for the site •
  Telephone number of above

SIGNATURE PAGE

If you have read this consent form (or had it explained to you), all your questions have been answered and you agree to take part in this study, please sign your name below.

______________________________	______________________________
Participant's Name (print)	Participant's Signature and Date
______________________________	______________________________
Participant's Legal Representative (print)<br>(As appropriate)	Legal Representative's Signature and Date
______________________________	______________________________
Study Staff Conducting<br>Consent Discussion (print)	Study Staff's Signature and Date
______________________________	______________________________
Witness's Name (print) (As<br>appropriate)	Witness's Signature and Date

APPENDIX I-A: A5362 STUDY VISITS

The study staff can answer any questions you have about individual study visits, and how long they will last, or about the tests that will occur. The table below can be used as a quick reference for you, along with the explanations that follow.

I. Study Schedule

Evaluation or Procedure	Screening[1]	Entry[2]	Day 3	Visits During Treatment [3]	Follow-Up Visits[4]	Suspected TB Treatment Failure or Recurrence or Poor Treatment Response[5]	Early Treatment or Study Discontinuation[6]
Consent/contact information	√						
HIV status	√						
Medical, medication history	√	√					
Physical exam/concomitant medications/skin changes	√	√	√	√	√	√	√
Blood collection (see blood tests below)	√	√	√	√	√	√	√
Pregnancy test	√	As clinically indicated					
Urinalysis		√					
Sputum collection	√	√		√	√	√	√
**Sputum RS Ratio (optional)**		√		√		√	√
Chest X-ray	√			√		√	
ECG	√		√	√			√
Directly observed therapy **(DOT)**				√			
Quality of life questions		√		√	√		√
Questions about depression		√		√	√		√
Questions about your experience with treatment				√			

1 Screening Visit: After you have read and signed the consent form, you will have several tests done to make sure that you meet the requirements for joining the study.

2 Entry Visit: If you are able to join the study, you will enter the study and receive your treatment assignment.

3 Visits During Treatment:
If you are in Arm 1, you will have a visit at day 3, **weekly through week 4, and then** biweekly visits to week 12, and then at week 13; **Arm 1 intensive PK participants will also have visits at weeks 1 and 3**.
If you are in Arm 2, you will have a visit at day 3, then biweekly visits to week 12, and then at weeks 13, 17, 21, and 26.
If you are in Arm C, you will have a visit at day 3, and then weekly visits to week 4.

4 Follow-up Visits:
If you are in Arm 1, your follow-up visits will be at weeks 17, 21, 26, 34, 42, 52, and 65.
If you are in Arm 2, your follow-up visits will be at weeks 34, 42, 52, and 65.
If you are in Arm C, your follow-up visits will be at weeks 8, 26, and 65.

[5] Suspected TB Treatment Failure, or TB Recurrence, or Poor Treatment Response: If the study drugs fail to treat your TB infection or if you have a recurrent TB infection, you will be asked to come in for a visit. For example, you may need to come in for a visit if:

- You have signs or symptoms that are consistent with TB, have a chest X-ray that indicates worsening TB, or have sputum test positive for TB at or after week 13 (Arm 1) or week 17 (Arm 2)
- The study doctor is considering extending or changing your TB treatment [6] Early Treatment or Study Discontinuation:

If you leave the study early, you will be asked to come in for an early study discontinuation. You will leave the study early if, after you enter the study, tests show that you do not have TB, or if your TB infection is resistant to anti-TB drugs (some drugs are not effective at treating the TB infection).

If you discontinue study drug early, you will come in for an Early Treatment Discontinuation Visit, and you will be encouraged to continue on study and receive all evaluations through week 65.

II. Explanation of Evaluations

Below are descriptions of the evaluations. You will be told the results of all tests performed with the exception of those tests to look at the levels of study drugs in your blood and for future ACTG-approved testing.

Consent and contact information collected
After you read the consent and have had a chance to ask questions about the study, you will sign the consent form if you want to join the study. You will also be asked how to be contacted in case you miss a visit or there are problems with your tests, and whether you give the study team permission to contact you.

HIV Status
If there is no record of your HIV status, a HIV test will be done. If a HIV test has to be done, you may have to sign a separate consent form before this is done. You will be told the result of the HIV test as soon as it is available.

Physical Examination
You will have a physical exam and will be asked questions about your health and about any medicines you are taking now. At the entry visit, your medical and medication history will be taken.

Skin Changes
If you are in Arm 1 or Arm 2, a small camera will be used to take pictures of your forehead, left cheek, right cheek, chin**, and arm** to look for and measure any changes in the color of your skin. Each area will be washed with alcohol skin cleanser. A small circular mark will be made on each of area of your skin where the photograph will be taken. You will be asked not to apply any creams or lotions for at least 1 hour prior to being measured. Identifiable parts of your face in the pictures will be blocked out so that others should not be able to identify you.

Blood Collection
Blood may be collected at any visit. If you have Intensive PK Sampling, as described below, up to about 18 tablespoons of blood may be collected from you (in total). If you

do not have Intense PK Sampling, up to about 13 tablespoons of blood may be collected from you (in total). Blood collected from you will be used for various tests during the study, including:

***Stored serum*:**
Blood will be stored and may be tested after the study is over to see if you had other infections that occurred during the study. Blood will be collected at Entry (all participants), at end of treatment (Weeks 13 and 26 for Arms 1 and 2, respectively), and at end of follow-up (Week 65, Arms 1 and 2 only).

Hematology, chemistry, and liver function tests:
These are routine blood tests for safety. You will be asked not to eat or drink for a period of time before you have blood collected for these tests.
If you are in Arm 1 you will have blood drawn at screening, day 3, and weeks 1, 2, 4, 8, 13, and 26.
If you are in Arm 2, you will have blood drawn at screening, day 3, and weeks 2, 4, 8, 13, and 26.
If you are in Arm C, you will have blood drawn at screening, day 3, and weeks 1, 2, and 4.
Your blood drawn at entry will be tested for diabetes.

HIV viral load:
This is a test that shows how much HIV is in your blood.
If you are living with HIV and are in Arm 1 or Arm 2, you will have blood drawn for this test at entry and week 12.
If you are living with HIV and are in Arm C, you will have blood drawn for this test at entry and week 8.

CD4+ count:
This is a test that shows how many infection-fighting cells you have in your blood. If you are living with HIV and are in Arm 1, Arm 2, or Arm C, you will have blood drawn for this test at screening.

Pharmacokinetic (PK) sampling:
Blood samples will be taken during the study to measure the levels of anti-TB drugs in the body.

- *Intensive PK sampling*:
    - Some participants in Arm 1 will have blood samples drawn at week 2 and week 13. If you are in Arm 1 and are chosen to have this testing, blood samples will be *drawn*:
- At the week 2 visit, eight blood samples will be taken over a 24-hour period, before you take your medicine and then at 1, 2, 4, 6, 8, 10, and 24 hours later. You may have to stay in the clinic for when some or all of these samples are taken.
- At the week 13 visit, nine blood samples will be taken over a 48-hour period, before you take your medicine, and then at 1, 2, 4, 6, 8, 10, 24, and 48 hours later. You may have to stay in the clinic for when some or all of these samples are taken.
- If you are in Arm C, at the week 2 visit, eight blood samples will be taken over a 24-hour period, before you take your medicine and then at 1, 2, 4, 6, 8, 10, and 24 hours later.

- *Sparse PK sampling*:
  - If you are in Arm 1 and not chosen to have intensive PK sampling at weeks 2 and 13, you will have one blood sample drawn for sparse PK testing at weeks 2, 4, 8, 12, 13, 17, 21, 26, 34, at the time of suspected TB treatment failure, TB recurrence, or poor treatment response, and at the early treatment or study discontinuation visit.
  - If you are in Arm 1 and have been chosen to have intensive PK sampling at weeks 2 and 13, you will have one blood sample drawn for sparse PK testing at weeks 4, 8, 12, 17, 21, 26, 34, at the time of suspected TB treatment failure, TB recurrence, or poor treatment response, and at the early treatment or study discontinuation visit.
  - If you are in Arm C, you will have one blood sample drawn for sparse PK testing at the week 4 visit.

Lipoprotein test
You will have blood samples collected to measure the levels of lipoprotein (substances made up of protein and fat) in your blood. Results of this test help to determine your risk of developing heart disease.

Pharmacogenetic analyses:
You will have one blood sample collected and stored at entry to look at how your genes (material passed from parent to child that determines the makeup of the body and mind) affect your response to the study drugs.

Pregnancy Test
If you are a female who is able to become pregnant, you will be asked to give a small urine or blood sample (about 5 mL or 1 teaspoon) for a pregnancy test.

If you are in Arm 1, this test will be done at screening, at week 13, and at other times during the study if you think you might be pregnant.
If you are in Arm 2, this test will be done at screening, and at other times during the study if you think you might be pregnant.
If you are in Arm C, this test will be done at screening, at week 4, and at other times during the study if you think you might be pregnant.

Urinalysis
You will be asked to provide a urine sample at entry.

Sputum Collection
You will be asked to provide sputum samples (a mixture of saliva and mucus coughed up from the respiratory tract) by coughing deeply and then spitting into a cup.

If you are in Arm 1, this procedure will be done at screening, entry, weekly through week 4, biweekly through week 12, and then at weeks 13, 17, 21, 26, 34, 42, 52, 65, at the time of suspected TB treatment failure, TB recurrence, or poor treatment response, and at the early treatment or study discontinuation visit.

If you are in Arm 2, this procedure will be done at screening, entry, bi-weekly through week 12, and then at weeks 13, 17, 21, 26, 34, 42, 52, and 65, at the time of suspected TB treatment failure, TB recurrence, or poor treatment response, and at the early treatment or study discontinuation visit.

If you are in Arm C, this procedure will be done at screening, entry, and at weeks **1,** 2, 3, and 4, and at the time of suspected TB treatment failure, TB recurrence, or poor treatment response, and at the early treatment or study discontinuation visit. Some of these collections can be done during a clinic visit or in your home. These samples will be used for TB drug susceptibility tests, to see if your TB infection responds to TB drugs.

Sputum Ribosomal RNA Synthesis (RS) Ratio (optional)
Your site may ask if you are interested in participating in the collection of sputum for RS ratio. RS ratio measures how the study treatment effects the growth of TB bacteria present in your body. If you decide to participate, you will provide additional sputum at entry, weeks 2 and 4, and at the time of suspected TB treatment failure, TB recurrence, or poor treatment response, and at the early treatment or study discontinuation visit. If you are participating in intensive PK sampling, additional sputum will be collected at weeks 1, 2, 3, and 4.

Chest X-ray
If you are in Arm 1, you will have a chest X-ray at screening, and at weeks 8 **and** 13, and at the time of suspected TB treatment failure, TB recurrence, or poor treatment response.
If you are in Arm 2, you will have a chest X-ray at screening, and at weeks 8, 13, **and** 26, and at the time of suspected TB treatment failure, TB recurrence, or poor treatment response.

If you are in Arm C, you will have a chest X-ray at screening **and at the time of suspected TB treatment failure, TB recurrence, or poor treatment response.**

ECG
You will have an ECG to look at the electrical activity of your heart.

If you are in Arm 1, this test will be done at screening, day 3, and at weeks 2, 8, **and** 13, and at the early treatment or study discontinuation visit.

If you are in Arm 2, this test will be done at screening, day 3, and at weeks 2, 8, **and** 13, and at the early treatment or study discontinuation visit.

If you are in Arm C, this test will be done at screening, day 3 and week 2.

DOT
Study staff will watch you take your study medication [*Site to insert site-specific information about directly observed therapy]*.

Quality of Life Questions
If you are in Arm 1 or Arm 2, you will be asked questions about how you feel about your quality of life, health, or other areas of your life. These questions will be asked at entry and weeks 8, 13, 26, and 65, and at the early treatment or study discontinuation visit.

Questions about Depression

You will be asked questions relating to symptoms of depression such as sadness, loss of interest, loss of appetite, problems in sleeping, thinking and concentration, as well as physical symptoms such as fatigue and weight loss. If you experience moderate depression, you will be referred to mental health services; if you experience severe depression, you will be escorted to mental health services. These will be recorded as adverse events that occurred on the study. If you are in Arm 1 or Arm 2, these questions will be asked at entry and weeks 8, 13, 26, **and** 65, and at the early treatment or study discontinuation visit.

If you are in Arm C, these questions will be asked at entry and week 8.

Questions about your experience with treatment

If you are in Arm 1 or Arm 2, you may be asked to participate in a 30 to 60-minute interview to understand more what life is like on the study treatment. You can still be in this study if you do not agree to the interview. If you agree to be interviewed, the study staff will ask you questions about your experience living with TB and to describe any challenges you face.

If you are in Arm 1, you will be asked these questions once during the study at week 13.

If you are in Arm 2, you will be asked these questions once during the study at week 26.

SIGNATURE PAGE

Intensive Pharmacokinetic (PK) sampling:

Please choose the response that matches whether you are willing to participate in the PK portion of the study by putting your initials in the space provided. Please ask the staff any questions that you have before you indicate your selection.

____ (initials) I agree to participate.

OR

____ (initials) No, I do not agree to participate.

Sputa collection for the RS ratio (if your site is participating):

Please choose the response that matches whether you are willing to provide a sputum sample for the RS ratio by putting your initials in the space provided. Please ask the staff any questions that you have before you indicate your selection.

____ (initials) I agree to participate.

OR

____ (initials) No, I do not agree to participate.

Questions about your experience with treatment

Please choose the response that matches whether you are willing to answer questions related to your quality of life, depression, and experience with treatment (qualitative interviews) by putting your initials in the space provided. Please ask the staff any questions that you have before you indicate your selection.

____ (initials) I agree to participate.

OR

____ (initials) No, I do not agree to participate.

APPENDIX I-B: SAMPLE CONSENT FORM FOR USE OF SAMPLES AND INFORMATION IN OTHER STUDIES
DIVISION OF AIDS
AIDS CLINICAL TRIALS GROUP (ACTG)

For protocol:
A5362

A5362, **FINAL**, **08Jul2022**, A Phase IIc Trial of Clofazimine- and Rifapentine-Containing Treatment Shortening Regimens in Drug-Susceptible Tuberculosis: The CLO-FAST Study

SHORT TITLE FOR THE STUDY: A5362, **FINAL Version 2.0, 08Jul2022**, Clofazimine and Rifapentine for Drug-Susceptible Tuberculosis

When samples are no longer needed for this study, the ACTG may want to use them in other studies and share them with other researchers. These samples are called “extra samples.” The ACTG will only allow your extra samples to be used in other studies if you agree to this. If you have any questions, please ask.

Identifiers will be removed from your samples and from any private information that has been collected about you. This means that no one looking at the labels or at other information will be able to know that the samples or information came from you.

Extra samples are stored in a secure central place called a repository. Your samples will be stored in the ACTG repository in the United States.

There is no limit on how long your extra samples will be stored.
[Site: Revise the previous sentence to insert limits if your regulatory authority imposes them.]

When a researcher wants to use your samples and information, their research plan must be approved by the ACTG. Also, the researcher’s institutional review board (IRB) or ethics committee (EC) will review their plan. *[Site: If review by your institution’s IRB/EC/RE is also required, insert a sentence stating this.]* IRBs/ECs protect the rights and well-being of people in research. If the research plan is approved, the ACTG will send your samples to the researcher’s location. This means that researchers who are not part of the protocol team may use your samples without asking you again for your consent.

You will not be paid for your samples. Also, a researcher may make a new scientific discovery or product based on the use of your samples. If this happens, there is no plan to share any money with you. The researcher is not likely to ever know who you are.

You may withdraw your consent for research on your extra samples at any time and the specimens will be discarded.

Please choose the response that matches what you want by putting your initials in the space provided. Please ask the staff any questions that you have before you indicate your selection.

esearch without Human Genetic Testing
If you agree, your extra samples may be stored (with usual protection of your identity) and used for ACTG-approved HIV-related research that does not include human genetic testing.

____ (initials) I understand and I agree to this storage and possible use of my samples.

OR
____ (initials) I understand but I do not agree to this storage and possible use of my samples.

Research with Human Genetic Testing

The ACTG has two different studies that collect samples for genetic testing.

If you live in the US, this study is called ACTG A5128, Plan for Obtaining Informed Consent to Use Stored Human Biological Materials (HBM) for Currently Unspecified Analyses.

If you live outside of the US, this study is called ACTG A5243, Plan for Obtaining Human Biological Samples at Non-US Clinical Research Sites for Currently Unspecified Genetic Analyses.

Your site might ask you if you would like to participate in one of these studies if it is being done where you live. If you would like to participate, you will sign a separate consent form.

Your extra samples will not be used for human genetic testing unless you sign and date a consent form for A5128 or A5243.

A5362

Primary Statistical Analysis Plan

Version 3.0

A Phase IIc Trial of Clofazimine- and Rifapentine-Containing Treatment Shortening Regimens in Drug-Susceptible Tuberculosis: The CLO-FAST Study

Protocol Version 2.0 and LOA #1

ClinicalTrials.gov Identifier: NCT04311502

13 October 2023

Created by:

Isabelle R. Weir, Jorge Leon-Cruz, and Luke Hall

Statistical and Data Analysis Center

Harvard T.H. Chan School of Public Health

Table of Contents

TABLE OF CONTENTS 1

VERSION HISTORY 3
1.1 Purpose 4

2 STUDY OVERVIEW 4
2.1 Overview of Study Design 4
2.2 Hypotheses 5
2.3 Study Objectives 5
2.3.1 Primary Objectives 6
2.3.2 Secondary Objectives 6
2.4 Overview of Sample Size Considerations 7
2.5 Overview of Formal Interim Monitoring 7

3 OUTCOME MEASURES 8
3.1 Primary Outcome Measures 8
3.1.1 Early Efficacy 8
3.1.2 Safety 8
3.2 Secondary Outcome Measures 8
3.2.1 Composite Efficacy........ 8
3.2.2 Tolerability 10
3.2.3 QT interval 10
3.2.4 Time to stable culture conversion in liquid and solid media through week 65 ... 10
3.2.5 Proportion of participants with culture conversion at weeks 8 and 12 across all study arms. 10
3.2.6 Time (days) to positivity in liquid culture (MGIT) after start of treatment through week 65........ 10
3.2.7 Change in chest X-ray (CXR) score from baseline to end of treatment (EOT). . 10
3.2.8 Proportion of participants with one or more serious adverse events (SAEs) through week 65 11
3.2.9 Proportion of participants who have a TB relapse from EOT until week 65 11
3.2.10 Proportion of participants who have a TB recurrence from EOT until week 65 . 11
3.2.11 Plasma pharmacokinetic parameters for CFZ 11
3.2.12 Change from baseline in skin pigmentation (colorimetric L*, a*, b* parameters) utilizing ambient light-independent reflectance mapping at weeks 8, 13, 26, and 65. 12
3.2.13 Change from baseline in participant-reported changes in skin pigment and distress related to perceived skin hyperpigmentation at weeks 8, 13, 26, and 65. 12

4 STATISTICAL PRINCIPLES 12
4.1 General Considerations 12
4.1.1 Analysis set definitions 13
4.1.2 Subgroups of interest 13
4.1.3 Interim Monitoring........ 13

5 ANALYSIS APPROACHES 15
5.1.1 Primary Estimands 15
5.1.2 Secondary Estimands 18
5.1.3 Secondary Objectives 22

6 PRIMARY ANALYSIS REPORT CONTENTS 26
6.1 Study Entry 26
6.2 Baseline Characteristics 26
6.3 Study Status 26
6.4 Treatment Status 26
6.5 Adverse Events 26
6.6 Primary Outcome Measures 27
6.7 Secondary Outcome Measures 27

7 ASSOCIATED DOCUMENTS 28

Version History

Version	Changes Made	Date Finalized
1	Original Version (protocol v1.0, LOA #1, LOA #2)	6/2/2021
1.1	Minor version update for LOA #3. No changes necessary.	February 1, 2022
2.0	Revised to align with protocol v2.0 and CM #1.	January 10, 2023
3.0	• Revised to align with protocol v2.0 and LOA #1<br>• Details added to reflect early close to accrual and data that will contribute to the primary analysis report<br>• Revisions to analysis specifications for time to event outcomes<br>• Additional details added for some outcome measures for clarity<br>• Edits throughout to remove redundant or duplicated information<br>• Updated writing team members and statisticians	October 13, 2023

Introduction

1.1 Purpose

This Primary Statistical Analysis Plan (SAP) describes the primary and key secondary estimands and other outcome measures that will address specific study objectives and interim monitoring of the A5362 study. The Primary SAP includes general approaches for all primary estimands, key secondary estimands, and other outcome measures in the primary manuscript(s) or submitted to ClinicalTrials.gov (regardless of the reporting timeline). The Primary SAP facilitates discussion of the statistical analysis components among the lead study investigators and statisticians, helping them agree on the statistical analyses to be performed and presented in the primary analysis report.

Detailed outlines of tables, figures, and coding descriptions that will be included in the primary analysis report are included in the Analysis Implementation Plan (AIP). Outlines of analyses for the exploratory objectives and exploratory outcome measures (which we do not describe in the Primary SAP) are provided in a separate SAP.

2 Study Overview

2.1 Overview of Study Design

A5362 is an open-label, randomized phase IIc study to evaluate whether a 3-month regimen of rifapentine/isoniazid/pyrazinamide/ethambutol (PHZE) with clofazimine (CFZ) dosed as 100 mg

daily with a 2-week 300 mg daily loading dose is superior to the standard of care (SOC) 6-month regimen of rifampin/isoniazid/pyrazinamide/ethambutol (RHZE) for DS-TB with respect to (1) early bacteriologic efficacy defined as time to culture conversion by 12 weeks post-randomization and (2) safety over 65 weeks.

Participants will be randomized to one of three treatment arms; Arm 1 with treatment for 13 weeks (including a 2-week CFZ loading dose of 300 mg daily; Arm 2 with treatment for 26 weeks; or Arm C with treatment for 4 weeks with an experimental regimen and the remaining 22 weeks with SOC (PK subgroup)).

- Arm 1 (Experimental): PHZE + CFZ 300 mg once daily for 2 weeks, PHZE + CFZ 100 mg once daily for 6 weeks; then PHZ + CFZ 100 mg once daily for 5 weeks
- Arm 2 (SOC): RHZE for 8 weeks; then RH for 18 weeks
- Arm C (PK subgroup): PHZE + CFZ 100 mg once daily for 4 weeks; then on study, off study medications and treated according to SOC (RHZE for 4 weeks; RH for 18 weeks)

We will follow all participants in Arms 1, 2, and C from randomization to week 65 for the primary and secondary objectives. Protocol v2.0 LOA #1 also includes extended follow-up for some participants corresponding to new exploratory objectives which will be detailed in a separate SAP.

Participants who consent to intensive PK sampling visits will be randomized to Arms 1, 2, or C in a 2:1:1 ratio, respectively, while participants who do not consent to intensive PK sampling will be randomized only to Arms 1 or 2 in a 2:1 ratio, respectively. We will stratify randomization based on HIV status and the presence of advanced disease as determined by chest X-ray. We will balance randomization by institution.

The primary efficacy analysis will be a superiority comparison of time to liquid culture conversion through 12 weeks in Arm 1 compared with Arm 2. The primary safety analysis will estimate and compare the proportions of participants who have a Grade 3 or higher safety event that is at least one grade higher than baseline at any time during the study. Important secondary analyses include 65-week favorable composite efficacy outcome, including cumulative relapse proportion versus 6-month SOC controls; and safety and tolerability (regimen discontinuation) in Arms 1 and 2.

The primary completion date for A5362 is the final visit date that is included in the primary safety analysis, which will occur approximately 65 weeks after the last participant enrolls. With the early closure to accrual in June 2023, the primary analysis report forming the basis of the primary manuscript will occur earlier than the original planned timepoint. We will prepare the primary analysis report using data collected at visits up to and including the date when the last enrolled participant completes the week 26 study visit. We will later conduct a supplemental analysis using data through week 65, which will form the basis of a secondary manuscript and the clinicaltrials.gov requirements.

2.2 Hypotheses

A 3-month regimen of rifapentine/isoniazid/pyrazinamide/ethambutol (PHZE) with clofazimine (CFZ) dosed as 100 mg daily with a 2-week 300 mg daily loading dose (Arm 1) will demonstrate early efficacy (time to 12-week liquid culture conversion) relative to standard of care (SOC) (RHZE; Arm 2), and will have acceptable safety and tolerability.

2.3 Study Objectives

This Primary SAP addresses the following primary and secondary objectives listed in the study protocol. We address other study objectives outlined in the protocol in subsequent secondary analysis plans.

We will analyze the primary efficacy objective below (2.3.1.1) using a superiority framework based on a one-sided 0.05 alpha level test. For analyses of other objectives, we will present estimates of the parameter of interest alongside their respective two-sided confidence intervals, as specified in Section 5.

We will finalize all analyses for the primary analysis report once the last enrolled participant has completed the week 26 study visit and all queries have been resolved.

2.3.1 Primary Objectives

2.3.1.1 To compare time to 12-week liquid culture conversion for Arm 1 with Arm 2 (SOC controls).

2.3.1.2 To compare the proportion of adverse events (safety) over 65 weeks between Arm 1 and Arm 2 (SOC controls).

2.3.2 Secondary Objectives

Each is a comparison between Arm 1 and Arm 2 unless otherwise specified.

2.3.2.1 To compare proportion of participants who experience a favorable composite efficacy outcome at 65 weeks between Arm 1 and Arm 2 participants

2.3.2.2 To compare premature regimen discontinuation (tolerability) between Arm 1 and Arm 2 participants

2.3.2.3 To estimate and compare CFZ-associated increases in QT interval (QTcF, using the Fridericia correction)

2.3.2.4 To estimate and compare time to stable liquid and solid mycobacterial culture conversion

2.3.2.5 To compare proportion of participants with liquid and solid culture conversion at weeks 8 and 12 between Arm 1 and Arm 2 participants

2.3.2.6 To compare days to positivity in automated liquid mycobacterial culture over treatment time

2.3.2.7 To compare chest radiographic score (with specific attention to resolution of cavitary disease) between Arms from baseline to end of treatment (Arm 1, week 13; Arm 2, week 26) between Arm 1 and Arm 2 participants

2.3.2.8 To compare proportions of participants who experience a favorable clinical/bacteriologic treatment outcome at 65 weeks between Arm 1 and Arm 2 participants

2.3.2.9 To estimate cumulative relapse and recurrence proportions up to week 65 for participants on Arm 1 and Arm 2

2.3.2.10 To determine CFZ pharmacokinetic (PK) parameters in plasma in Arm 1

2.3.2.11 To compare PK parameters when CFZ is given with a loading dose in Arm 1 and without a loading dose in Arm C

2.3.2.12 To objectively and subjectively assess CFZ-associated skin hyperpigmentation between Arm 1 and Arm 2 participants

2.4 Overview of Sample Size Considerations

We plan to enroll a total of 185 participants; approximately 110 in Arm 1, 55 in Arm 2, and 20 in Arm C. We estimate that we will have at least 90% power to detect an effect between Arm 1 and Arm 2 with respect to the primary efficacy outcome. We assume 70% culture conversion by week 12 in Arm 2 and a hazard ratio of 1.8. We use a 2:1 allocation ratio and assume that 10% of participants will have unevaluable culture conversion results.

For the primary safety outcome, we will compare proportions of participants with any Grade 3 or higher adverse events (AE) between Arms. Table 10.4-2 from the study protocol (copied below) gives the power we would have to detect that Arm 1 is inferior to Arm 2 assuming a two-sided type 1 error of 0.10, 10% probability of developing an AE in the SOC Arm, and varying probabilities (15-30%) of developing an AE in experimental Arm. We also provide the 90% confidence interval (CI) width for the comparison.

Protocol Table 10.4-2

Power	N1	N2	N	Arm 1 Proportion with ≥Grade 3 AE	Arm 2 Proportion with ≥ Grade 3 AE	Two-sided type 1 error	90% CI Width
0.13	100	50	150	0.15	0.10	0.10	0.210
0.36	100	50	150	0.20	0.10	0.10	0.218
0.64	100	50	150	0.25	0.10	0.10	0.225
0.85	100	50	150	0.30	0.10	0.10	0.230

2.5 Overview of Formal Interim Monitoring

The Data and Safety Monitoring Board (DSMB) will perform an initial review of the study between 4-6 months after the first enrollment, and thereafter at least every 6 months or on another schedule requested by the DSMB. For all reviews, we will provide the DSMB with detailed information on safety, tolerability, and administrative aspects (including accrual, retention, and compliance with study requirements).

There was one planned interim analysis for futility of the primary efficacy outcome using a conditional power approach. This analysis was to be conducted once 50% of participants had reached week 12 of follow-up. However, due to the early close to accrual, this interim analysis will not be completed.

3 Outcome Measures

3.1 Primary Outcome Measures

3.1.1 Early Efficacy

Time to stable culture conversion in liquid media, defined as the first of two (consecutive or non-consecutive) negative sputum cultures without an intervening positive culture, and/or visits wherein the participant is unable to produce sputum and has no signs of active TB, up to 12 weeks post-randomization.

3.1.2 Safety

Proportion across study arms experiencing any Grade 3 or higher AE that is at least a one-grade increase from baseline over 65 weeks

3.2 Secondary Outcome Measures

3.2.1 Composite Efficacy

A: Proportion with favorable clinical/bacteriologic outcome at 65 weeks post randomization

B: Proportion with favorable composite outcome including treatment completion at 65 weeks post-randomization.

Favorable, unfavorable, and unevaluable clinical/bacteriological outcomes (A) are defined as follows:

Favorable Outcome (any one of the following scenarios)

- Participants with liquid culture negative status at week 65.
- Participants without signs or symptoms of ongoing active TB and are unable to produce a sputum specimen at 65 weeks.
- Participants who at the end of the follow-up period are clinically without symptoms/signs of ongoing active TB and produce a sputum specimen that is contaminated in two liquid cultures without evidence of TB.

For any of the above to be considered favorable, participants must not already have been considered unfavorable, as defined below. Unfavorable Outcome

- Absence of cure. A participant will be considered to have absence of bacteriological cure if a sputum sample obtained at or after end of treatment for Arm 1 (week 13), or at or after week 17 for Arm 2 is culture-positive in liquid or solid media for an *Mtb* strain genotypically matched with the initial isolate. A second sputum sample, obtained at least four hours following the first sputum collection (see section 6.2.3 of protocol), is required to confirm absence of bacteriological cure.
- Participants who die from any cause during study treatment or follow-up except from violent or accidental cause (e.g., road traffic accident).
- Participants who had a positive culture for *Mtb* when last seen, whether confirmed by a second sample or not, unless determined to have been reinfected.

- Participants experiencing extension of treatment beyond the nominal level (week 13 for Arm 1 and week 26 for Arm 2) due to clinically inadequate response. These cases should be managed individually in conjunction with and after having notified the A5362 Clinical Management Committee (CMC). Extension of treatment to make up missed doses will not count as unfavorable.

<u>Unevaluable Outcome</u>

- Participants lost to follow-up during treatment or post-treatment follow-up with their last culture being negative for *Mtb*.
- Violent or accidental death.
- Participants with recurrent TB due to a new strain of *Mtb* confirmed by conventional molecular genotyping (as defined by MIRU and IS6110 typing).
- Women who become pregnant during their assigned active treatment and stop their assigned treatment.

Favorable, unfavorable, and unevaluable composite outcomes (B) are defined as follows:

<u>Favorable Outcome</u>
Same as in outcome measure 3.2.1 A.

<u>Unfavorable Outcome</u>
Same as in outcome measure 3.2.1 A, and:

- Participants lost to follow-up during treatment phase of each Arm (i.e., 13 weeks and 26 weeks for Arms 1 and 2, respectively).
- Participants failing to complete treatment (see below for Definitions of Adequate Treatment) and not assessable at the end of the follow-up period.
- Participants receiving any one or more of the following:
  - extension of treatment beyond the nominal level, except to make up missed doses
  - a re-start of treatment following ≥30 consecutive days lost to follow-up
  - a change in at least one drug in treatment regimen for any reason except re-infection, pregnancy, or temporary drug challenge.

<u>Unevaluable Outcome</u>

- Participants lost to follow-up after treatment phase, and not assessable at the end of follow-up, with their last culture being negative for *Mtb.*
- Violent or accidental death.
- Participants re-infected with a new strain of *Mtb* confirmed by conventional molecular genotyping (as defined by MIRU and IS6110 typing).
- Women who become pregnant during their assigned active treatment and stop their assigned treatment.

<u>Definitions of Adequate Treatment Within 3-month Regimen (Arm 1)</u>

- Taken a total of at least 56 doses of allocated intensive phase treatment within 70 days of starting treatment
- AND taken at least 28 doses of continuation phase treatment within 49 days (7 weeks) of completion of the intensive phase
- AND missed no more than 21 doses of medication overall.

Definitions of Adequate Treatment within 6-month Regimen (Arm 2)

- Taken a total of at least 56 doses of allocated intensive phase treatment within 70 days of starting treatment
- AND taken at least 100 doses of continuation phase treatment within 154 days (22 weeks) of completion of the intensive phase
- AND missed no more than 42 doses of medication overall.

3.2.2 Tolerability

Proportion with premature discontinuation through 65 weeks, defined as discontinuation other than due to violent death, natural disaster, or administrative censoring.

3.2.3 QT interval

We derive the outcome measures defined below from ECG readings, which the site conducts in triplicate (three ECGs 5-10 minutes apart). We will use the mean of all measurements (up to 3) that are readable and available.

3.2.3.1 Mean baseline QTcF and change from baseline in mean QTcF at weeks 2, 8, and end of treatment (EOT) (Arm 1, week 13; Arm 2, week 26)

3.2.3.2 Occurrence of absolute QTcF ≥480 ms and <500 ms, and ≥500 ms, at any time during study treatment

3.2.3.3 Occurrence of QTcF change from baseline of ≥30 ms and <60 ms, and ≥60 ms, at any time during study treatment

3.2.4 Time to stable culture conversion in liquid and solid media through week 65

Defined as the first of two (consecutive or non-consecutive) negative sputum cultures without an intervening positive culture, and/or visits wherein the participant is unable to produce sputum and has no signs of active TB. Conducted separately for liquid and solid media.

3.2.5 Proportion of participants with culture conversion at weeks 8 and 12 across all study arms.

Liquid (MGIT) and solid media analyzed separately.

3.2.6 Time (days) to positivity in liquid culture (MGIT) after start of treatment through week 65

We will report the median (Q1, Q3) times to positivity in liquid culture at each time point (weeks 1, 2, 3, 4, 6, 8, 10, and 12) in Arm 1 and Arm 2.

3.2.7 Change in chest X-ray (CXR) score from baseline to end of treatment (EOT).

Sites conduct the baseline chest X-ray during screening. We will compute the chest X-ray score with the following validated formula (Ralph et al. Thorax 2010):

CXR Score = percent of total lung affected by any pathology + 40 (if cavitation is present).

We will compute the CXR scores based on both site assessment of the chest Xray and an assessment by independent central reviewers blinded to study treatment.

3.2.8 Proportion of participants with one or more serious adverse events (SAEs) through week 65

3.2.9 Proportion of participants who have a TB relapse from EOT until week 65

For participants who had successful culture conversion through the end of study treatment, TB relapse is defined as a recurrence of TB emanating from the same strain as the participant's originally diagnosed TB, which will be determined through molecular genotyping.

3.2.10 Proportion of participants who have a TB recurrence from EOT until week 65

3.2.11 Plasma pharmacokinetic parameters for CFZ

Arm 1

We will report the minimum concentration (Cmin), maximum concentration (Cmax), time of Cmax (Tmax), and area under the concentration curve (AUC024h) estimated though non-compartmental analysis for weeks 2 and 13 summarized separately.

Arm C

We will report the estimated minimum concentration (Cmin), maximum concentration (Cmax), time of Cmax (Tmax), and area under the concentration curve (AUC0-24h) estimated though non-compartmental analysis for week 2.

Arm 1 versus Arm C

We will compare the estimated minimum concentration (Cmin), maximum concentration (Cmax), time of Cmax (Tmax), and area under the concentration curve (AUC0-24h) at week 2 between Arm 1 and Arm C intensive PK participants. We will estimate the geometric mean ratio comparing Arm 1 to Arm C and corresponding 90% confidence interval. As a supplemental analysis, we will use a Wilcoxon rank sum test and report the p-value.

3.2.12 Change from baseline in skin pigmentation (colorimetric L*, a*, b* parameters) utilizing ambient light-independent reflectance mapping at weeks 8, 13, 26, and 65.

Mean change in total color difference from week 0 to each of weeks 8, 13, 26, 65 in Arm 1 vs Arm 2 at each of the following locations; (1) left cheek, (2) right cheek, (3) forehead, (4) chin.

Mean change in total color difference from week 0 to each of weeks 8, 13, 26, 65 in Arm 1 vs Arm 2 using, for each participant, the location with the maximum change.

3.2.13 Change from baseline in participant-reported changes in skin pigment and distress related to perceived skin hyperpigmentation at weeks 8, 13, 26, and 65.

Mean subjective 'change in coloration' score between week 0 and weeks 8, 13, 26, and 65 in Arm 1 vs Arm 2 (10-point numeric rating scale where 0=none, 10=most significant possible).

Mean subjective 'distress related to skin coloration' score at each of weeks 0, 8, 13, 26, and 65 in Arm 1 vs Arm 2 (10-point numeric rating scale where 0=none, 10=worst possible)

4 Statistical Principles

4.1 General Considerations

All statistical comparisons described in Section 3 are between Arms 1 and 2, unless otherwise noted. The analysis set or study population is specified for the analysis of each study objective. We explicitly define these in the next section.

For time-to-event analyses, we will base each participant's follow-up time from the date of randomization. We will compute the time to event as the weeks from randomization to the study visit week when the event occurred, not the actual date of the visit. Similarly, participants who do not have an event by the last scheduled visit and are not lost to follow-up are censored on the respective study visit week, not the actual date of their last visit. This ensures there are not major artificial fluctuations in the risk set near the end of the period for the outcomes in which we estimate Kaplan-Meier proportions at a fixed time point.

For analyses where we estimate Kaplan-Meier proportions at a fixed time point, we will use Greenwood's estimator to estimate the variance of the proportions in each arm, and the variance for the difference in proportions between arms will be computed as the sum of the squared variance estimates for Arms 1 and 2.

4.1.1 **Analysis set definitions**

4.1.1.1 **Analysis sets (used for primary (5.1.1) and key secondary (5.1.2) estimands):**

- Efficacy Set:
  All participants who are randomized to study treatment and are not late exclusions. (Participants will be late exclusions if they do not have any positive culture (liquid or solid) at screening, entry, or week 1, or if they have TB that is RIF-resistant or INH-resistant identified at screening or entry.)

- Safety Set:
  All participants who start assigned study treatment.

- Start treatment/not late exclusion set:
  All participants who start assigned study treatment and are not late exclusions.

- Alive/adequate treatment set:
  All participants who complete an adequate course of treatment as defined within Sections 10.2.5 and 10.2.6 of the protocol and who do not die during treatment.

- Per protocol Set:
  All participants who start and complete assigned study treatment.

4.1.2 Subgroups of interest

Subgroups defined by levels of sex
Subgroups defined by levels of race/ethnicity
Subgroups defined by levels of HIV status
Subgroups defined by presence (or absence) of advanced disease on chest x-ray conducted at screening.

4.1.3 Interim Monitoring

4.1.3.1 Early Assessment of QT Prolongation

After 12 Arm 1 participants have evaluable AE data through week 2, we will find the point estimate of the proportion of participants in the ITT population on Arm 1 with prolonged QT interval (defined by QTcF > 500 ms) and corresponding two-sided Pearson-Klopper exact 95% confidence interval.

4.1.3.2 Interim Futility Analysis

Note: Due to the early closure to accrual, this analysis will not be conducted.

We will estimate the conditional power of the test statistic at the second DSMB review using two methods in the efficacy set.

1) Under the assumption that the future data follows the hypothesized difference between Arm 1 and Arm 2 (hazard ratio of 1.8 between arms).

2) Under the assumption that future data follows the estimated difference between Arm 1 and Arm 2 at the time of the review (the estimated hazard ratio).

4.1.3.3 Unfavorable Composite Outcome Monitoring in Arm 1

We will find the two-sided 95% confidence bands for cumulative unfavorable outcome probabilities in Arm 1 in the safety set approximately one month after the first participant completes treatment, and will continue approximately monthly and for each DSMB review until the end of the study. We will present these estimates alongside historical unfavorable proportions for SOC on the REMox, OFLOTUB, RIFAQUIN, and Study31 trials, and A5362 SOC data as available. There will be no adjustment to the confidence bands for multiple looks.

4.1.3.4 Early Recurrence Monitoring in Arm 1

We will find the two-sided 95% confidence bands for cumulative relapse probabilities in Arm 1 beginning approximately 2 months after the first participant completes treatment, and will continue approximately monthly and for each DSMB review until the end of the study. We will present these results alongside historical relapse proportions for SOC on the REMox, OFLOTUB, and RIFAQUIN trials, and A5362 SOC data as available. There will be no adjustment to the confidence bands for multiple looks.

5 Analysis Approaches

5.1.1 Primary Estimands

<table>
<tr><th colspan="3">Primary Objective 2.3.1.1: To compare time to 12-week liquid culture conversion for Arm 1 with SOC controls (Arm 2)</th></tr>
<tr><td>Estimand description</td><td colspan="2">Time to liquid culture conversion through 12 weeks of follow-up among individuals aged ≥18 years and with pulmonary TB without demonstrated isoniazid or rifampicin resistance.</td></tr>
<tr><td>Treatment</td><td colspan="2">13-week Experimental Regimen:
rifapentine/isoniazid/pyrazinamide/ethambutol (PHZE) + clofazimine (CFZ)

26-week Standard of Care: rifampicin/isoniazid/pyrazinamide/ethambutol (RHZE) followed by rifampicin/isoniazid (RH)</td></tr>
<tr><th colspan="2">Target population</th><th>Analysis set</th></tr>
<tr><td colspan="2">Individuals aged ≥18 years living with confirmed pulmonary TB without demonstrated isoniazid or rifampicin resistance.</td><td>Efficacy set (All participants who are randomized to study treatment and are not late exclusions)</td></tr>
<tr><th colspan="2">Variable</th><th>Outcome measure 3.1.1</th></tr>
<tr><td colspan="2">Time to stable culture conversion based on sputum samples taken at serial collections up to 13 weeks after treatment initiation.

Note: The time of stable culture conversion will be the time of the sampling date of the first of two culture negative sputum samples in liquid media (MGIT) without an intervening positive culture. We also consider a participant's inability to produce sputum with no signs of active TB at a given visit as a negative sputum culture. A negative culture at week 13 can be used as the confirmatory second culture to determine stable culture conversion at week 12.</td><td>Time to stable culture conversion in liquid media, defined as the first of two (consecutive or nonconsecutive) negative sputum cultures without an intervening positive culture, and/or visits wherein the participant is unable to produce sputum and has no signs of active TB, up to 12 weeks post-randomization.</td></tr>
<tr><th colspan="2">Handling of intercurrent events</th><th>Handling of missing data</th></tr>
<tr><td colspan="2">The following intercurrent events are relevant to the estimand:

1. Failure to start TB treatment
Observations will be used to determine the variable (treatment policy strategy)

2. Premature treatment discontinuation due to suspected TB treatment failure or TB recurrence or poor treatment response.
Observations will be used to determine the variable (treatment policy strategy)

3. Premature TB treatment discontinuation due to TB death
Observations will be used to determine the variable (treatment policy strategy)

4. Premature treatment discontinuation for other reason
Observations will be used to determine the variable (treatment policy strategy)

5. Non-TB death</td><td>Participants who have contaminated or missing samples leading up to and including week 12 will be censored at the last sampling date for which they had a valid sputum culture assessment.

Participants who experience premature TB treatment discontinuation due to TB death will be censored at week 12.</td></tr>
</table>

Observations will be used to determine the variable (treatment policy strategy)	
**Population-level summary measure**	**Analysis approach**
Hazard ratio of culture conversion through 12 weeks of follow-up.	We will estimate the treatment effect with a hazard ratio and corresponding 90% two-sided confidence interval. We consider Arm 1 superior to Arm 2 if the one-sided p-value is less than 0.05 in favor of Arm 1. The hazard ratio (HR) will be estimated from a Cox proportional-hazards model, with HIV status (positive; negative) and advanced disease according to chest X-ray (yes/no) treated as stratification factors. We will assess the proportional hazards assumption visually by plotting the scaled Schoenfeld residuals against the natural logarithm of the time to event. In the event that the data violate the proportional hazards assumption, we will conduct the primary analysis with a stratified logrank test.<br><br>Participants who do not achieve stable culture conversion, but who are on study and have contaminated or missing cultures leading up to and including week 12 will be censored at the last sampling visit for which they had a valid sputum culture assessment. Participants who are lost to follow-up prior to 12 weeks with their last culture being positive will be censored at 12 weeks (i.e. assumed they did not have a culture conversion by week 12), and participants who are lost to follow-up prior to 12 weeks with their last culture being negative will be censored at the last sampling visit for which they had a valid culture result. Participants who die prior to week 12 will be censored at 12 weeks.

Sensitivity Analyses

None.

Supplemental Analyses

We will also test the treatment by sex and treatment by race/ethnicity interaction terms in separate Cox proportional hazards models. These models will also include the same stratification factors as the primary analysis: HIV status and advanced disease according to chest X-ray reading. In a supplemental analysis, we will estimate the difference in restricted mean survival times (difference in restricted mean time to culture conversion) for a time horizon of 12 weeks.

<table>
<tr><th colspan="3">Primary Objective 2.3.1.2: To compare the proportion of adverse events (safety) over 65 weeks between Arm 1 and Arm 2</th></tr>
<tr><td>Estimand description</td><td colspan="2">Probability of having at least one grade 3 or higher adverse event through 65 weeks that is at least a one-grade increase from baseline among patients aged ≥18 years living with pulmonary TB without demonstrated isoniazid or rifampicin resistance.</td></tr>
<tr><td>Treatment</td><td colspan="2">13-week Experimental Regimen:
rifapentine/isoniazid/pyrazinamide/ethambutol (PHZE) + clofazimine (CFZ)

26-week Standard of Care: rifampicin/isoniazid/pyrazinamide/ethambutol (RHZE) followed by rifampicin/isoniazid (RH)</td></tr>
<tr><th colspan="2">Target population</th><th>Analysis set</th></tr>
<tr><td colspan="2">Patients aged ≥18 years living with pulmonary TB without demonstrated isoniazid or rifampicin resistance who initiate TB therapy.</td><td>Safety Set (All participants who start their assigned study treatment)</td></tr>
<tr><th colspan="2">Variable(s)</th><th>Outcome measure 3.1.2</th></tr>
<tr><td colspan="2">Occurrence of a Grade 3 or higher adverse event through 65 weeks that is new in onset or aggravated in severity of frequency from the baseline condition.</td><td>Proportion across study arms experiencing any Grade 3 or higher AE that is at least a one-grade increase from baseline over 65 weeks.</td></tr>
<tr><th colspan="2">Handling of intercurrent events</th><th>Handling of missing data</th></tr>
<tr><td colspan="2">1. Persistent clinical disease and extension of treatment
Observations will be used to determine the variable (treatment policy strategy)

2. Premature treatment discontinuation
Observations will be used to determine the variable (treatment policy strategy)

3. Premature treatment discontinuation due to suspected TB treatment failure or TB recurrence or poor treatment response. Observations will be used to determine the variable (treatment policy strategy)</td><td>Participants who discontinue follow-up before week 65 will have their outcome determined based on data available until the time of discontinuation (i.e., missing observations after discontinuation that would be relevant to the variable are considered missing completely at random).

Sensitivity analyses will consider participants who discontinue follow-up before week 65 as having at least one Grade 3 or higher adverse event that is at least a one-grade increase from baseline.</td></tr>
<tr><th colspan="2">Population-level summary measure</th><th>Analysis approach</th></tr>
<tr><td colspan="2">Probability of having at least one Grade 3 or higher AE that is at least one-grade increase from baseline at any time during the 65 weeks after treatment initiation.</td><td>We will compare the cumulative proportion of participants experiencing a Grade 3 or higher AE that is at least one-grade increase from baseline at any time during the 65-week study period between Arm 1 and Arm 2 using the KaplanMeier estimator at week 65. Participants who have not experienced an event will be censored at the last completed study visit. For participants who experience more than one event, we will use the time of the first occurrence as the time of event. We will present the point estimate and 90% two-sided confidence intervals for the difference in cumulative proportions.</td></tr>
</table>

Supplemental Analyses:

As supplementary analyses, we will also estimate the Kaplan-Meier proportions and confidence intervals for the outcome measure up to week 13, and up to week 26. These time horizons correspond to the end of treatment for Arm 1, and Arm 2, respectively.

5.1.2 **Secondary Estimands**

<table>
<tr><th colspan="3">Secondary Objective 2.3.2.1:
To compare proportions of participants treated with RPT/CFZ-containing regimens who experience favorable composite efficacy outcome at 65 weeks with Arm 2 (6-month SOC controls)</th></tr>
<tr><td>Estimand description</td><td colspan="2">Difference in proportion with favorable outcomes 65 weeks after initiation of TB therapy (PHZE+CFZ versus RHZE) among individuals aged ≥18 years and with pulmonary TB without demonstrated isoniazid or rifampicin resistance.</td></tr>
<tr><td>Treatment</td><td colspan="2">13-week Experimental Regimen:
rifapentine/isoniazid/pyrazinamide/ethambutol (PHZE) + clofazimine (CFZ)

26-week Standard of Care: rifampicin/isoniazid/pyrazinamide/ethambutol (RHZE) followed by rifampicin/isoniazid (RH)</td></tr>
<tr><th colspan="2">Target population</th><th>Analysis set</th></tr>
<tr><td colspan="2">Individuals aged ≥18 years with confirmed pulmonary TB without demonstrated isoniazid or rifampicin resistance who initiate TB therapy.</td><td>Efficacy set (All participants who are randomized to study treatment and are not late exclusions)</td></tr>
<tr><th colspan="2">Variables</th><th>Outcome measure 3.2.1 A</th></tr>
<tr><td colspan="2">Favorable outcome 65 weeks after initiation of TB therapy following evidence of cure at treatment completion where favorable outcome is defined by any one of the following:

• Liquid culture negative at 65 weeks after TB therapy initiation

• No signs or symptoms of ongoing active TB and unable to produce a sputum specimen at 65 weeks after TB therapy initiation

• No signs or symptoms of ongoing active TB and a sputum specimen that is contaminated in two liquid cultures without evidence of TB at 65 weeks after TB therapy initiation

Absence of cure at or after treatment completion is an unfavorable outcome.</td><td>Favorable clinical/bacteriologic outcome at the 65 week study visit.</td></tr>
<tr><th colspan="2">Handling of intercurrent events</th><th>Handling of missing data</th></tr>
<tr><td colspan="2">The following intercurrent events are relevant to the estimand.

1. Death except from violent or accidental cause (e.g., road traffic accident)
Unfavorable outcome (composite strategy)

2. Violent or accidental death
Unevaluable outcome* (hypothetical strategy)

3. Extension of treatment beyond the nominal duration due to clinically inadequate response Unfavorable outcome (composite strategy)

4. New TB diagnosis due to a new strain of Mtb
Unevaluable outcome* (hypothetical strategy)</td><td>Participants lost to follow-up and culture negative at their last visit meet the definition of “unevaluable” and will be censored at the time of their last follow-up visit

Participants with a positive culture for Mtb when last seen, whether confirmed by a second sample or not, unless determined to have been re-infected will be considered to have an unfavorable outcome and the event time will be the visit when last seen.

Participants with a new TB diagnosis due to a new strain of Mtb will be censored at the time of their last follow-up visit.</td></tr>
</table>

5. Pregnancy during treatment resulting in discontinuation of initial therapy<br>Unevaluable outcome* *(hypothetical strategy)*<br><br>6. Failure to complete treatment (except for 2 and 5 above)<br>*All follow-up through to 65 weeks will be used to determine the variable irrespective of duration of treatment (treatment policy strategy)*<br><br>* See handling of missing data for imputation rules for unevaluable outcomes	Participants who become pregnant during assigned active treatment and discontinue treatment will be censored at the time of treatment discontinuation.
Population-level summary measure	Analysis approach
Difference in cumulative probability of having a favorable clinical/bacteriologic outcome at 65 weeks after TB therapy initiation.	Difference in cumulative proportion of participants at 65 week visit together with 90% and 95% two-sided confidence intervals. We will use the Kaplan-Meier estimator and Greenwood's estimate for the standard error.

<table>
<tr><td colspan="3">Secondary Objective 2.3.2.1:
To compare proportions of participants treated with RPT/CFZ-containing regimens who experience favorable composite efficacy outcome at 65 weeks with Arm 2 (6-month SOC controls)</td></tr>
<tr><td>Estimand description</td><td colspan="2">Difference in proportion favorable outcomes 65 weeks after initiation of TB therapy (PHZE+CFZ versus RHZE) among individuals aged ≥18 years and with pulmonary TB without demonstrated isoniazid or rifampicin resistance who complete TB treatment.</td></tr>
<tr><td>Treatment</td><td colspan="2">13-week Experimental Regimen:
rifapentine/isoniazid/pyrazinamide/ethambutol (PHZE) + clofazimine (CFZ)

26-week Standard of Care: rifampicin/isoniazid/pyrazinamide/ethambutol (RHZE) followed by rifampicin/isoniazid (RH)</td></tr>
<tr><td colspan="2">Target population</td><td>Analysis set</td></tr>
<tr><td colspan="2">Individuals aged ≥18 years living with confirmed pulmonary TB without demonstrated isoniazid or rifampicin resistance who complete TB therapy.</td><td>Efficacy set (All participants who are randomized to study treatment and are not late exclusions)</td></tr>
<tr><td colspan="2">Variables</td><td>Outcome measure 3.2.1B</td></tr>
<tr><td colspan="2">Favorable outcome 65 weeks after initiation of TB therapy following evidence of cure at treatment completion where favorable outcome is defined by any one of the following:

• Liquid culture negative at 65 weeks after TB therapy initiation

• No signs or symptoms of ongoing active TB and unable to produce a sputum specimen at 65 weeks after TB therapy initiation

• No signs or symptoms of ongoing active TB and a sputum specimen that is contaminated in two liquid cultures without evidence of TB at 65 weeks after TB therapy initiation

Absence of cure at or after treatment completion is an unfavorable outcome.</td><td>Favorable clinical/bacteriologic outcome at the 65 week study visit.</td></tr>
<tr><td colspan="2">Handling of intercurrent events</td><td>Handling of missing data</td></tr>
<tr><td colspan="2">The following intercurrent events are relevant to the estimand:

Intercurrent events that occur during therapy:

1. Death except from violent or accidental cause (e.g., road traffic accident)
Unfavorable outcome* (Composite strategy)

2. Violent or accidental death
Unevaluable outcome* (Composite strategy)

3. A re-start of therapy following $\geq$ 30 consecutive days without therapy
Unfavorable outcome (Composite strategy)

4. A change in at least one drug in treatment regimen for any reason except re-infection, pregnancy, or temporary drug challenge.
Unfavorable outcome (Composite strategy)</td><td>Participants lost to follow-up during the treatment phase are considered to have an unfavorable outcome (regardless of TB culture result at the last available visit) and the event time is the time of the last visit.

Participants failing to complete treatment and not assessable at the end of the follow-up period are considered to have an unfavorable outcome and the event time is the time of the last treatment dose received.

Participants receiving an extension of treatment, re-start of treatment, or change in treatment (see full definition B of unfavorable outcome) are considered to have an unfavorable outcome and the event time is the time of the treatment modification.</td></tr>
</table>

5. Pregnancy during treatment resulting in discontinuation of initial therapy<br>Unevaluable outcome* (Composite strategy)<br><br>**Intercurrent events that occur after adequate** completion of therapy:**<br><br>6. Death except from violent or accidental cause (e.g., road traffic accident)<br>Unfavorable outcome (Composite strategy)<br><br>7. Violent or accidental death<br>Unevaluable outcome* (Hypothetical strategy)<br><br>8. Extension of treatment beyond the nominal duration except to make up missed doses<br>Unfavorable outcome (Composite strategy)<br><br>9. New TB diagnosis due to a new strain of *Mtb* Unevaluable outcome* (Hypothetical strategy)<br><br>* See handling of missing data for imputation rules for unevaluable outcomes	Participants lost to follow-up after the treatment phase with their last available TB culture being negative are considered unevaluable and are censored at the time of the last visit.<br><br>Participants who experience violent or accidental death are considered to have an unevaluable outcome and will be censored at the time of their last follow-up visit.<br><br>Participants reinfected with a new strain of Mtb are considered to have an unevaluable outcome and will be censored at the time of their last follow-up visit.<br><br>Participants who become pregnant during their assigned active treatment and who stop their assigned treatment are considered to have an unevaluable outcome and will be censored at the time of the last treatment dose received.
Population-level summary measure	Analysis approach
Difference in cumulative probability of having a favorable clinical/bacteriologic outcome at 65 weeks after TB therapy initiation .	Difference in cumulative proportion of participants at 65 week visit together with 90% and 95% two-sided confidence intervals. We will use the Kaplan-Meier estimator and Greenwood's estimate for the standard error.

** Adequate completion of therapy is determined by the following definitions:

<u>Definitions of Adequate Treatment Within 3-month Regimen (Arm 1 in the setting of this trial)</u>

- Taken a total of at least 56 doses of allocated intensive phase treatment within 70 days of starting treatment
- AND taken at least 28 doses of continuation phase treatment within 49 days (7 weeks) of completion of the intensive phase
- AND missed no more than 21 doses of medication overall.

<u>Definitions of Adequate Treatment within 6-month Regimen (Arm 2 in the setting of this trial)</u>

- Taken a total of at least 56 doses of allocated intensive phase treatment within 70 days of starting treatment
- AND taken at least 100 doses of continuation phase treatment within 154 days (22 weeks) of completion of the intensive phase
- AND missed no more than 42 doses of medication overall.

5.1.3 Secondary Objectives

5.1.3.1 Tolerability (Objective 2.3.2.2; Outcome Measure 3.2.2)

We will compare the proportion of participants who permanently and prematurely discontinue study treatment for any reason between arms in the start treatment/not late exclusion set. Permanent and premature discontinuation of study treatment is defined as not having completed an adequate dose of treatment as defined in Section 3.2.1. We will exclude participants who discontinued due to violent or accidental death, natural disaster, or administrative censoring (e.g. site closure), from the analysis. The difference in proportions will be estimated using binomial proportions, and we will provide exact 95% confidence intervals.

5.1.3.2 QT Prolongation (Objective 2.3.2.3; Outcome Measures 3.2.3.1, 3.2.3.2, 3.2.3.3)

We will compare the mean change in QTcF from screening to weeks 2, 8, and 13 (3.2.3.1) between Arm 1 and Arm 2 in the safety set using a GEE model with exchangeable correlation structure and time by treatment interaction terms (treating time as a categorical variable).

We will compare the change from baseline in QTcF over time between arms using a linear mixed effects model. Randomization arm and week (time) will be fixed effects and participant ID will be a random effect.

We will compare the odds of participants having maximum occurrence of absolute QTcF ≥ 480 ms and <500 ms, or ≥ 500 ms (3.2.3.2) between Arm 1 and Arm 2 in the safety set using a proportional odds model, if the proportional odds assumption is satisfied. We will assess the proportional odds assumption using the score test for the proportional odds assumption implemented in SAS PROC LOGISTIC. If the score test indicates a statistically significant result at the 0.05 level, we will also assess the proportional odds assumption graphically to confirm. If graphical evidence suggests the proportional odds model is valid, we will use it for analysis.

If the proportional odds assumption does not hold, we will instead estimate the difference in binomial proportions and 95% confidence intervals for the collapsed category of QTcF ≥ 480 ms (including QTcF ≥ 500 ms), and separately for the category QTcF ≥ 500 ms.

The analysis of outcome measure 3.2.3.3 will follow the same approach as that of 3.2.3.2.

5.1.3.3 Time to stable culture conversion in liquid media, and solid media at week 26 (Objective 2.3.2.4; Outcome Measure 3.2.4)

Time to stable culture conversion in liquid media will follow the same approach as described in Section 5.1.1, except we will censor participants at week 26 instead of week 12.

Time to stable culture conversion in solid media will be analyzed separately, and also follow the same approach as described in Section 5.1.1, except we will censor participants at week 26 instead of week 12.

5.1.3.4 Proportion with culture conversion at week 8 and 12 (Objective 2.3.2.5; Outcome Measure 3.2.5)

We will compare the proportion of participants with stable culture conversion by week 8 and week 12 between Arm 1 and Arm 2 in the efficacy set. We will analyze liquid and solid cultures separately. We will present the difference in proportions and exact 95% confidence intervals.

5.1.3.5 Proportion with one or more SAE (Outcome Measure 3.2.8)

We will estimate the proportion of participants with one or more SAEs of any grade between Arm 1 and Arm 2 in the safety set using the same approach as described in Section 5.1.1

As in Section 5.1.1, supplementary analyses will compare SAEs between arms up to week 13 and up to week 26.

5.1.3.6 Time to Positivity (Objective 2.3.2.6, Outcome Measure 3.2.6)

We will compare time (days) to positivity of TB cultures between Arm 1 and Arm 2 in the efficacy set separately at weeks 1, 2, 3, 4, 6, 8, 10, and 12 using Cox proportional hazards models or logrank tests. We will present the hazard ratios and 95% confidence intervals for each week.

We will include side-by-side box plots to show the distribution of time to positivity at each visit in each of Arms 1 and 2. In exploratory analyses, we may consider comparing time (days) to positivity of TB cultures between Arm 1 and Arm 2 in the efficacy set using a random effects joint model for time-to-event data with repeated measures. We may also consider other models and mechanistic approaches.

5.1.3.7 Chest X-Ray Scores (Objective 2.3.2.7; Outcome Measure 3.2.7)

Each participant will have a CXR Score computed at screening and at EOT (week 13 for Arm 1; week 26 for Arm 2). We will compute the difference in scores from baseline to EOT between arms in the efficacy set using linear regression, with adjustment for stratification factors of HIV status and advanced disease status.

We will carry out two analyses for this outcome; (1) based on the site reading of CXR, and (2) based on the central reading of CXR. The central reading will take precedence when presenting results.

5.1.3.8 Proportion of participants who have a TB relapse (Objective 2.3.2.9; Outcome Measure 3.2.8)

We will estimate the proportion of participants in Arm 1 and Arm 2 with TB relapse using KaplanMeier estimates and 95% confidence intervals at 6 months (Arm 1), 9 months (Arms 1 and 2), and 12 months post-treatment (Arm 1 only) in the start treatment/not late exclusion set. We define the time of relapse as the time from end of treatment until the first sputum sample that is culture positive in liquid or solid media for an *Mtb* strain that has matching genotype with the baseline isolate. A second positive sputum sample obtained at least 4 hours following the first sputum collection is required to confirm a relapse. If the second sputum sample is negative, this will not be counted as a relapse. If a participant is lost to follow-up without a second sputum sample confirmation of relapse (including contaminated or missing), it will be counted as a relapse.

For monitoring and interim reviews, we will estimate the proportion of participants with a confirmed relapse over time with 95% pointwise confidence bands. In addition, we will estimate the proportion of participants with a suspected TB recurrence or poor treatment response after successful culture conversion, even if relapse/recurrence has yet to be confirmed bacteriologically.

5.1.3.9 Proportion of Participants who have a TB recurrence (Objective 2.3.2.9; Outcome Measure 3.2.10)

The analysis for the proportion of participants with a TB recurrence will follow the same approach described in Section 5.1.3.8. We define recurrence in the same way as relapse, except it is not required that the TB strain has matching genotype with the baseline sample.

5.1.3.10 Plasma Pharmacokinetic Parameters for CFZ (Objectives 2.3.2.10 and 2.3.2.11; Outcome Measures 3.2.11)

Elin Svensson and team at Radboudumc, The Netherlands, will conduct this analysis.

The PK parameters of interest (Cmin, Cmax, Tmax, AUC0-24h) for CFZ in Arm 1 and Arm C of the alive/adequate treatment set will be estimated using non-compartmental methods applied to concentrations from intensive PK sampling visits. The AUC will be determined using the trapezoidal rule. Cmin will be the minimum observed concentration after the observed dose. Cmax will be the maximum observed concentration. Tmax is the time at which Cmax occurs.

<u>Arm 1 versus Arm C</u>

We will estimate the geometric mean ratio for each PK parameter (Cmin, Cmax, Tmax, AUC024h) together with the 90% confidence interval using available data in the alive/adequate treatment set.

This non-compartmental analysis will be in conjunction with non-linear mixed-effects modeling to estimate the primary PK parameters (e.g., apparent oral clearance (CL/F), volume of distribution, rate of absorption). The model-based analysis will use the intensive PK samples as well as the sparse samples collected throughout the study period and all protein concentration data. We will model the PK observations continuously over time, taking in account each participant's actual dosing history. We will conduct a covariate analysis to evaluate the potential impact of demographics and concomitant medications. We will compare the derived secondary metrics with the non-compartmental analysis results in a posterior predictive check.

5.1.3.11 CFZ-associated skin hyperpigmentation (Objective 2.3.2.12; Outcome Measures 3.2.12 and 3.2.13)

We will compute the total color difference scores using the CIELAB color space output values, (L, a, b), from the colorimeter device. Since each reading is done in triplicate, we will use the average scores from the three readings for L, A, and b for each participant. The analysis for each anatomical locations will be separate.

The CIE76 formula for total color difference, Delta E, is given by: $\Delta E = \sqrt{\Delta L^2 + \Delta a^2 + \Delta b^2}$

We will compare the mean change in total color difference (ΔE) from week 0 (entry) to each of weeks 8, 13, 26, 65 in Arm 1 vs Arm 2 at each of the following locations; (1) forehead, (2) left cheek, (3) right cheek, (4) chin in the mITT-B population. That is, for each anatomical location, we will compute the difference between ΔE at week 0 and week 8 for each of Arm 1 and Arm 2. We will compare the differences with a t-test.

We will also compare the mean change in total color difference (ΔE) from week 0 (entry) to each of weeks 8, 13, 26, 65 in Arm 1 vs Arm 2 using, for each participant in the safety set, the location with the maximum change. We will compare differences between arms with a t-test.

We will compare the 'mean change in coloration score' and 'mean distress related to skin coloration score' for Arm 1 vs Arm 2 at each of weeks 0, 8, 13, 26, and 65 in the safety set.

Using subjective assessments of clinical images, we will compare the proportion of participants with each degree of skin discoloration in Arm 1 vs Arm 2 in the safety set. We will provide the point estimates together with the Pearson Klopper exact 95% confidence intervals.

6 Primary Analysis Report Contents

All tables, listings, and figures provided in the final report will include totals and percentages by arm and overall unless otherwise specified. At a minimum, the final report will have the following contents.

6.1 Study Entry

- Accrual by site, country, and month
- Summary of late exclusions (number and reasons, by arm)
  - Participants whose screening, entry, and first post-entry visit sputum cultures all fail to grow *Mtb* complex,

    Or
  - Participants whose molecular or drug susceptibility test results demonstrate resistance to INH or RIF after enrollment

6.2 Baseline Characteristics

- Summary of age, sex, gender, country, Karnofsky Score (50-70, 80-100), BMI, HIV Status (stratification factor), advanced disease status determined by chest x-ray (stratification factor), Total CES Depression Scale Score, CD4 count among those HIV positive, viral load among those HIV positive
- Baseline TB diagnostic test results (AFB smear, drug susceptibility tests)
- Baseline lab results for AST, ALT, bilirubin, hemoglobin
- 2x2 table displaying concordance of site classification and central reader classification of advanced disease status by chest x-ray

6.3 Study Status

- Summary of off-study reasons
- Summary of participants last study visit week

6.4 Treatment Status

- Summary of number of participants who did not start study treatment
- Summary of extensions, changes, and restarts of study treatment
- Summary of reasons for permanently discontinuing study treatment
- Summary of number of participants who had an adequate dose of study treatment as defined in the protocol and Section 3.2.1 of this document.

6.5 Adverse Events

- Summary of post-entry reportable adverse events according to Section 7 of the protocol
- Summary of post-entry SAE
- Summary of QTcF at screening, weeks 2, 8, and 13

- Summary of difference in QTcF between screening, and weeks 2, 8, and 13
- Proportion with a maximum occurrence of QTcF ≥ 480 ms and < 500 ms, and ≥ 500 ms

- Proportion with a maximum change from screening of QTcF ≥30 ms and <60 ms, and ≥60 ms, at any time during study treatment
- Summary of Grade 3 or higher post-entry QT prolongation adverse events

6.6 Primary Outcome Measures

- Kaplan-Meier curve of time to stable culture conversion in liquid media, censored at 12 weeks (outcome measure 3.1.1)
- Hazard ratio and 90% two-sided confidence interval for time to stable culture conversion in liquid media (outcome measure 3.1.1)
- Proportions in each arm, and difference in proportions with 90% confidence interval of primary safety outcome at weeks 13, 26, and 65 (outcome measure 3.1.2)

6.7 Secondary Outcome Measures

- Proportions in each arm, and difference in proportions with corresponding confidence interval for outcome measures 3.2.1A and B, 3.2.2, 3.2.5, 3.2.6, 3.2.9, 3.2.10.
- Contrasts that estimate the difference between in mean change in QTcF scores between Arm 1 and Arm 2 from screening to weeks 2, 8, and 13 with corresponding 95% confidence intervals (outcome measure 3.2.3.1)
- Odds ratio and 95% confidence interval from proportional odds model for outcome measures 3.2.3.2 and 3.2.3.3. If proportional odds model does not hold, then difference in proportions for categories QTcF ≥ 480 ms and QTcF ≥ 500 ms between arms with corresponding 95% confidence intervals.
- Kaplan-Meier curves of time to culture conversion in liquid media, and solid media, censored at 26 weeks (outcome measure 3.2.4).
- Hazard ratio and 95% confidence intervals for time to culture conversion in liquid media, and solid media, censored at 65 weeks (outcome measure 3.2.4).
- Hazard ratio and 95% confidence intervals for time to positivity in liquid media at each time point. If proportional hazards does not hold, then we will report log-rank test. Sideby-side box plots of time to positivity (Days) at each time point (weeks 1, 2, 3, 4, 6, 8, 0, 12) (outcome measure 3.2.6)
- Estimates for changes in chest x-ray score in each arm from screening to EOT, and estimate of difference in change with 95% confidence interval (outcome measure 3.2.7)
- Contrasts that estimate the difference with corresponding 95% confidence interval between Arm 1 and Arm 2 for the change in total skin hyperpigmentation color difference from week 0 to each of weeks 8, 13, 26, and 65 at each of the following locations; (1) left cheek, (2) right cheek, (3) forehead, (4) chin, (5) using the location with the maximum change in total skin hyperpigmentation color difference. (outcome measure 3.2.12)
- Contrasts that estimate the difference in mean subjective ‘change in coloration score’ at each of weeks 0, 8, 13, 26, and 65 in Arm 1 vs Arm 2 with corresponding 95% confidence intervals (outcome measure 3.2.13)

  Contrasts that estimate the difference in mean subjective ‘distress to skin coloration’ score at each of weeks 0, 8, 13, 26, and 65 in Arm 1 vs Arm 2 with corresponding 95% confidence intervals (outcome measure 3.2.12)

7 Associated Documents

Attachment 1: Writing Team Roster

Protocol Chair	John Metcalfe, MD, PhD, MPH
Protocol Vice Chairs	Samuel Pierre, MD<br>Kimberly Scarsi, PharmD, MS
DAIDS Clinical Representatives	Melanie Goth
Statisticians	Isabelle Weir, PhD<br>Jorge Leon-Cruz, MS
Pharmacologists	Gary Maartens<br>Elin Svenson
Dermatologist	Leo Shmuylovich

The reports will also be distributed to the TB TSG chair/vice chair and the ACTG network chair. The clinical trials specialist (CTS) and ACTGPublications@mednet.ucla.edu will be notified when the analysis report has been sent.

Attachment 2: Timetable for primary analysis and manuscript preparation

Event	Responsible party	Weeks from primary completion date (PCD)
Pre-closure conference call<br>- Create specimen shipping and testing plan for laboratory data<br>- Check about MTAs<br>- Initiate DTAs	CTS<br>Protocol team<br>NCC<br>LDM	PCD - 26 weeks
Primary completion date (PCD)		PCD
Clinical data entry termination	Sites	PCD + 1 week
Final specimen shipping request of samples to testing laboratories	LDM	PCD + 2 weeks
Initial database clean-up complete, including<br>- DAERS reconciliation complete<br>- TSDV complete	PDM and LDM	PCD + 12 weeks

Laboratory data submitted to Frontier Science	Assay laboratory	PCD + 12 weeks
Clinical database closure/freeze complete/eCRF signoff by site investigators	PDM and Sites	PCD + 15 weeks
SDTM ARMCD populated	SDTM Specialist	PCD + 16 weeks
Processing & QC of laboratory data complete	LDM	PCD + 16 weeks
Complete SDTM on production server	SDTM Specialist	PCD + 18 weeks
Clinical database lock and primary laboratory database lock	Chief Data Manager and LDM Leadership	PCD + 21 weeks
Final SDTM on production server	SDTM Specialist	PCD + 22 weeks
Primary analysis report to protocol Chairs/Writing Team	Protocol statisticians	PCD + 26 weeks
